# Impacts on Human Movement in Australian Cities Related to the COVID-19 Pandemic

**DOI:** 10.3390/tropicalmed8070363

**Published:** 2023-07-14

**Authors:** Daniel J. Weiss, Tara F. Boyhan, Mark Connell, Kefyalew Addis Alene, Paulina A. Dzianach, Tasmin L. Symons, Camilo A. Vargas-Ruiz, Peter W. Gething, Ewan Cameron

**Affiliations:** 1 Telethon Kids Institute, Perth Children’s Hospital, Nedlands, WA 6009, Australia; kefyalew.alene@telethonkids.org.au (K.A.A.); paulina.dzianach@telethonkids.org.au (P.A.D.); tasmin.symons@telethonkids.org.au (T.L.S.); camilo.vargas@telethonkids.org.au (C.A.V.-R.); peter.gething@telethonkids.org.au (P.W.G.); ewan.cameron@telethonkids.org.au (E.C.); 2 School of Population Health, Curtin University, Bentley, WA 6102, Australia

**Keywords:** COVID-19, human movement, Australia, cities, restrictions

## Abstract

No studies have yet examined high-resolution shifts in the spatial patterns of human movement in Australia throughout 2020 and 2021, a period coincident with the repeated enactment and removal of varied governmental restrictions aimed at reducing community transmission of SARS-CoV-2. We compared overlapping timeseries of COVID-19 pandemic-related restrictions, epidemiological data on cases and vaccination rates, and high-resolution human movement data to characterize population-level responses to the pandemic in Australian cities. We found that restrictions on human movement and/or mandatory business closures reduced the average population-level weekly movement volumes in cities, as measured by aggregated travel time, by almost half. Of the movements that continued to occur, long movements reduced more dramatically than short movements, likely indicating that people stayed closer to home. We also found that the repeated lockdowns did not reduce their impact on human movement, but the effect of the restrictions on human movement waned as the duration of restrictions increased. Lastly, we found that after restrictions ceased, the subsequent surge in SARS-CoV-2 transmission coincided with a substantial, non-mandated drop in human movement volume. These findings have implications for public health policy makers when faced with anticipating responses to restrictions during future emergency situations.

## 1. Introduction

Australia has been among the most successful high-income nations at limiting the burden of severe COVID-19 illness amongst its population [1]. While a degree of protection was afforded to Australia by its geographic remoteness, lack of land borders across which infected individuals could travel, low population density, and the timing of the initial spread of SARS-CoV-2 during the Southern Hemisphere summer, the national and state-level responses were instrumental in limiting the public health impact of the pandemic. Governmental restrictions included measures designed to keep infectious individuals out of the community and measures designed to limit transmission of SARS-CoV-2 when it was present. The former included strict international and state border controls, caps on the number of international arrivals, and hotel quarantine systems managed independently by each state or territory; the latter included constraints on human movement and gathering sizes, school and business closures, occupancy limits, and mandates for mask-wearing and working from home. These policies, combined with the willingness of individual Australians to adhere to government directives and receive COVID-19 vaccines once they became available, allowed Australia to avoid the worst impacts of the pandemic. Most restrictions were removed in late 2021 or early 2022 when high vaccination rates were achieved, which led to an expected surge in cases and pressure on hospital systems. Total hospitalizations, direct deaths, and excess mortality [2] were substantial, but well below those experienced by countries that saw widespread community transmission earlier in the pandemic [1].

SARS-CoV-2 was first detected in Australia in January 2020 and limited community transmission was occurring in all Australian capital cities by March of that year [3]. Governmental responses to the first wave were swift and effective, and consisted largely of strict lockdown measures, border closures, and eventually contact tracing of individual cases. These measures virtually eliminated SARS-CoV-2 transmission within Australia by mid-2020 [3] and enabled a gradual lifting of restrictions across the country. Despite this success, the return of Australian residents from abroad presented an unavoidable risk of reintroducing the virus. Australian state and territorial governments responded by instituting arrival caps and hotel quarantine systems for international travelers, which enabled tens of thousands of people to safely enter Australia. However, several reintroductions of SARS-CoV-2 (including variants of concern) nevertheless occurred due to infected travelers returning from abroad, primarily when the virus was accidentally transmitted to quarantine hotel staff, transportation workers, or other quarantined individuals soon to be released. When SARS-CoV-2 escaped quarantine, Australian states instituted lockdown measures, which were effective in many cases at preventing widespread community transmission. Melbourne and Sydney, however, experienced second-wave outbreaks, albeit at different scales, with a much larger outbreak occurring in Melbourne. The state governmental responses to these outbreaks differed considerably, with Victoria (Melbourne) adopting restrictions that were stricter and longer-lasting than those in New South Wales (Sydney).

The widely varying levels of SARS-CoV-2 transmission and differing responses by states make Australia a fascinating context in which to examine population-level behavioral responses to emergency governmental restrictions put in place in response to the pandemic. For example, while Australia has among the lowest COVID-19 mortality rates among high-income countries, and many Australian cities were effectively COVID-free for much of 2020–2021, residents of Melbourne experienced more days with COVID-19-related movement restrictions than any other city on Earth as of October 2021 [4].

The unprecedented coincidence of a global pandemic and the existence of vast datasets enumerating human movement led to a proliferation of research studies exploring how travel patterns were affected by COVID-19 [5], as well as the development of novel tools to visualize and summarize these large movement datasets [6]. Numerous metrics have been used to assess COVID-19-related impacts on people’s lives, including analyses of nation-wide and/or sector-specific mobility during the pandemic [7,8,9,10,11]. Several studies also collected survey data and used them to associate characteristics or opinions of individuals with observed trends in aggregated human mobility data [12,13,14,15,16,17]. A number of human movement studies have focused on Australia [7,8,9,11,14,18], while other publications have had a focus on human mobility in cities elsewhere [15,17,19,20]. None, however, have yet explored high-resolution patterns of movement within Australian cities, particularly within the context of varying governmental restrictions.

This paper builds upon existing research by analyzing spatially and temporally disaggregated human movement data within the context of SARS-CoV-2 transmission, governmental restrictions, and vaccination rates. For this research, we rely on data from the Google COVID-19 Aggregated Mobility Research Dataset (GAMRD), which differs from the COVID-19 Community Mobility Reports used in numerous analyses of changing human mobility during the pandemic [21]. The GAMRD consists of weekly, high-spatial-resolution measurements of human movement captured from November 2019 through January 2022. This dataset contains anonymized mobility flows aggregated over users who have turned on the Location History setting, which is off by default. This is similar to the data used to show how busy certain venues are in Google Maps—helping identify when a local business tends to be the most crowded.

Our analysis is limited to the eight state, territorial, or national capital cities in Australia: Adelaide, Brisbane, Canberra, Darwin, Hobart, Melbourne, Perth, and Sydney. Although few in number, the metropolitan areas of these cities contain two thirds of the total Australian population. While each Australian capital city is unique in its size, character, geography, and political leadership, the consistent nature of the GAMRD supports comparisons between the cities relative to their unique COVID-19 timelines.

In this paper we address the following questions: (1) How did governmental restrictions impact human movement within Australian cities? (2) Did adherence to governmental restrictions during lockdowns attenuate with the duration and/or frequency of the lockdowns? (3) How did human movement patterns change in the absence of government mandates when cases surged within highly vaccinated populations? In answering these questions, we hope that the results of this research will illuminate population-level behavioral responses to infectious disease outbreaks and their subsequent public health interventions.

## 2. Materials and Methods

The datasets obtained or collected for this research capture city-specific data for (1) human movement, (2) restrictions implemented by national, state, or territorial governments, and (3) SARS-CoV-2 transmission and vaccination rates. The human movement data were obtained from the GAMRD and consist of a global dataset of flows between pixels at level 12 of the S2 spherical geometry (https://s2geometry.io/, accessed on 9 July 2023), which has a spatial resolution of approximately 2 × 2 km for Australia. To produce this dataset, machine learning is applied to logs data to automatically segment it into semantic trips [22]. To provide strong privacy guarantees, all trips were anonymized and aggregated using a differentially private mechanism [23] to aggregate flows over time (see https://policies.google.com/technologies/anonymization, accessed on 9 July 2023). This research is done on the resulting heavily aggregated and differentially private data. No individual user data were ever manually inspected, and only heavily aggregated flows of large populations were handled.

All anonymized trips are processed in aggregate to extract their origin and destination location and time. For example, if users traveled from location A to location B within time interval T, the corresponding cell (A, B, T) in the tensor would be n ∓ err, where err is Laplacian noise. The automated Laplace mechanism adds random noise drawn from a zero mean Laplace distribution and yields a (𝜖, δ)-differential privacy guarantee of 𝜖 = 0.66 and δ = 2.1 × 10^−29^ per metric. Specifically, for each week W and each location pair (A, B), we compute the number of unique users who took a trip from location A to location B during week W. To each of these metrics, we add Laplace noise from a zero-mean distribution of scale 1/0.66. We then remove all metrics for which the noisy number of users is lower than 100, following the process described in https://research.google/pubs/pub48778/ (accessed on 9 July 2023), and publish the rest. This yields that each metric we publish satisfies (ε, δ)-differential privacy with values defined above. The parameter 𝜖 controls the noise intensity in terms of its variance, while δ represents the deviation from pure 𝜖-privacy. The closer they are to zero, the stronger the privacy guarantees.

The GAMRD can be conceptualized as a sparse matrix of movements because most potential flows between destination-origin pairs lacked trips from enough users to exceed privacy thresholds. The movement data were available as 115 weekly downloads from November 2019 through January 2022, thereby encapsulating the first two years of the pandemic plus several months to serve as a pre-pandemic baseline. The GAMRD was subset to only include flows within the greater metropolitan areas of the selected Australian cities and consolidated into a single table. The flows were rescaled to range linearly from 1 to infinity such that a flow of 2.0 was twice as large as a flow of 1.0 (i.e., the minimum flow volume in the database of approximately 100 unique individuals moving between two pixels). The travel time between each set of pixels was estimated in minutes using an established method [24,25] and added to the table to serve as a multiplier for incorporating trip length within the analysis. Summary metrics were tabulated to determine the total weekly travel-time-weighted flow (*WF*) volume as

$$WF_{i}=\sum \left({\mathrm{flow}}_{i,j}*{\mathrm{travel}\_\mathrm{time}}_{j}\right)$$

with *i* = the week, and *j* = the associated to-from link in the movement matrix, and the ratio (*WFR*) of each week’s flow relative to the maximum *WF* observed within the 115-week timeseries as

$$WFR_{i}=\frac{WF_{i}}{WF_{max}}$$

The chronology of restrictions for the Australian states and territories was collated from a mixture of media reporting and governmental publications. The resulting database contains the start and end dates for school closures, venue limits, public gathering size limits, business closures, private gathering size limits, movement restrictions, and mask mandates. Restrictions were issued primarily by state and territorial governments and those applicable only to regional areas outside the capital cities were omitted. The restrictions were categorized into five classes (Table 1) to group and analyze movement patterns associated with similar restrictions. The presence of movement restrictions or mandatory business closures, which were typically issued in tandem, were used to define lockdowns. The last category consists of a surge in cases coupled with few restrictions due to high vaccination rates. The start of the city-specific surge periods was defined as the week after October 2021 when each state first set a record for minimum, mean, or maximum daily cases. Note that the transition week of 16–22 March in 2020 was excluded as this week straddled the extremes of the pre-pandemic baseline and the start of the initial lockdown. The SARS-CoV-2 case data and vaccination rates were downloaded from www.covid19data.com.au (accessed on 9 July 2023). All cases, including those identified in quarantined travelers, were included in this analysis to avoid confusion related to inconsistent definitions in the reported data among the states and territories. Vaccination coverage was defined as two doses with an approved vaccine and the percentage was relative to the total population, including young children who were ineligible for vaccination as of January 2022.

For each week in the movement database, each city contained thousands of flows between S2 pixels with which to assess the impacts of COVID-19 on Australian cities. All flows were included in comparisons of the different lockdown classes and the tabulation of the descriptive statistics presented, but the number of flows present within the movement database was too large to map effectively. As such, only flows that were ever within the top 1% of flows within a city, on any week of the 115-week timeseries, were included within the maps. Furthermore, due to the size of the level 12 S2 pixels relative to human movements, many flows in the database were intra-pixel (i.e., had a matching origin and destination) and were thus represented on our maps as points rather than lines.

We use visualizations, summaries of the data, and simple statistical tests to address the research questions posed in the introduction. Chronologies juxtaposing flow volumes relative to governmental restrictions, maps, and plots of distance vs. flow volume illustrate how human movement was impacted during the pandemic. Generalized linear models (GLMs) relating the flow volumes during lockdown periods to the length of lockdown and number of lockdown periods are used to assess adherence to governmental restrictions. Due to the limited number of observations of years with and without surges in the absence of government restrictions, only visualizations are used to illustrate the impacts on human movement of SARS-CoV-2 case surges relative to periods without restrictions.

## 3. Results

Results of this analysis consist primarily of plots and maps. Due to the large number of visualizations, only maps for selected cities are shown in the manuscript. However, corresponding graphics for all cities are available within the Supplementary Information.

### 3.1. COVID-19 Chronologies

**Figure 1:**
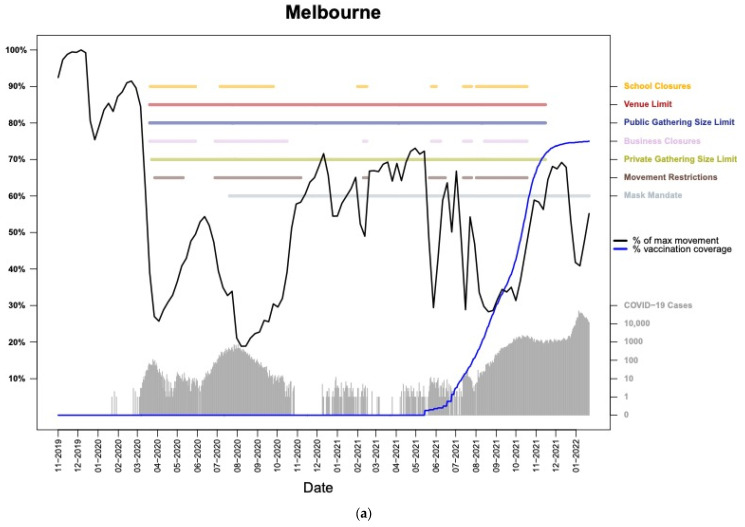

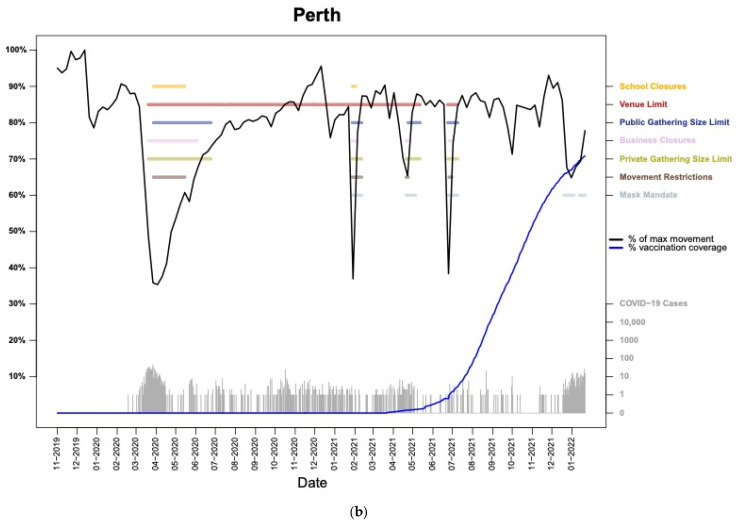
The movement, restrictions, vaccinations, and incidence chronology for Melbourne (**a**) and Perth (**b**). The black line represents movement within the city relative to the most movement observed within the timeseries (i.e., typically the pre-pandemic peak movement week). Cases are presented in gray and displayed on a logarithmic scale (values displayed on right *Y*-axis). The presence of restrictions is indicated by the colored horizontal lines (labels displayed on right *Y*-axis). Comparable plots for the other seven cities are presented in the Figure S1 of the Supplementary Information.

**Figure 2:**
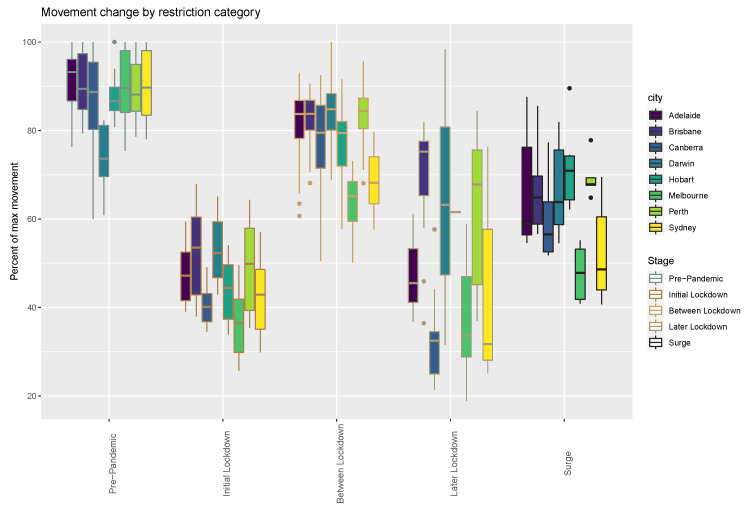
Declines in human movement flow volume by city coincident with each restriction category during the COVID-19 pandemic.

Chronologies of movement rates illustrate city-specific COVID-19 chronology plots that juxtapose human movement volume, governmental restrictions, and SARS-CoV-2 transmission and vaccination rates (Figure 1 and Figure S1). As expected, the lockdown measures consisting of movement restrictions and/or business closures caused the weekly flow volume to plummet both during the initial lockdown phase and in subsequent lockdowns (Table 2, Figure 2). The movement volumes in the cities had similar magnitudes of change by restriction type, although the changes in flows were less consistent for the later lockdowns.

### 3.2. Spatial Patterns of Movement Changes

**Figure 3:**
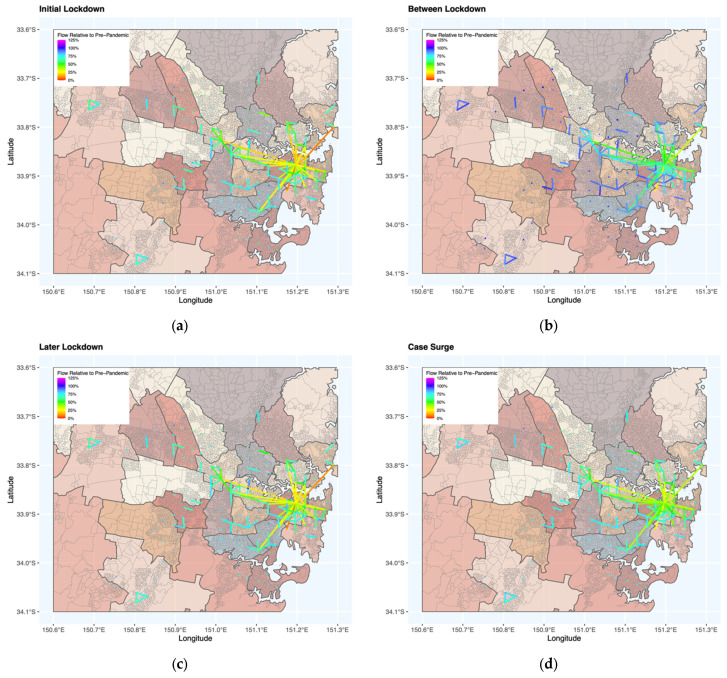
Movement maps for Sydney during pandemic stages relative to a pre-pandemic baseline for (**a**) the initial lockdown period, (**b**) periods without movement restrictions, (**c**) later lockdowns, and (**d**) surges in cases within a highly vaccinated population. For interpretability, only flows that were within the top 1% of flow volume on any week within the timeseries are mapped. Flows depicted with points rather than lines are indicative of movements within the 2 km pixels. Maps for all eight cities are presented in Figure S2 of the Supplementary Information.

**Figure 4:**
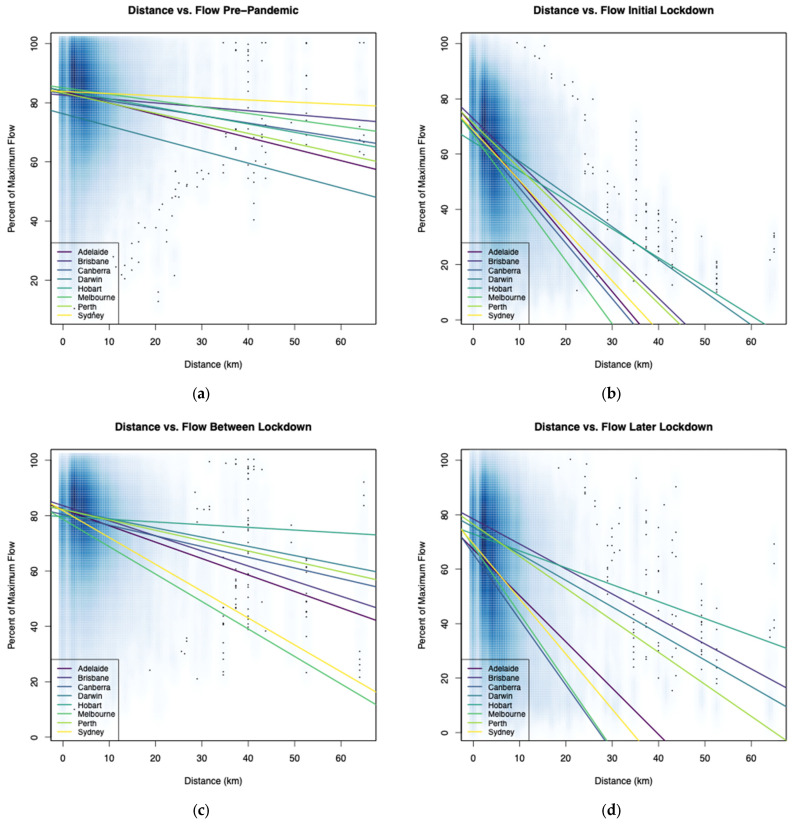

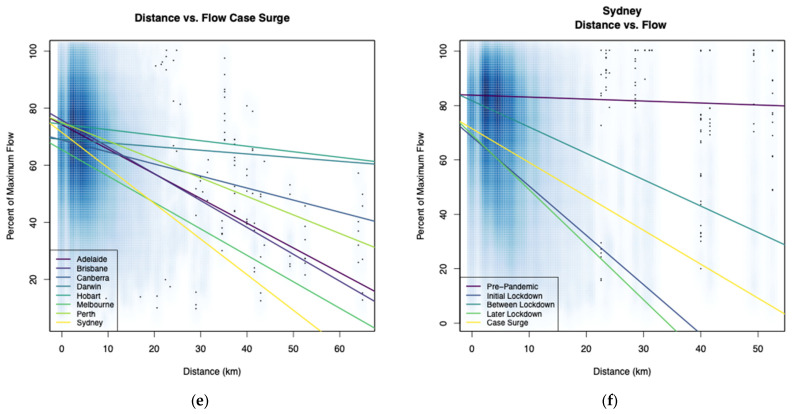
Linear trends in human movement flow volume by city for each COVID-restriction type shown on density plots (in blue) that illustrate the frequency of observed movements in each distance category. The inverse relationship between distance and flow volume is present in all stages (**a**–**e**), but more pronounced when harsher restrictions were implemented. Variability among cities may reflect features of the lockdowns such as limits on distance traveled from home and/or unique traits in cities and their populations. Panel (**f**) is a counter example of all lockdown categories for a single city (Sydney). Comparable plots for other cities are available in Figure S3 of the Supplementary Information.

As lockdown measures were implemented or removed, patterns of human movement shifted accordingly. In Australian cities, the number of people observed moving within or between S2 pixels declined after the onset of the pandemic in early 2020, including the times with few restrictions (Figure 3 and Figure S2). As expected, these declines were more pronounced during periods with movement restrictions and/or business closures. The relative reduction in flow volumes varied by trip distance, with steeper declines for longer trips than short ones, and this relationship was more pronounced with increased restrictions and thus lower levels of overall human movement. This observation is interpretable as people restricting their movements largely to short trips of necessity such as getting groceries, although in some instances government mandates also limited the distance people could travel from home without an approved reason. This additional aspect of the restrictions may explain the disparity in the relative declines in flow vs. distance by restriction category as well as city (Figure 4 and Figure S3).

### 3.3. Movement Reductions during the Case Surges

**Figure 5:**
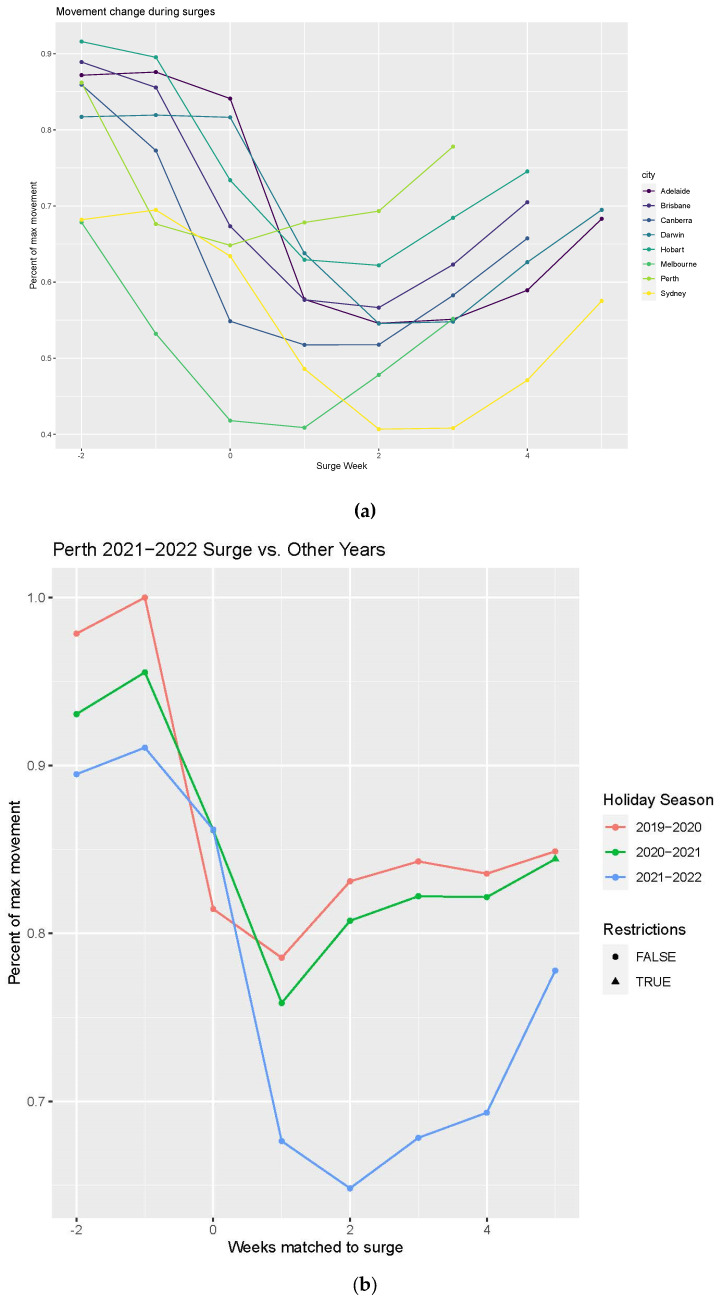
Declines in human movement flow volume, relative to the peak observed weekly flow, coincident with the late pandemic case surges amongst a heavily vaccinated population and without restrictions (**a**). The non-mandated change in human behavior, as observed by movement flows, reflects a population-level response to COVID circulating freely and widely in the cities for the first time. These decreases exceeded the typical seasonal declines observed in the absence of community transmission of COVID (**b**). City-specific plots for surges relative to anniversary weeks in other years are shown in Figure S4 of the Supplementary Information.

In late 2021, the arrival of the Omicron variant of COVID-19, coupled with the removal or reduction of COVID restrictions due to high vaccination rates, led to asynchronous surges in cases in all Australian capital cities. In each city, the surge in cases coincided with a drop in flow volume (Figure 5a), but a confounding effect in interpreting this relationship is the typical annual reduction in flow volume that accompanies the Christmas holiday season. However, when compared to reductions in flow volume relative to COVID-free Christmas seasons, the GAMRD data suggest that the surges in cases led to declines in human movement that far exceeded seasonal declines in other years (Figure 5b). While we did not attempt to determine the underlying cause of the drop in human movement observed during the surge phase, we suspect it was due to a combination of individual apprehension (i.e., reducing travel to limit exposure), non-government-mandated shifts to working from home, and a decrease in the number of people travelling due to self-quarantine of individuals ill with COVID or having been exposed to the virus by someone in their household.

### 3.4. Addressing the Research Questions

(1) How do governmental restrictions impact human movement within Australian cities?

Governmental restrictions, in particular limits on movements and business closures, reduced the volume of human movement evident in the GAMRD considerably. During lockdowns, the average weekly travel time-weighted flow was 49% flow (range of 33.2 to 69.2 of the corresponding city’s maximum observed) of pre-pandemic maximum (Table 2, Figure 2). Additionally, longer movements were reduced more than shorter movements, suggesting individuals generally stayed closer to home during these periods.

(2) Does adherence to governmental restrictions during lockdowns attenuate with the duration and frequency of the lockdowns?

Governmental restrictions were unambiguously effective at reducing human movement within Australian cities. However, the visualizations suggest that as individual lockdowns wore on, flow volumes gradually increased. To support this observation, we fit a simple linear model and confirmed a positive slope (beta = 0.0106 (0.007–0.013)) between lockdown week and the weekly flow volume minus the minimum flow of the corresponding lockdown period. The phenomenon of increasing movement as lockdowns wore on was apparent during the lengthy initial lockdowns as well as most other lockdowns that were more than two weeks in length. Short lockdowns (aka “snap lockdowns”), in contrast, tended to have a pronounced impact on human movement, but ended so quickly that there was not time for that impact to attenuate. While the impact of restrictions on human movement waned with the duration of lockdown, in the context the approximately two-year COVID-19 pandemic period in Australia, the number of lockdown periods did not appear to decrease the public’s willingness to follow government restrictions that led to reduced movement. This observation was supported by non-significant relationships between lockdown number and minimum flow volume per lockdown.

(3) How do human movement patterns change in the absence of government mandates when cases surged within highly vaccinated populations?

The surge in cases within cities with high levels of vaccination appeared to cause a drop in movement in the absence of restrictions. These drops were more modest than those associated with movement restrictions and/or business closures, but atypical of any other period in the movement timeseries. An important consideration in this assessment is the cooccurrence of surges in cases with the Christmas holiday season, which is a typical period of reduced movement. However, compared to pre-pandemic or between lockdown periods (i.e., unrestricted movement and no business closures), the surge phase had substantially lower levels of human movement.

## 4. Discussion

Existing research on the impacts to human movement patterns of COVID-19 and its associated public health responses in Australia has relied upon temporally and/or spatially aggregated data [7,8,9,11]. While illustrative of important changes occurring in Australia, in particular in sector-specific movements that our approach could not evaluate, the datasets used in these analyses precluded exploration of fine-scale shifts in the magnitude, spatial pattern, and timing of human movements. Examples of similar, high-resolution movement analyses in cities [17,20] were conducted in countries that had COVID-19 present throughout the pandemic and generally adopted less aggressive interventions to limit the transmission of SARS-CoV-2 than those implemented in Australia. As such, alternative studies could not analyze population movement responses between periods with and without SARS-CoV-2 transmission. Likewise, studies based in countries that had large numbers of COVID-19 cases prior to widespread vaccination could not examine how population movement was affected by unrestricted SARS-CoV-2 transmission that occurred for the first time within a predominately vaccinated society.

There are several limitations associated with this work that should be considered when interpreting results. The weekly temporal resolution of the GAMRD resulted in multiple categories occurring in weeks when restrictions were implemented or discontinued (i.e., transition weeks that had different restrictions on different days). Likewise, the weekly resolution was inadequate for fully exploring snap lockdowns, some of which only lasted a few days. Another consideration is typical fluctuations in movement patterns and volume around national holidays and periods when schools are closed (e.g., Christmas, New Year, and Easter), not all of which occurred within in the pre-pandemic baseline. While we assume the pre-pandemic period to be a stabile baseline for the number of smartphone users, total flow volumes, and spatiotemporal movement patterns, some results suggest there were shifts in the number of users captured within the GAMRD that will influence the results. For example, in Darwin, the maximum weekly movement occurred in July 2021 rather than during the pre-pandemic period.

Additionally, these results should be interpreted in light of several important limitations related to the GAMRD dataset. First, GAMRD is limited to smartphone users who have opted into Google’s Location History feature, which is off by default. These data may not be representative of the population as a whole, and furthermore their representativeness may vary by location. Importantly, these limited data are only viewed through the lens of differential privacy algorithms, specifically designed to protect user anonymity and obscure fine detail. Moreover, comparisons across rather than within locations are only descriptive since these regions can differ in substantial ways.

There were also spatiotemporal limitations related to the restrictions dataset, including nonuniform implementation of lockdowns across cities. For example, in Sydney in December 2020, business closures and movement restrictions were only implemented in the Northern Beaches area in response to a localized SARS-CoV-2 outbreak. Similarly, the categorization used to determine lockdowns fails to capture nuances, such as easing but not eliminating movement restrictions, that may explain the gradual increase in movement observed during extended lockdowns. More broadly, this analysis only included cities in Australia, which had an atypical experience with the pandemic compared to other nations. As such, cultural and geographic factors should be considered closely when extending lessons learned from the Australian to other contexts.

Lastly, there were also limitations related to the epidemiological data, as the publicly available data for some states included imported cases while other states only included SARS-CoV-2 cases acquired locally. Another caveat to consider is that the epidemiological data were for the state or territory rather than just the capital city. However, we assume the state or territorial case and vaccination rates to be representative of the cities given the concentrated distribution of the Australian population. Lastly, the SARS-CoV-2 case count timeseries are likely be imperfect, particularly for the surge phases, as many people may have gone untested or failed to report positive tests taken at home.

Despite the limitations, this research is unique in bringing together epidemiological, governmental, and high-spatial-resolution datasets of human movement datasets to demonstrate how populations of medium to large cities responded to both epidemiological conditions and governmental policies. Unlike previous research, which was limited to aggregated movement data, this project characterizes where, when, and how human movement changed in response to lockdowns. The key findings of this research include the following: (a) The aggregated movement among the populations of the cities dropped dramatically in response to movement restrictions and/or business closures; (b) lockdown measures remained highly effective at reducing human movement despite occurring repeatedly over the course of the pandemic; and (c) as individual lockdowns wore on, however, their impact on human movement declined, though never to the levels of unrestricted times. Taken together, these findings suggest that, when possible, short-but-frequent lockdowns are preferable to lengthy lockdowns aimed at reducing human movement. Lastly, we found that longer trips were reduced much more than shorter ones when lockdowns were in place, which is intuitive as people continued to make short trips for necessities while forgoing unnecessary trips farther afield.

To improve public health responses during future pandemics, additional research should explore the causal mechanisms driving individuals to change their behaviors, including behavioral changes beyond just movement. While broad governmental restrictions clearly had an impact on human movement, several findings emerged from this research that warrant further examination. These include: (a) During lockdown periods, levels of movement dropped considerably but remained well above zero and tended to rise as the lockdowns wore on. While some level of movement remained necessary (e.g., getting groceries, essential workers commuting, etc.), some of these movements were likely the result of individuals flouting the rules. Understanding the factors which influence the decision of an individual to not comply with movement restriction directives may improve our ability to create more effective messaging in future public health emergencies. (b) Conversely, we found evidence that there were individuals self-limiting their own movement in the absence of restrictions. For example, most cities had reduced levels of human movement, relative to pre-pandemic norms, even between lockdown periods, and there were consistent drops in movement during the surge phases despite an absence of restrictions. These findings point to the efficacy of non-governmental action and, once studied, could potentially be replicated in the future to reduce human movement in the service of limiting disease transmission without measures that some consider infringements on civil liberties.

In conclusion, the results presented here illustrate the utility of combining diverse datasets for improving our understanding of human behavior changes in response to the COVID-19 pandemic and related government actions. While the analysis was limited to Australian cities, the consistency of responses to lockdown measures despite the varied pandemic experiences and character of the cities suggests that the findings may have utility in future public health emergencies in other locations and contexts.

## Figures and Tables

**Table 1:** Descriptions of the five pandemic-related restriction categories.

Category	Description
Pre-Pandemic	All weeks prior to 15 March 2020
Initial Lockdown	All weeks between 23 March and 17 May 2020
Between Lockdown	All weeks without movement restrictions and/or business closures after initial lockdown and prior to the late surge
Later Lockdown	All weeks with movement restrictions and/or business closures after initial lockdown and prior to the late surge
Surge	Late-stage outbreak within a heavily vaccinated society and without lockdown restrictions

**Table 2:** Mean weekly movement by city and restriction status. Percentage are relative to the maximum observed weekly movement (typically pre-pandemic) for each city within the database.

City	Pre-Pandemic	Initial Lockdown	Between Lockdowns	Later Lockdown	Surge
Adelaide	90.9	47.9	81.9	47.8	66.6
Brisbane	90.0	52.8	82.7	69.2	66.6
Canberra	87.0	40.6	77.9	33.2	59.9
Darwin	73.9	53.2	84.0	64.4	67.0
Hobart	87.3	43.8	77.2	61.6	71.8
Melbourne	89.8	36.5	64.9	36.2	47.8
Perth	89.6	49.3	83.5	62.1	69.5
Sydney	90.0	42.5	68.9	40.9	52.5
All Cities	87.3	45.9	78.7	44.8	62.8

## Data Availability

The Google COVID-19 Aggregated Mobility Research Dataset used for this study is available with permission from Google LLC. The governmental restrictions database from this analysis are available for download at https://data.malariaatlas.org/downloads/COVID_restrictions_Australia.xlsx (accessed on 9 July 2023).

## Supplementary Materials

The following supporting information can be downloaded at: https://www.mdpi.com/article/10.3390/tropicalmed8070363/s1. Figure S1. The movement, restrictions, vaccinations, and incidence chronology for all state and territorial capitals in Australia. The black line represents movement within the city relative to the most movement observed within the timeseries. Cases are presented as in gray and displayed on a logarithmic scale. Figure S2. Movement maps for cities during pandemic stages relative to a pre-pandemic baseline for (a) The initial lockdown period, (b) Periods without movement restrictions, (c) Snap lockdowns, and (d) Surge in cases within a highly vaccinated popu-lation. For interpretability, only flows that were within the 1% of flow volume on any week within the timeseries are mapped. Figure S3. Declines in human movement flow volume by city for all lockdown categories for a single city. The lines represent the linear trends for all weeks within each category. Figure S4. Declines in human movement flow volume coincident with the late pandemic case surges amongst a heavily vac-cinated population and without restrictions compared to observed holiday movement volumes in other years. Anniversary weeks are aligned for each plot.
