# Supplementary material for: Impacts on Human Movement in Australian Cities Related to the COVID-19 Pandemic
Source: Trop Med Infect Dis. 2023 Jul 14;8(7):363. doi: 10.3390/tropicalmed8070363 (PMC10385321; doi:10.3390/tropicalmed8070363)
Supplement: Supplementary file 1 [file tropicalmed-08-00363-s001.zip › tropicalmed-2424623-supplementary.pdf]

Tropical Medicine and Infectious Disease

MDPI

Supplementary Materials:

Impacts on Human Movement in Australian Cities Related to the COVID-19 Pandemic

Daniel J. Weiss [1,2,*], Tara F. Boyhan [2], Mark Connell [2], Kefyalew Addis Alene[1,2], Paulina A. Dzianach [1], Tasmin L. Symons [1], Camilo A. Vargas-Ruiz [1], Peter W. Gething [1,2] and Ewan Cameron [1,2]

1 Telethon Kids Institute, Perth Children's Hospital, Nedlands WA 6009, Australia
2 Curtin University, School of Population Health, Bentley WA 6102, Australia
* Correspondence: Daniel.Weiss@telethonkids.org.au

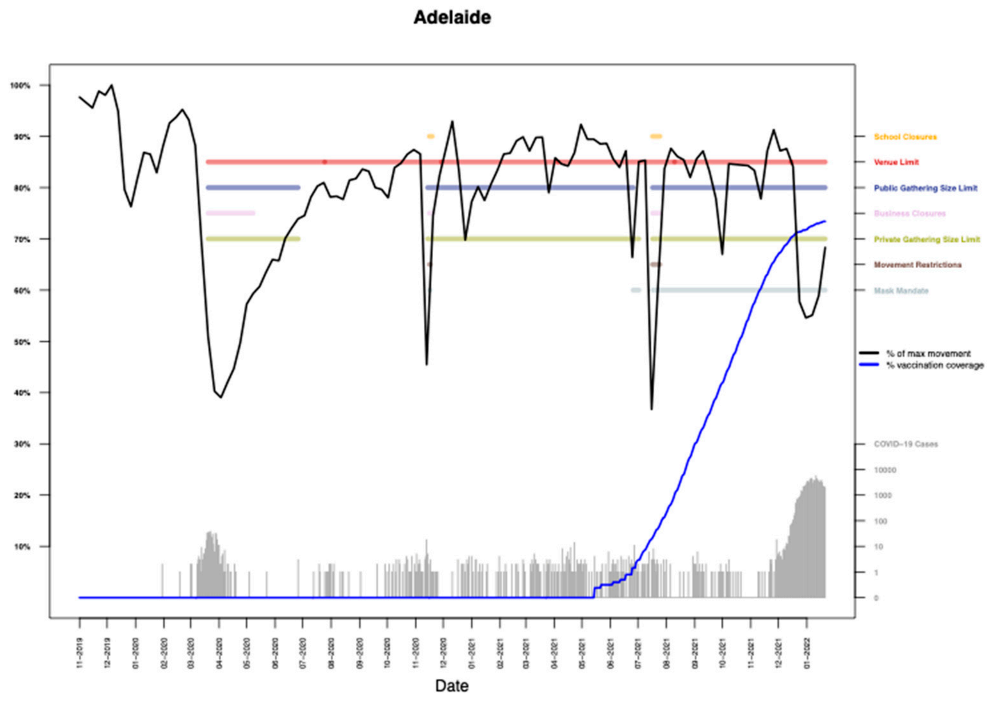

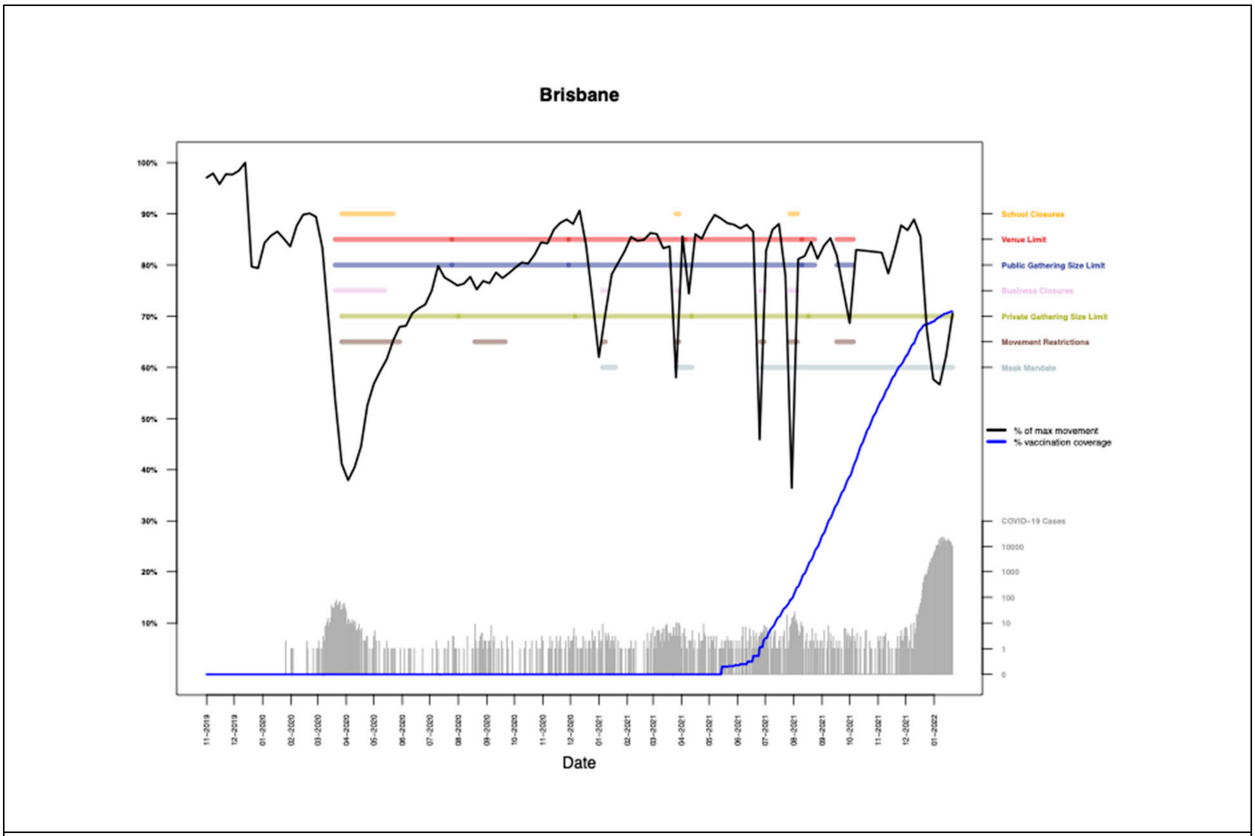
Brisbane
School Closures
Venue Limit
Public Gathering Size Limit
Business Closures
Private Gathering Size Limit
Movement Restrictions
Mask Mandate
% of max movement
% vaccination coverage
COVID−19 Cases
Date

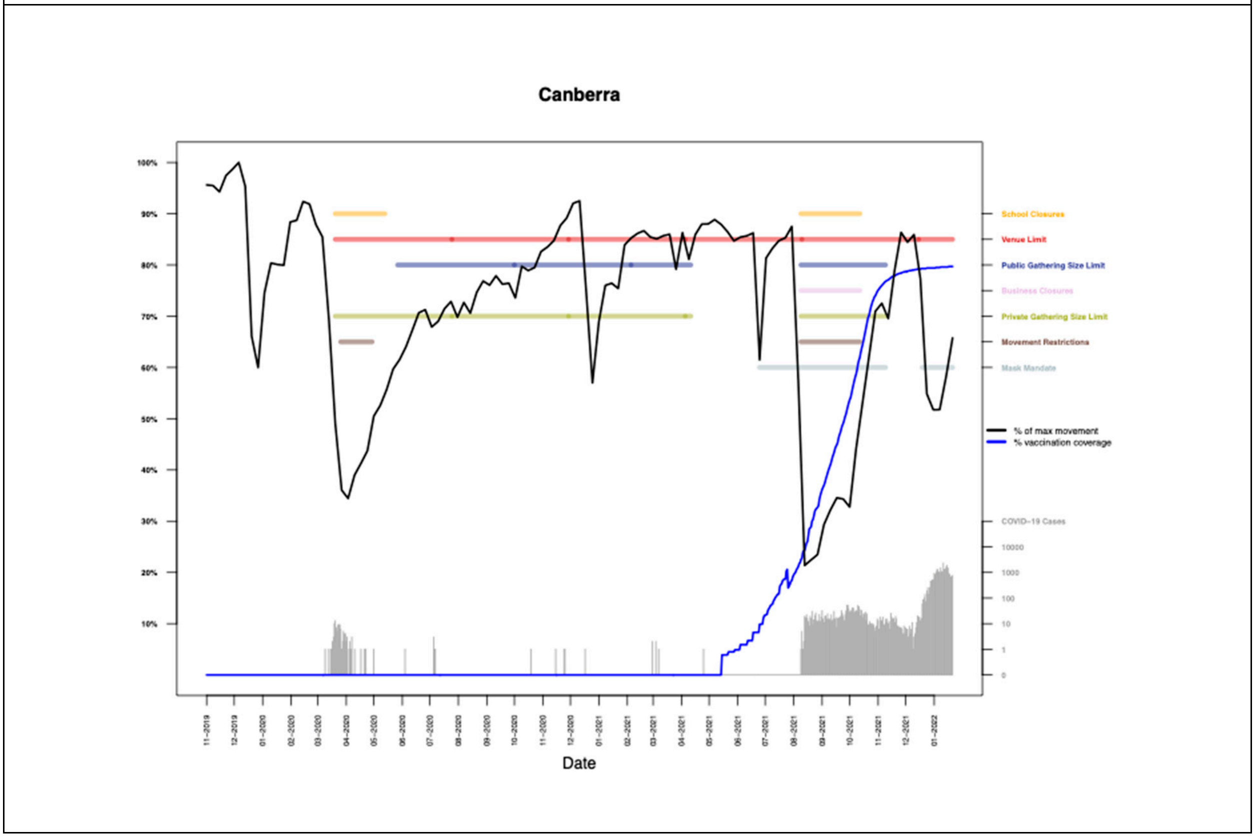
Canberra
School Closures
Venue Limit
Public Gathering Size Limit
Business Closures
Private Gathering Size Limit
Movement Restrictions
Mask Mandate
% of max movement
% vaccination coverage
COVID−19 Cases
Date

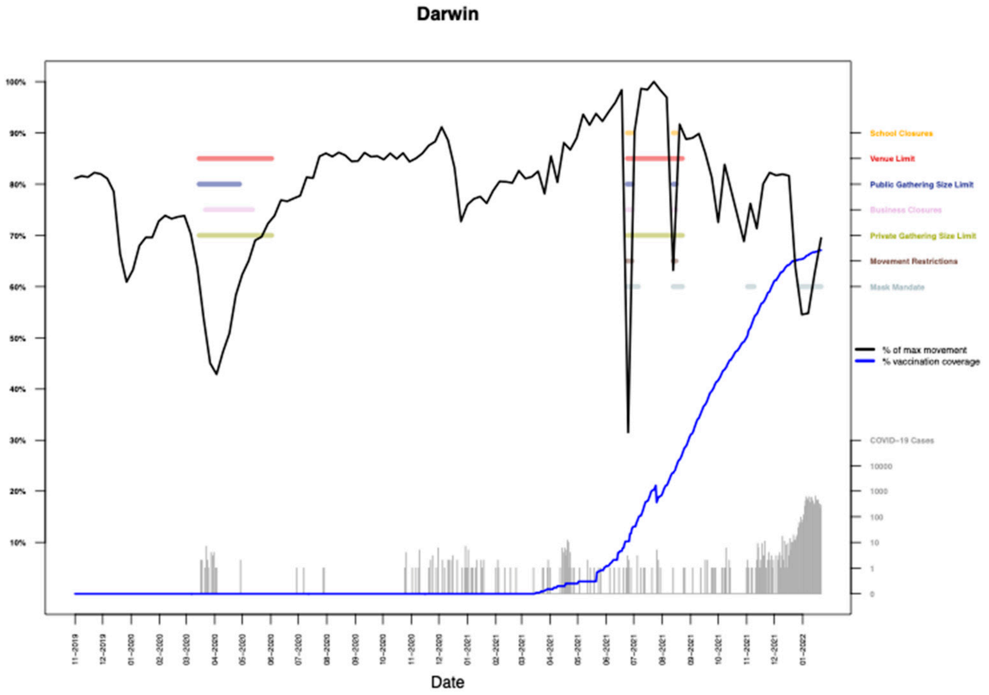
Darwin
School Closures
Venue Limit
Public Gathering Size Limit
Business Closures
Private Gathering Size Limit
Movement Restrictions
Mask Mandate
% of max movement
% vaccination coverage
COVID-19 Cases
Date

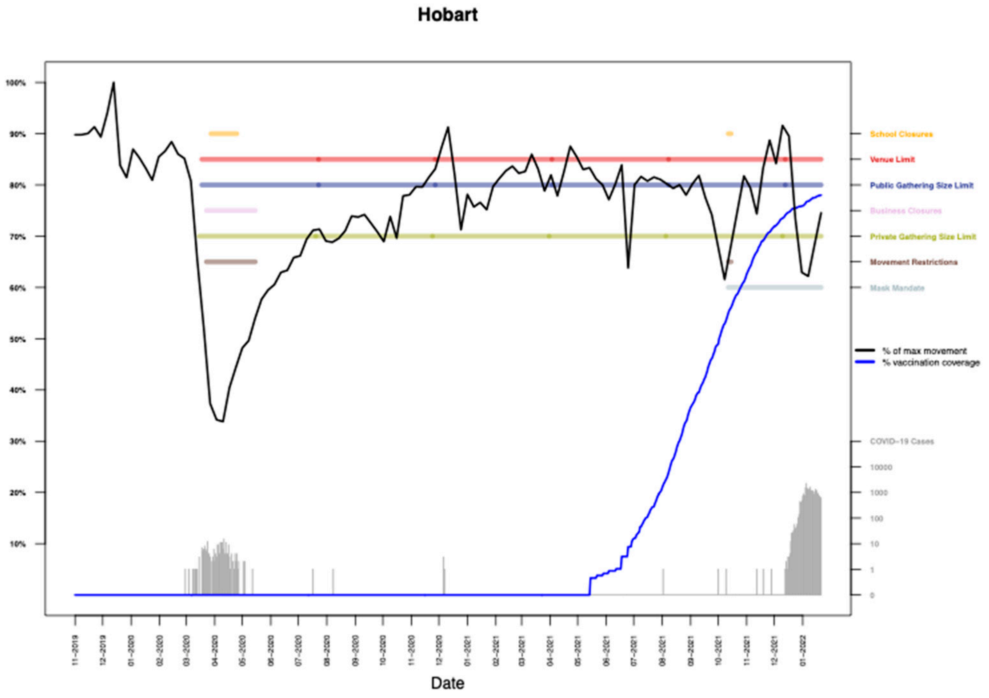
Hobart
School Closures
Venue Limit
Public Gathering Size Limit
Business Closures
Private Gathering Size Limit
Movement Restrictions
Mask Mandate
% of max movement
% vaccination coverage
COVID-19 Cases
Date

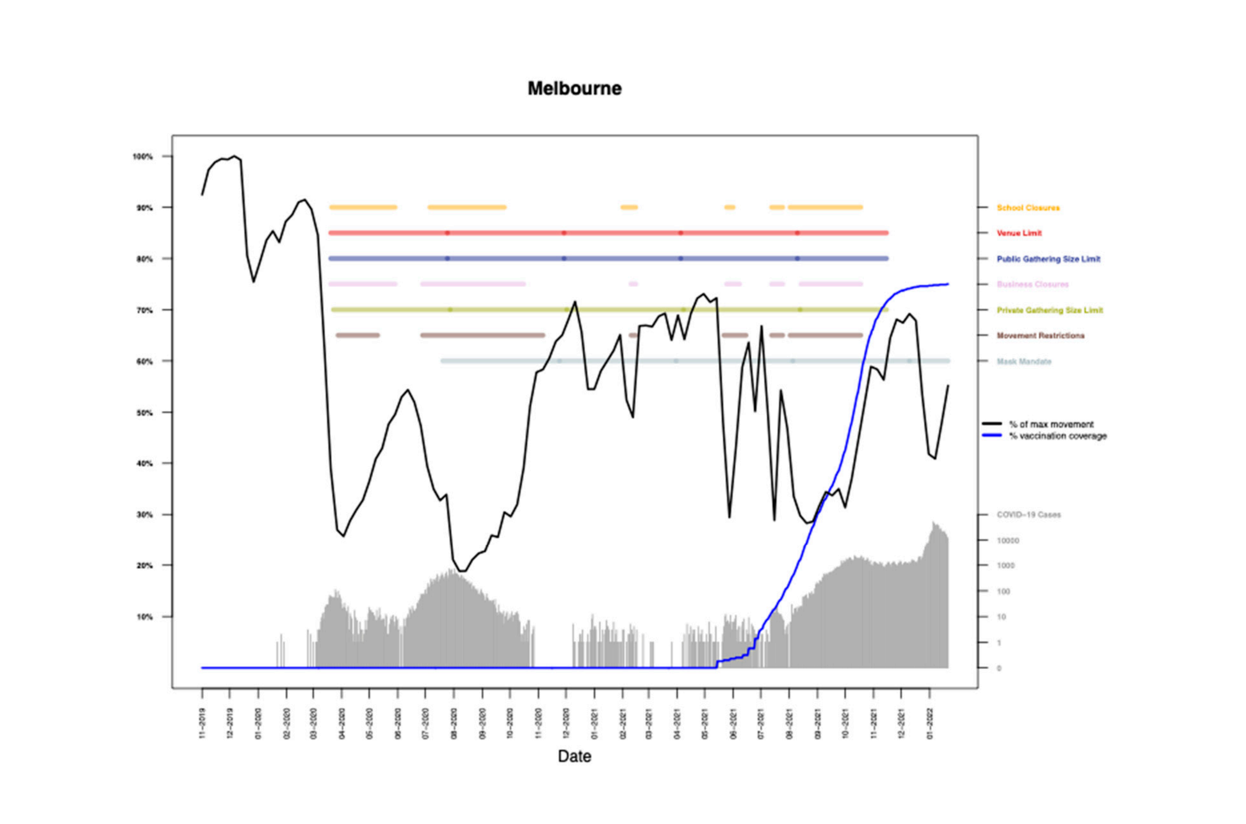
Melbourne
School Closures
Venue Limit
Public Gathering Size Limit
Business Closures
Private Gathering Size Limit
Movement Restrictions
Mask Mandate
% of max movement
% vaccination coverage
COVID−19 Cases
Date

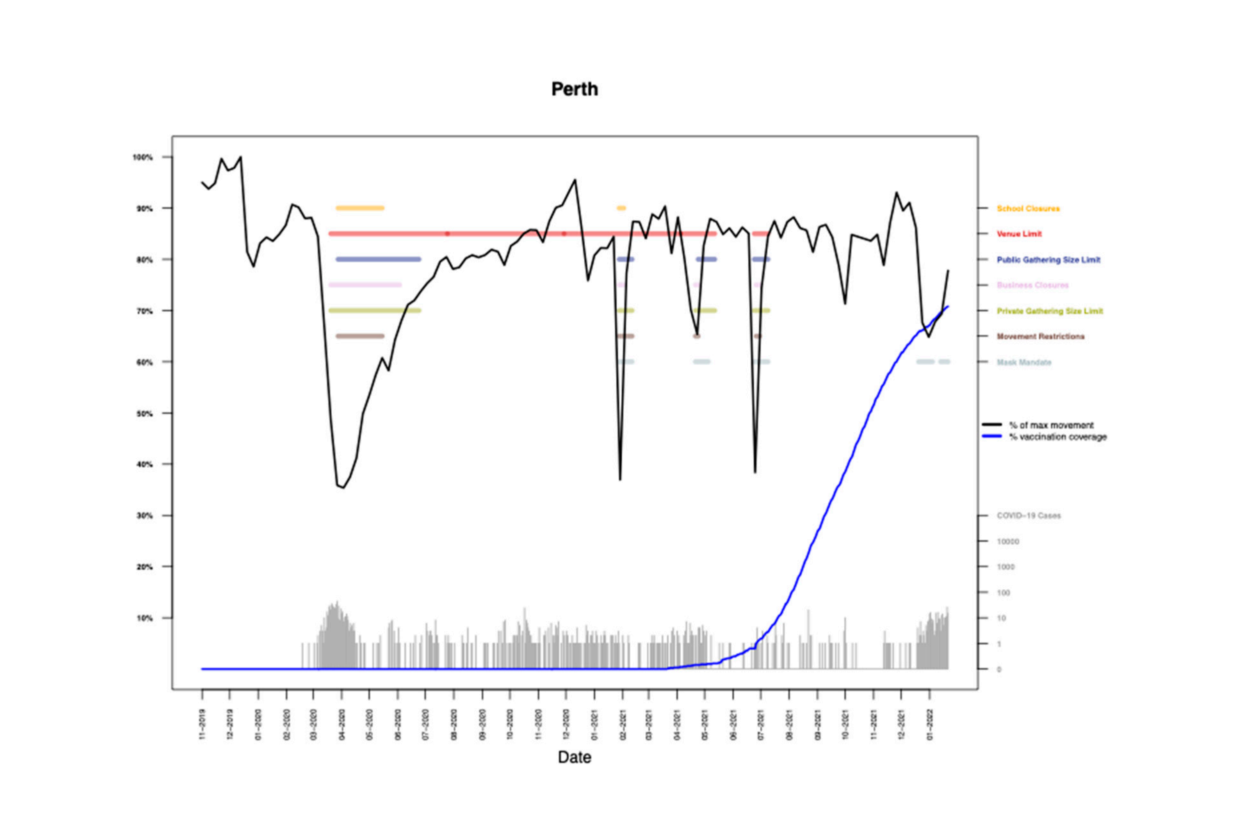
Perth
School Closures
Venue Limit
Public Gathering Size Limit
Business Closures
Private Gathering Size Limit
Movement Restrictions
Mask Mandate
% of max movement
% vaccination coverage
COVID−19 Cases
Date

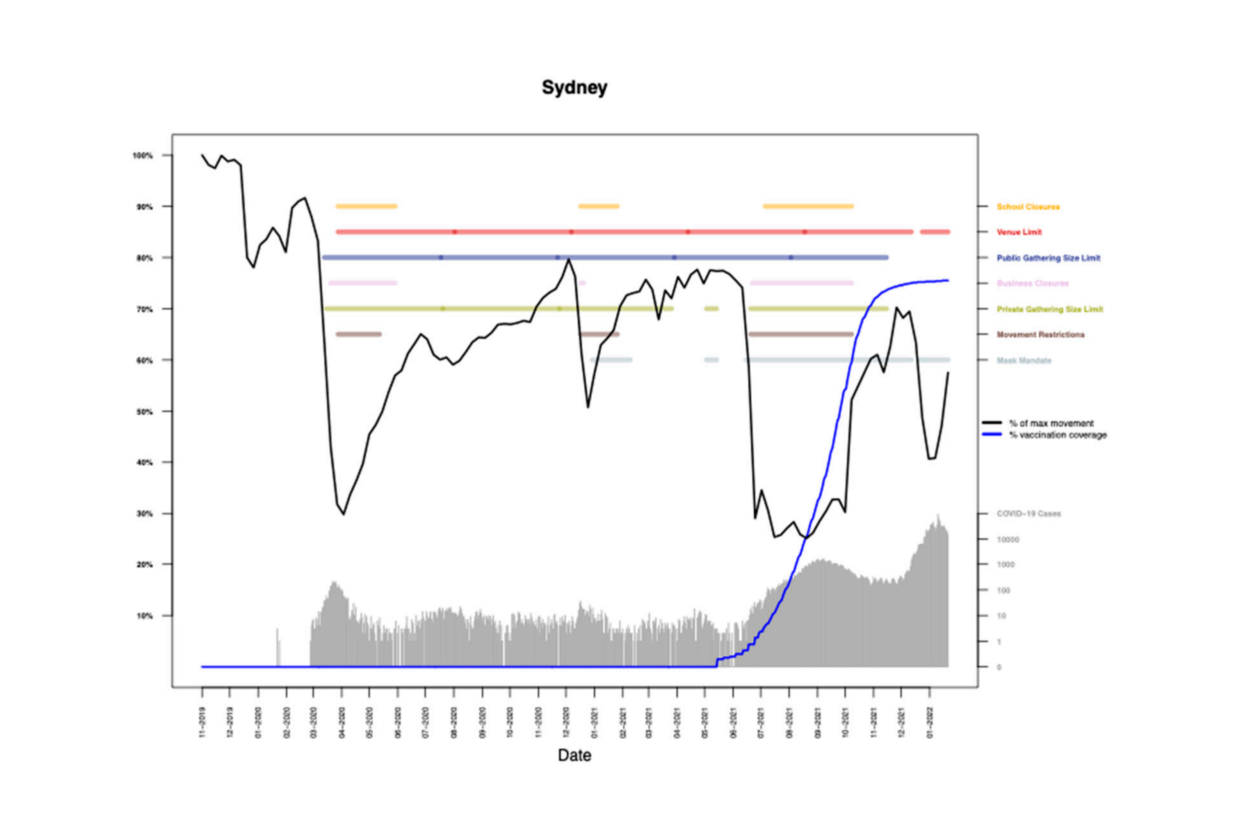

Figure S1. The movement, restrictions, vaccinations, and incidence chronology for all state and territorial capitals in Australia. The black line represents movement within the city relative to the most movement observed within the timeseries. Cases are presented as in gray and displayed on a logarithmic scale.

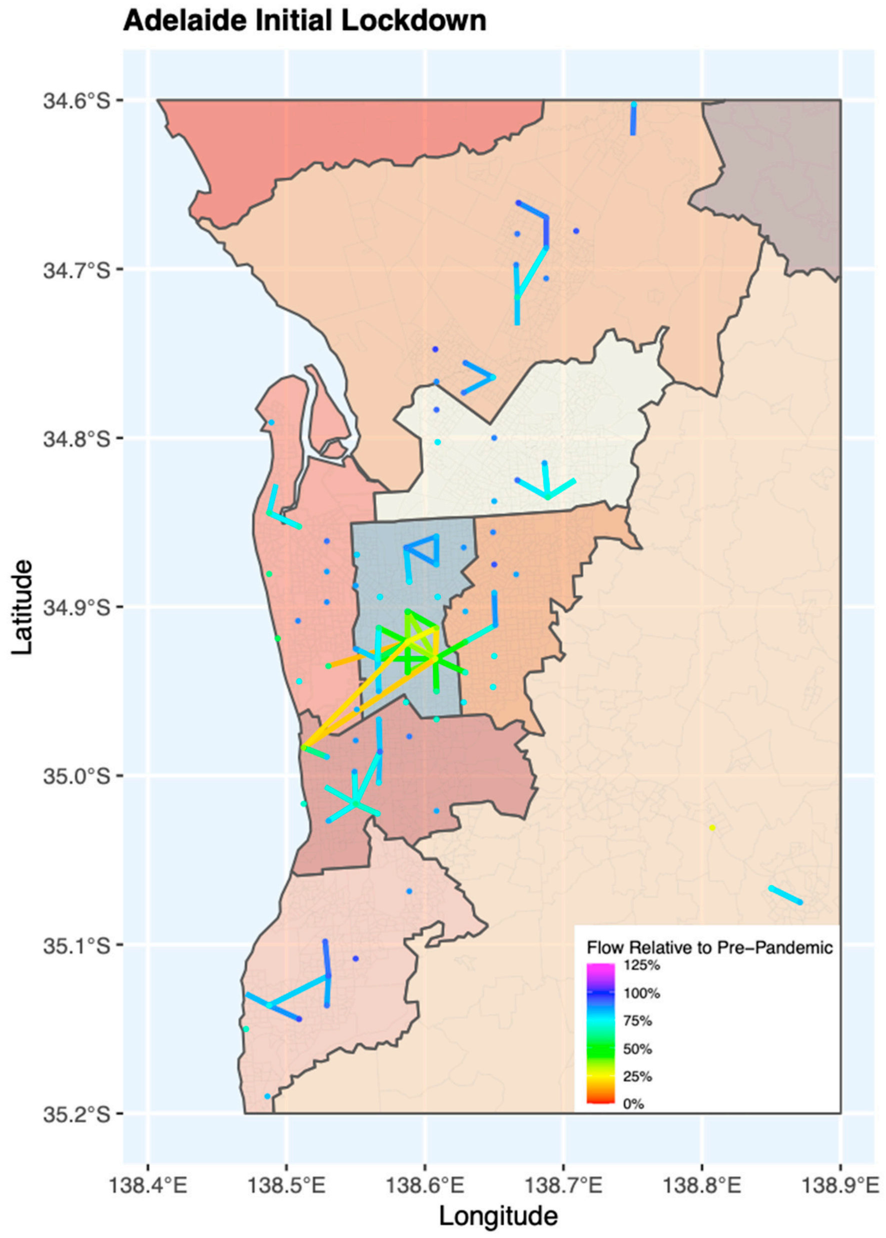
Adelaide Initial Lockdown
34.6°S
34.7°S
34.8°S
34.9°S
35.0°S
35.1°S
35.2°S
Latitude
138.4°E
138.5°E
138.6°E
138.7°E
138.8°E
138.9°E
Longitude
Flow Relative to Pre–Pandemic
125%
100%
75%
50%
25%
0%

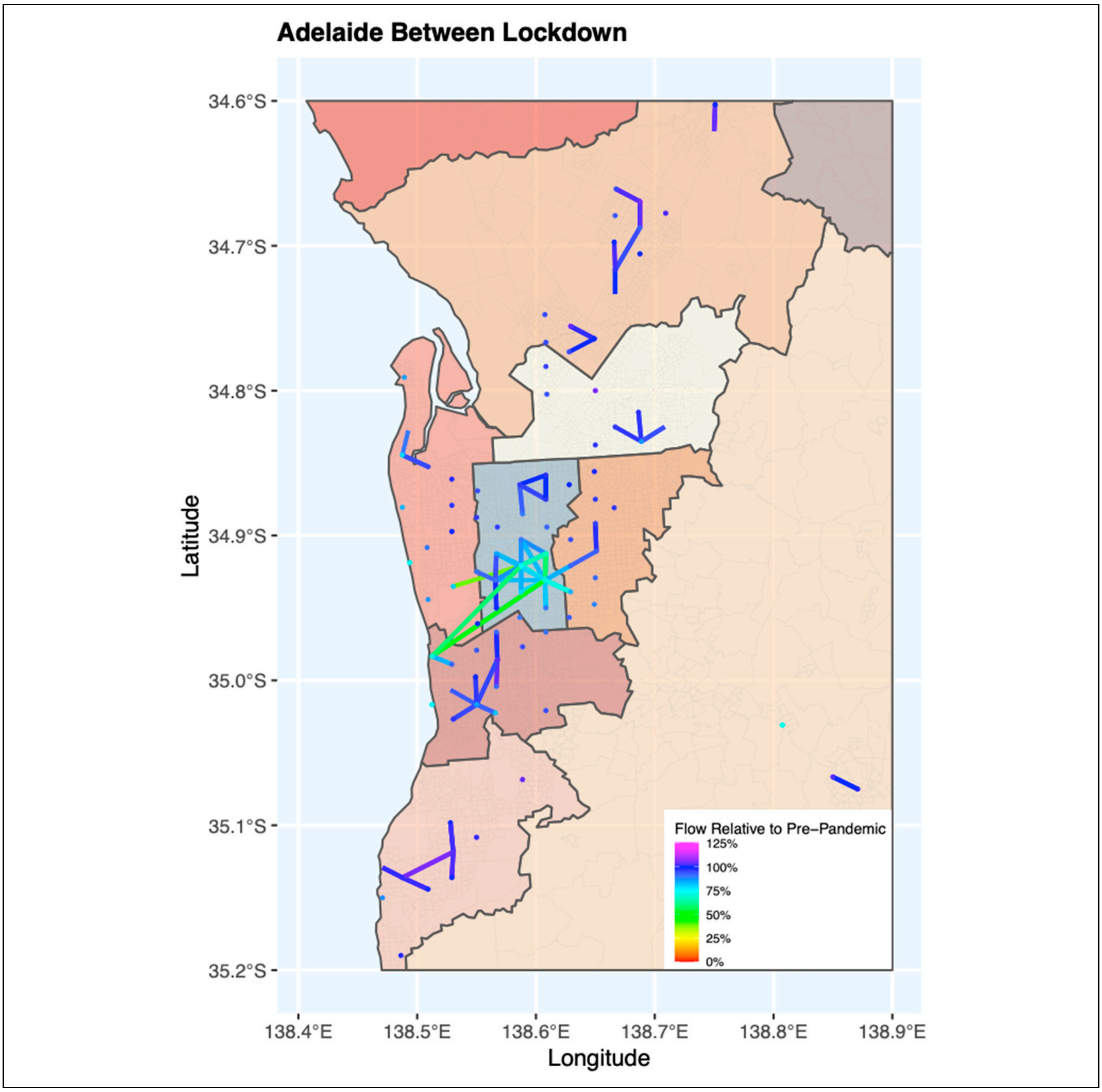
Adelaide Between Lockdown
34.6°S
34.7°S
34.8°S
34.9°S
35.0°S
35.1°S
35.2°S
Latitude
138.4°E
138.5°E
138.6°E
138.7°E
138.8°E
138.9°E
Longitude
Flow Relative to Pre−Pandemic
125%
100%
75%
50%
25%
0%

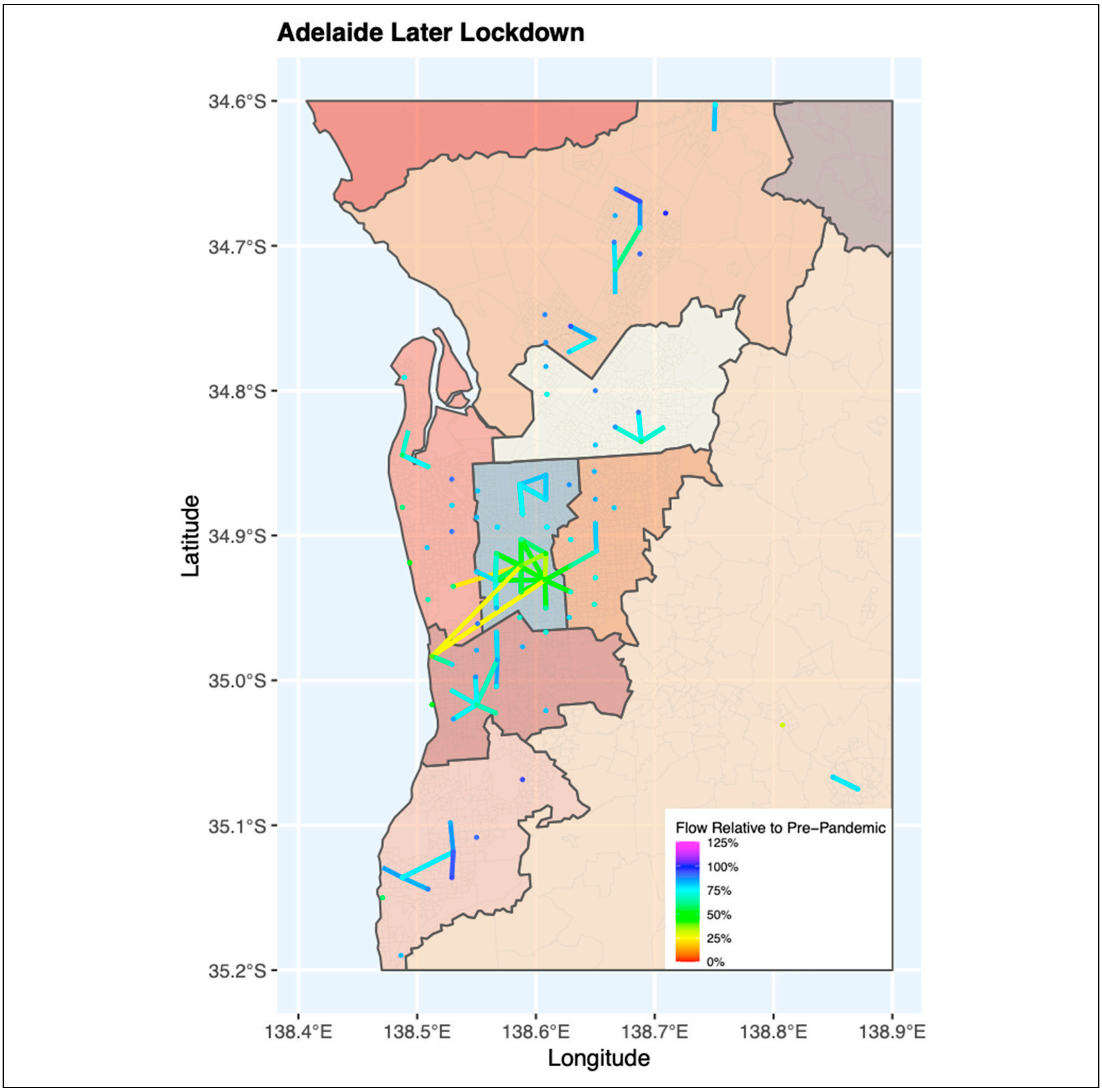
Adelaide Later Lockdown
34.6°S
34.7°S
34.8°S
34.9°S
35.0°S
35.1°S
35.2°S
Latitude
138.4°E
138.5°E
138.6°E
138.7°E
138.8°E
138.9°E
Longitude
Flow Relative to Pre–Pandemic
125%
100%
75%
50%
25%
0%

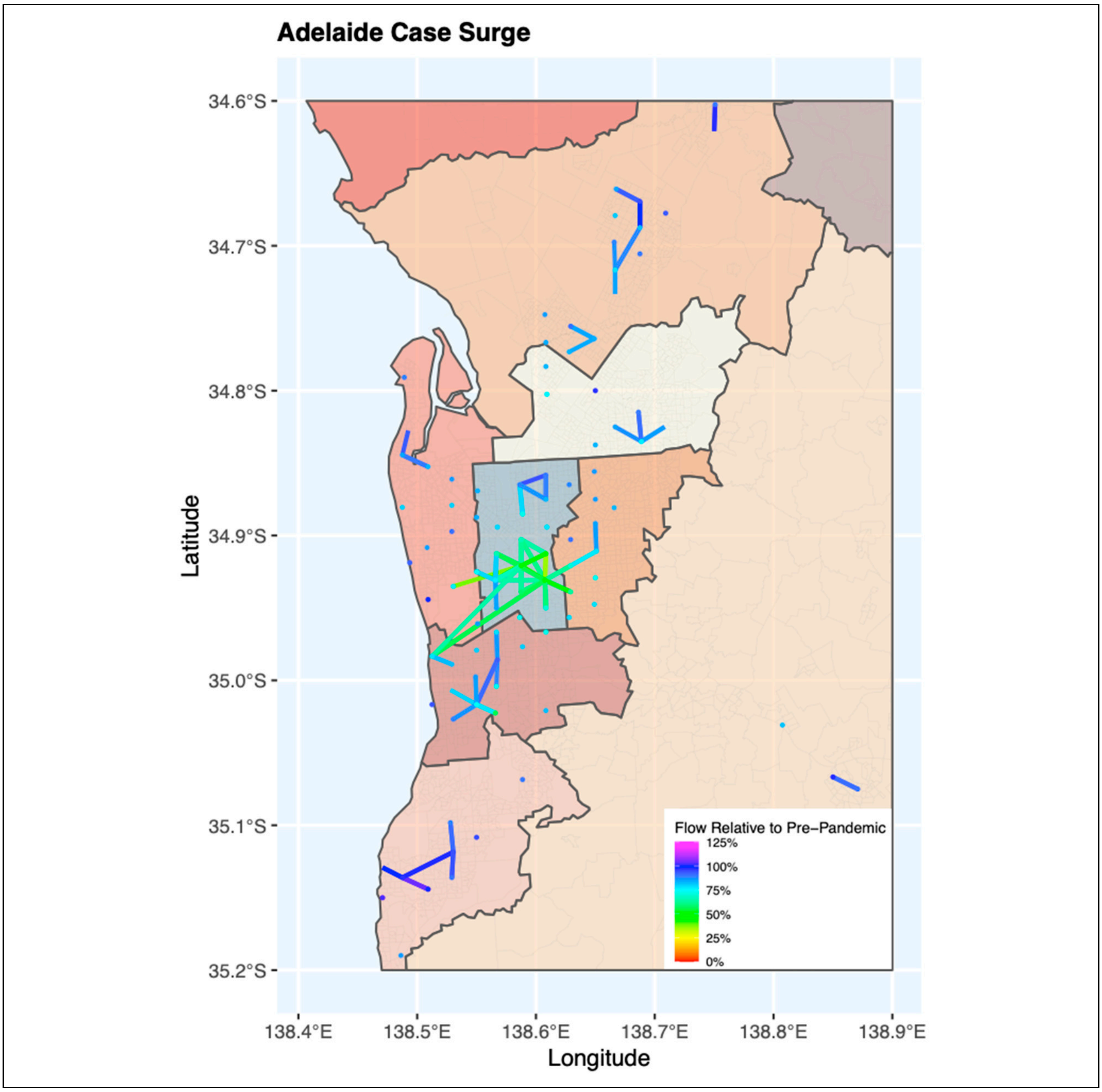
Adelaide Case Surge
34.6°S
34.7°S
34.8°S
34.9°S
35.0°S
35.1°S
35.2°S
Latitude
138.4°E
138.5°E
138.6°E
138.7°E
138.8°E
138.9°E
Longitude
Flow Relative to Pre–Pandemic
125%
100%
75%
50%
25%
0%

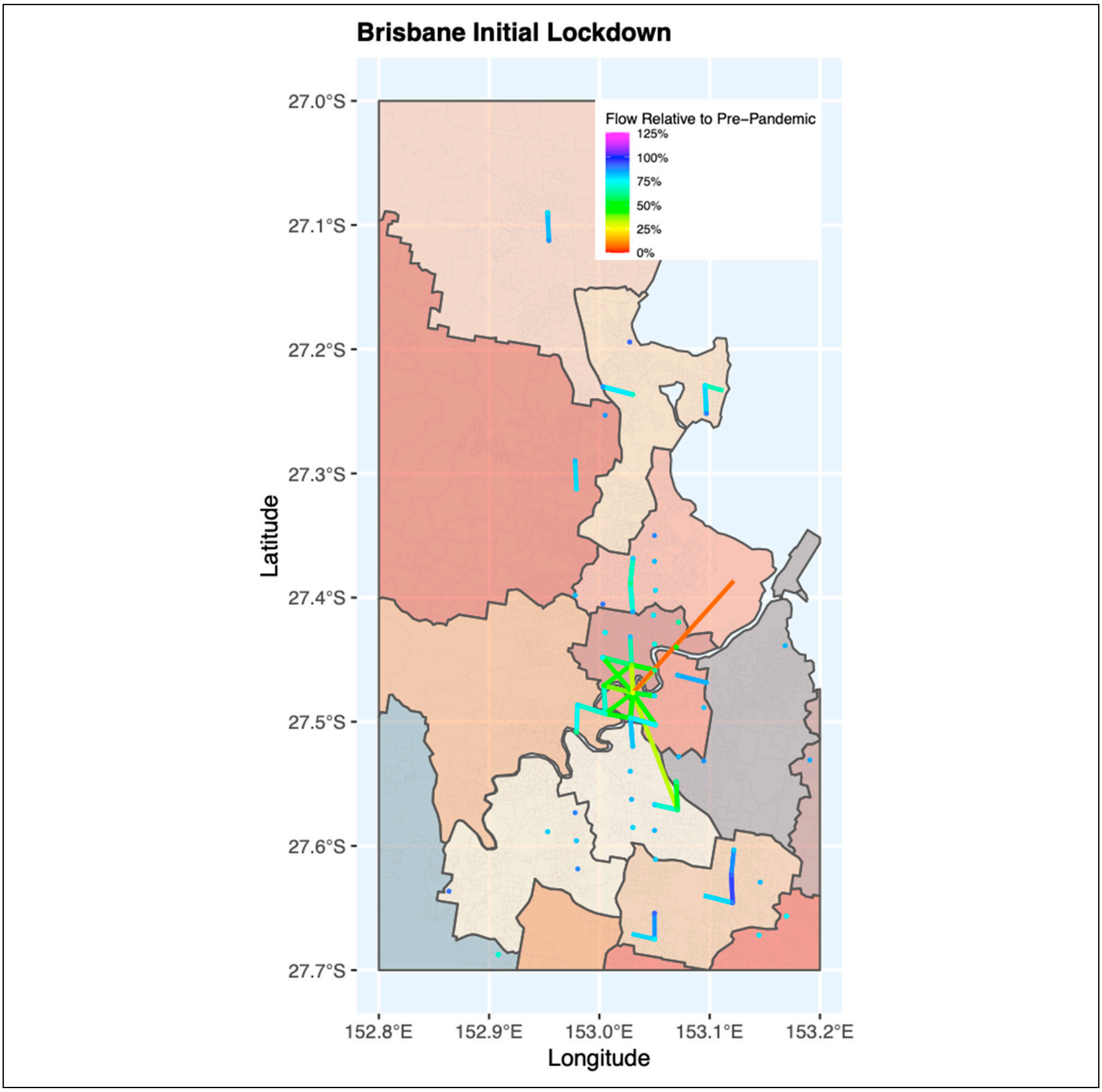
Brisbane Initial Lockdown
Flow Relative to Pre–Pandemic
125%
100%
75%
50%
25%
0%
27.0°S
27.1°S
27.2°S
27.3°S
27.4°S
27.5°S
27.6°S
27.7°S
Latitude
152.8°E
152.9°E
153.0°E
153.1°E
153.2°E
Longitude

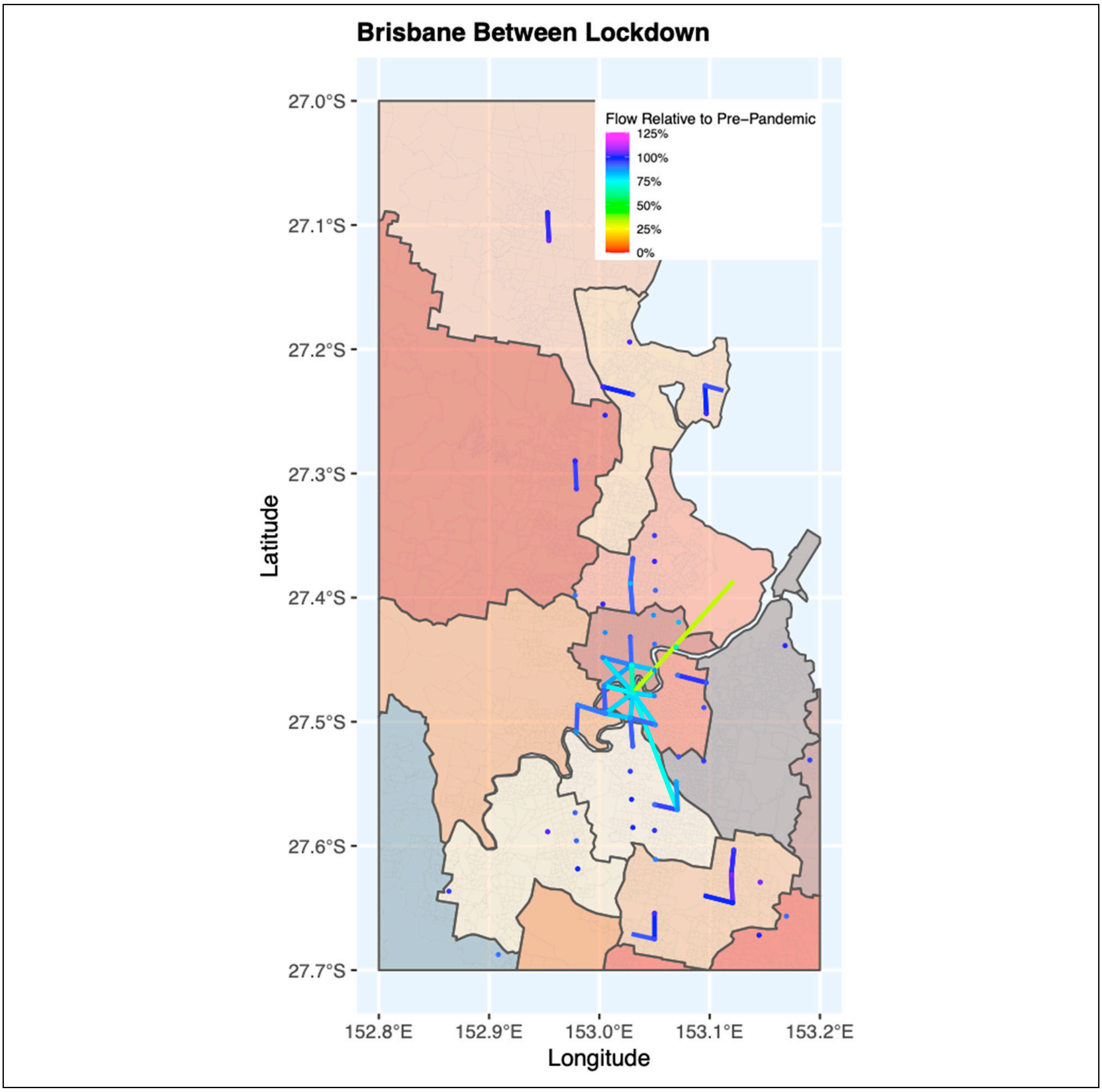
Brisbane Between Lockdown
Flow Relative to Pre−Pandemic
125%
100%
75%
50%
25%
0%
27.0°S
27.1°S
27.2°S
27.3°S
27.4°S
27.5°S
27.6°S
27.7°S
Latitude
152.8°E
152.9°E
153.0°E
153.1°E
153.2°E
Longitude

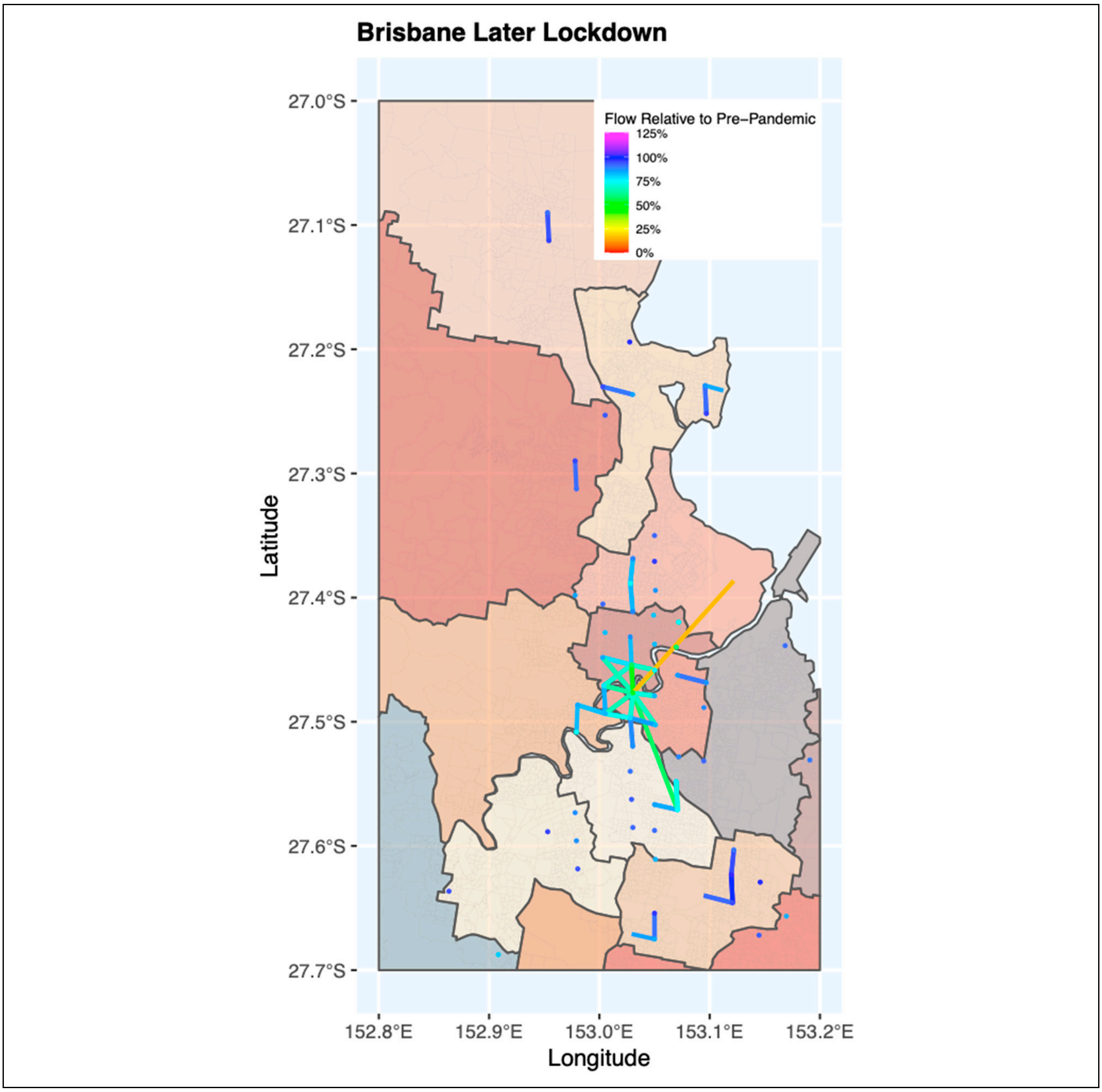
Brisbane Later Lockdown
Flow Relative to Pre−Pandemic
125%
100%
75%
50%
25%
0%
27.0°S
27.1°S
27.2°S
27.3°S
27.4°S
27.5°S
27.6°S
27.7°S
Latitude
152.8°E
152.9°E
153.0°E
153.1°E
153.2°E
Longitude

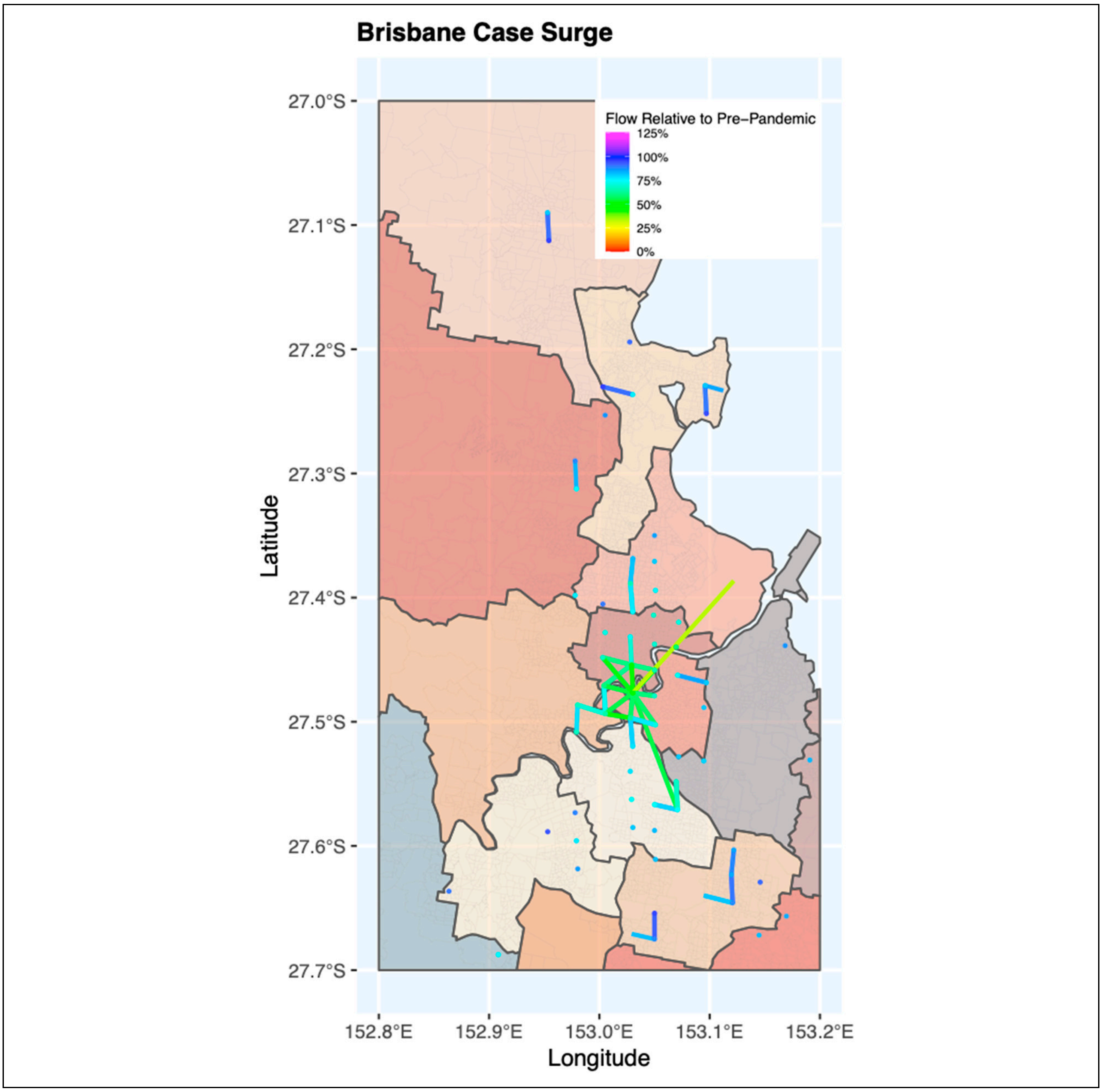
Brisbane Case Surge
Flow Relative to Pre–Pandemic
125%
100%
75%
50%
25%
0%
27.0°S
27.1°S
27.2°S
27.3°S
27.4°S
27.5°S
27.6°S
27.7°S
Latitude
152.8°E
152.9°E
153.0°E
153.1°E
153.2°E
Longitude

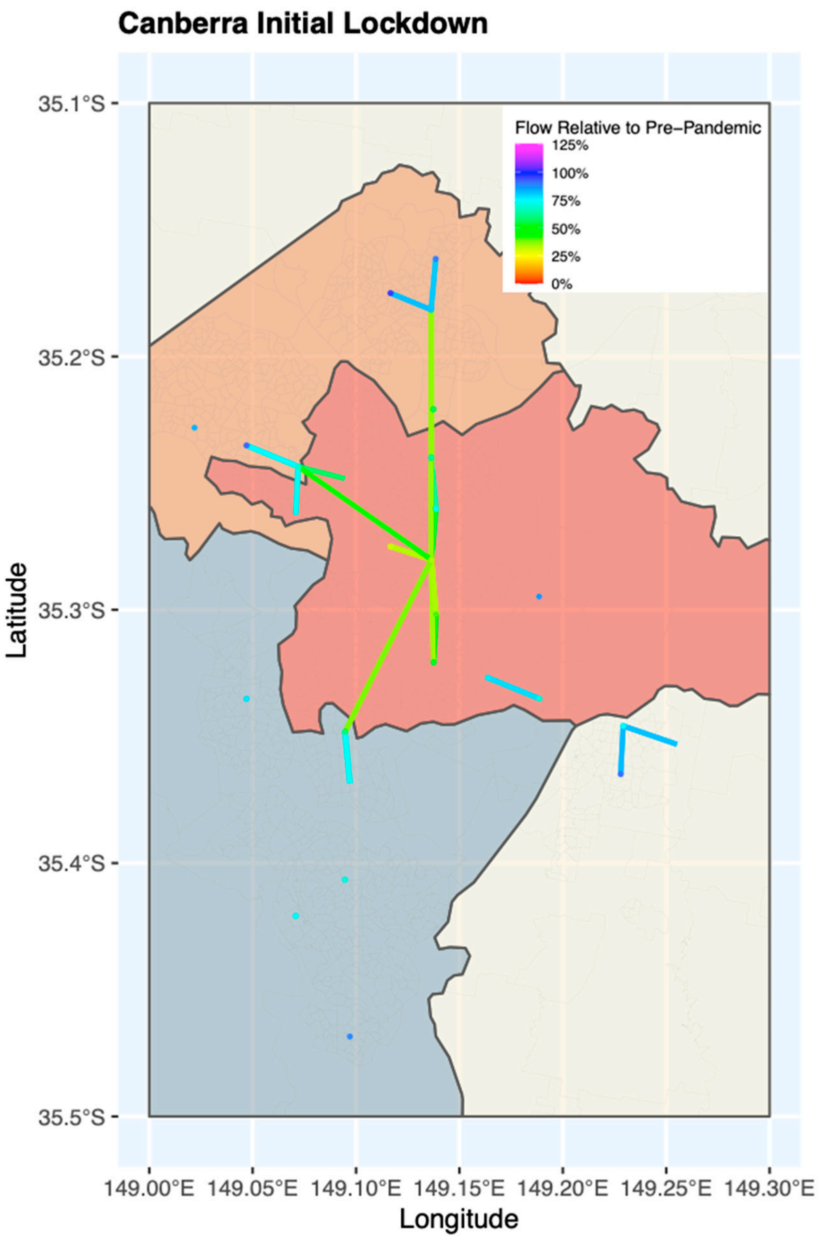
Canberra Initial Lockdown
Flow Relative to Pre−Pandemic
125%
100%
75%
50%
25%
0%
35.1°S
35.2°S
35.3°S
35.4°S
35.5°S
Latitude
149.00°E
149.05°E
149.10°E
149.15°E
149.20°E
149.25°E
149.30°E
Longitude

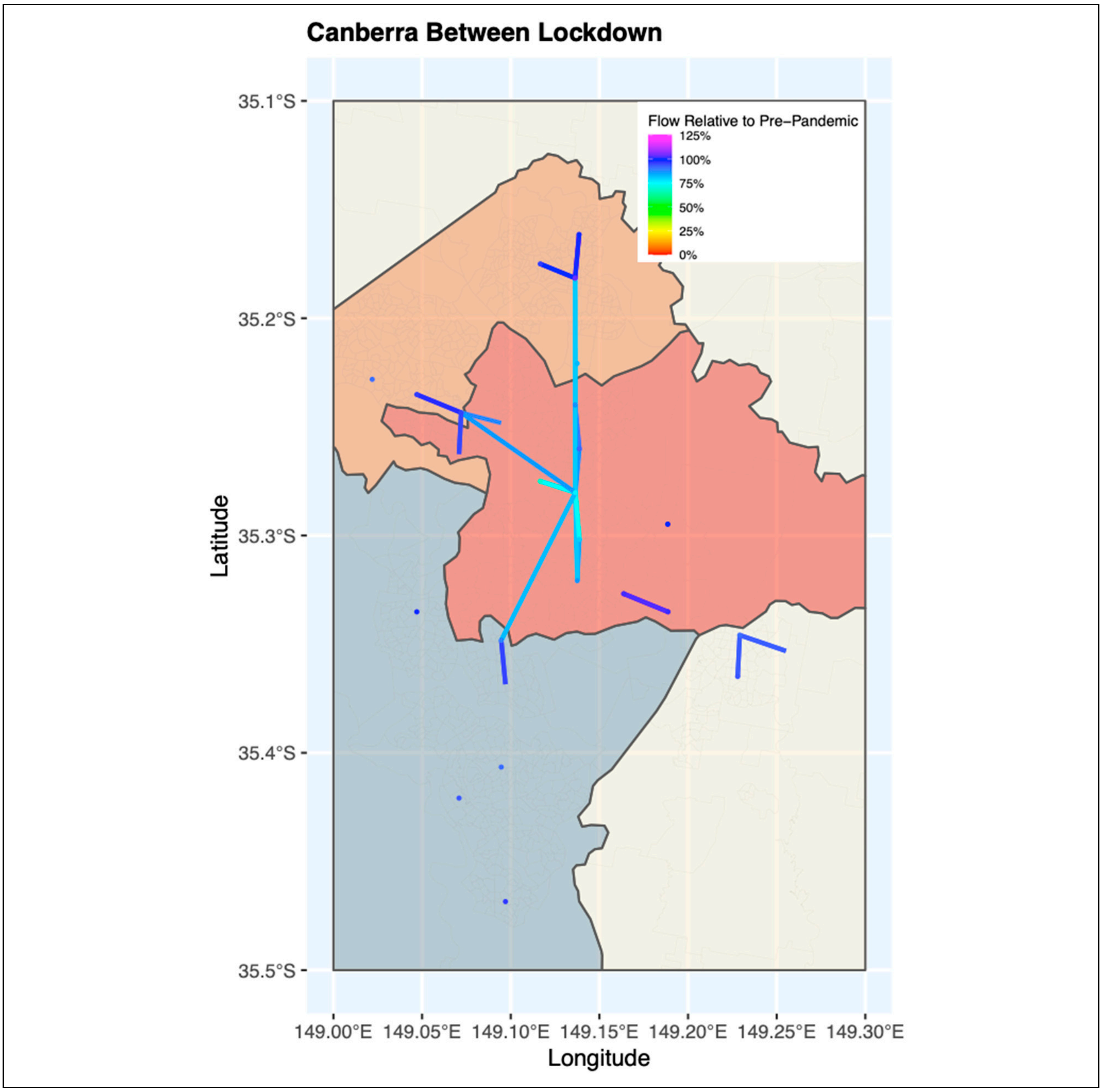
Canberra Between Lockdown
Flow Relative to Pre−Pandemic
125%
100%
75%
50%
25%
0%
35.1°S
35.2°S
35.3°S
35.4°S
35.5°S
Latitude
149.00°E 149.05°E 149.10°E 149.15°E 149.20°E 149.25°E 149.30°E
Longitude

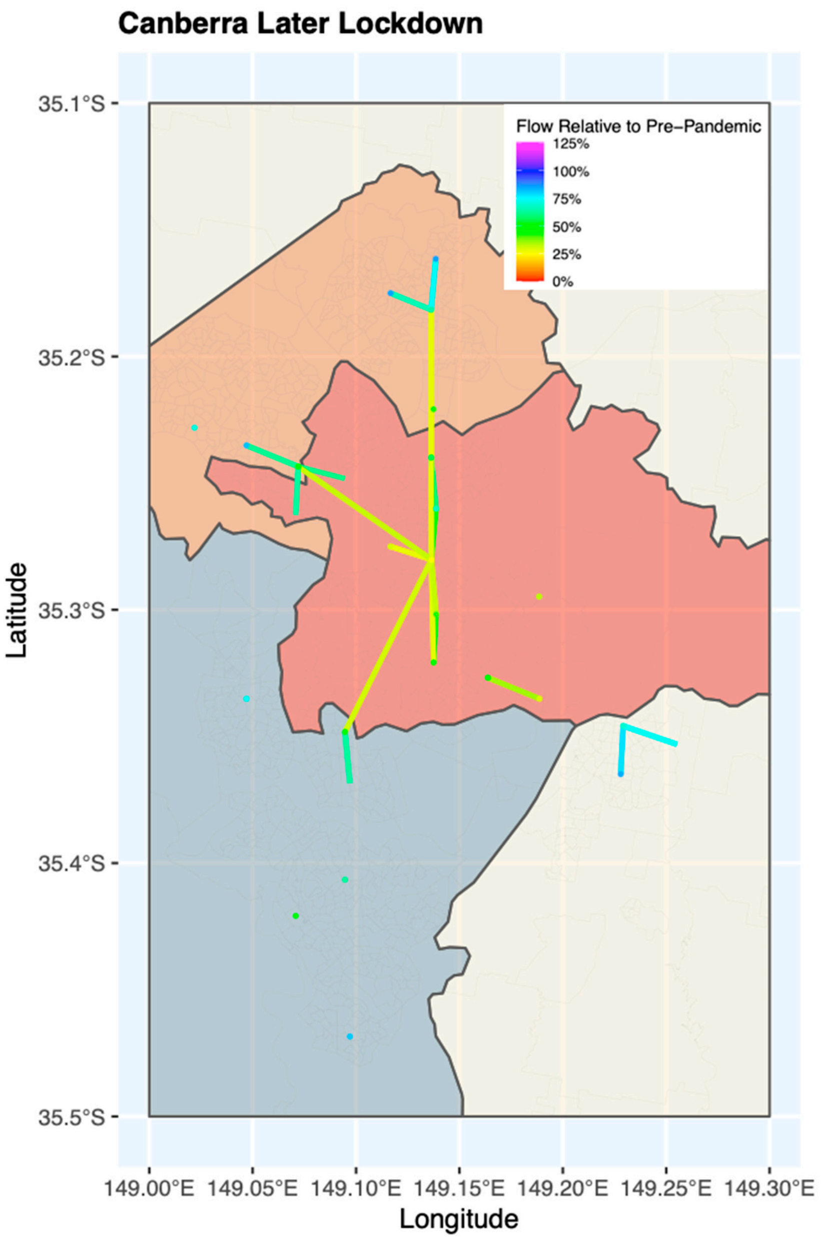
Canberra Later Lockdown
Flow Relative to Pre−Pandemic
125%
100%
75%
50%
25%
0%
35.1°S
35.2°S
35.3°S
35.4°S
35.5°S
Latitude
149.00°E
149.05°E
149.10°E
149.15°E
149.20°E
149.25°E
149.30°E
Longitude

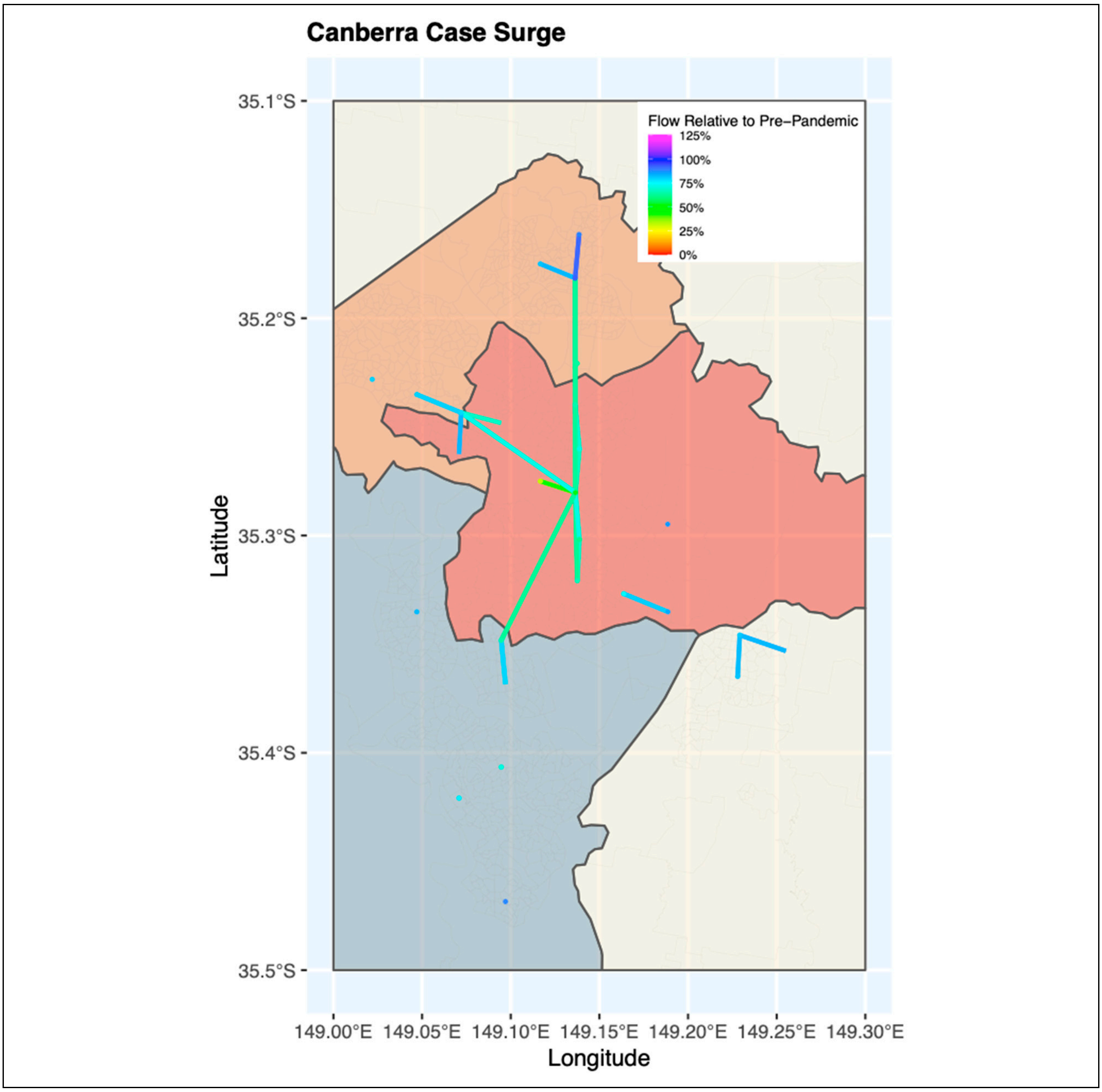
Canberra Case Surge
Flow Relative to Pre–Pandemic
125%
100%
75%
50%
25%
0%
35.1°S
35.2°S
35.3°S
35.4°S
35.5°S
Latitude
149.00°E
149.05°E
149.10°E
149.15°E
149.20°E
149.25°E
149.30°E
Longitude

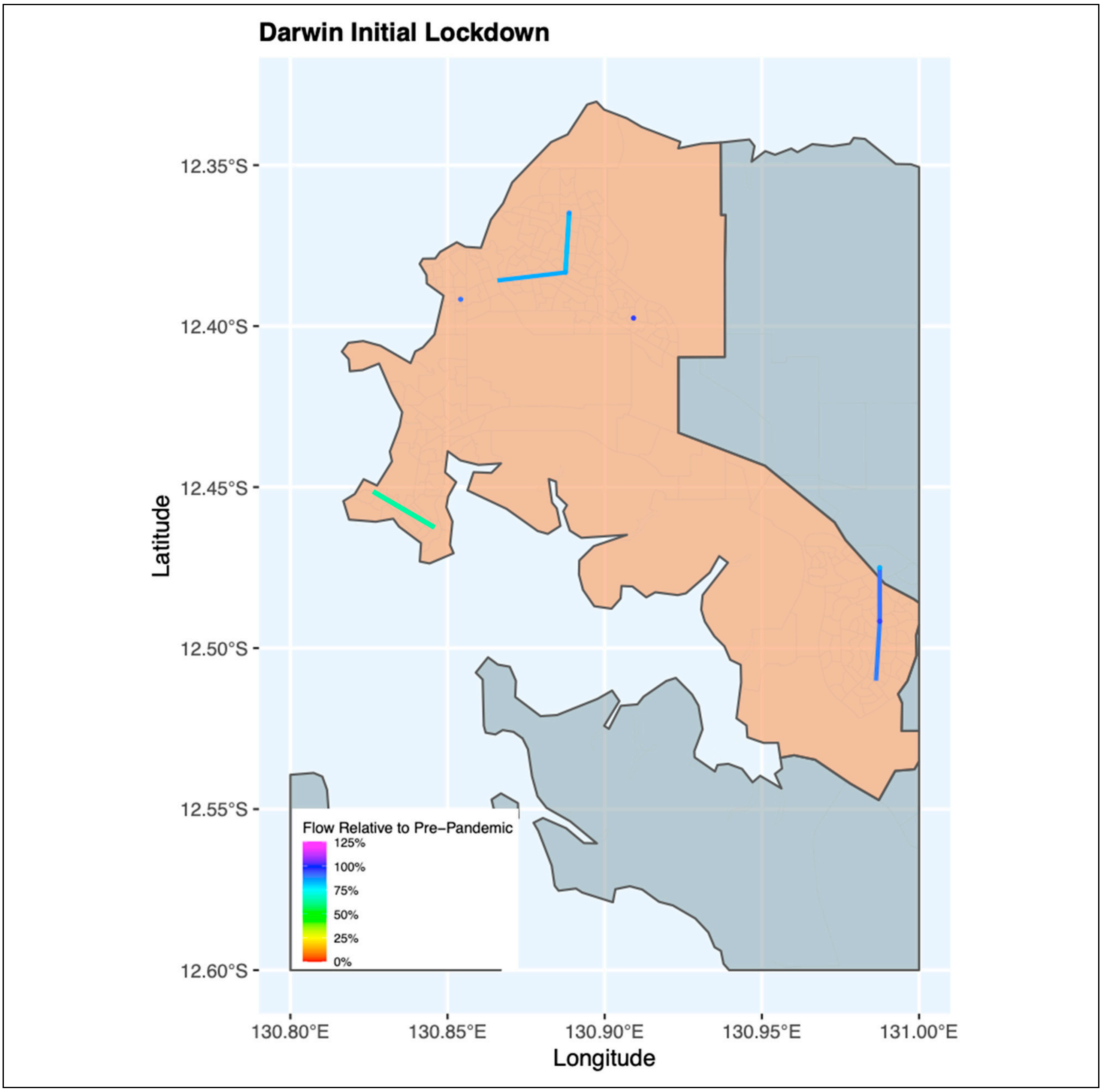
Darwin Initial Lockdown
12.35°S
12.40°S
12.45°S
12.50°S
12.55°S
12.60°S
Latitude
130.80°E
130.85°E
130.90°E
130.95°E
131.00°E
Longitude
Flow Relative to Pre−Pandemic
125%
100%
75%
50%
25%
0%

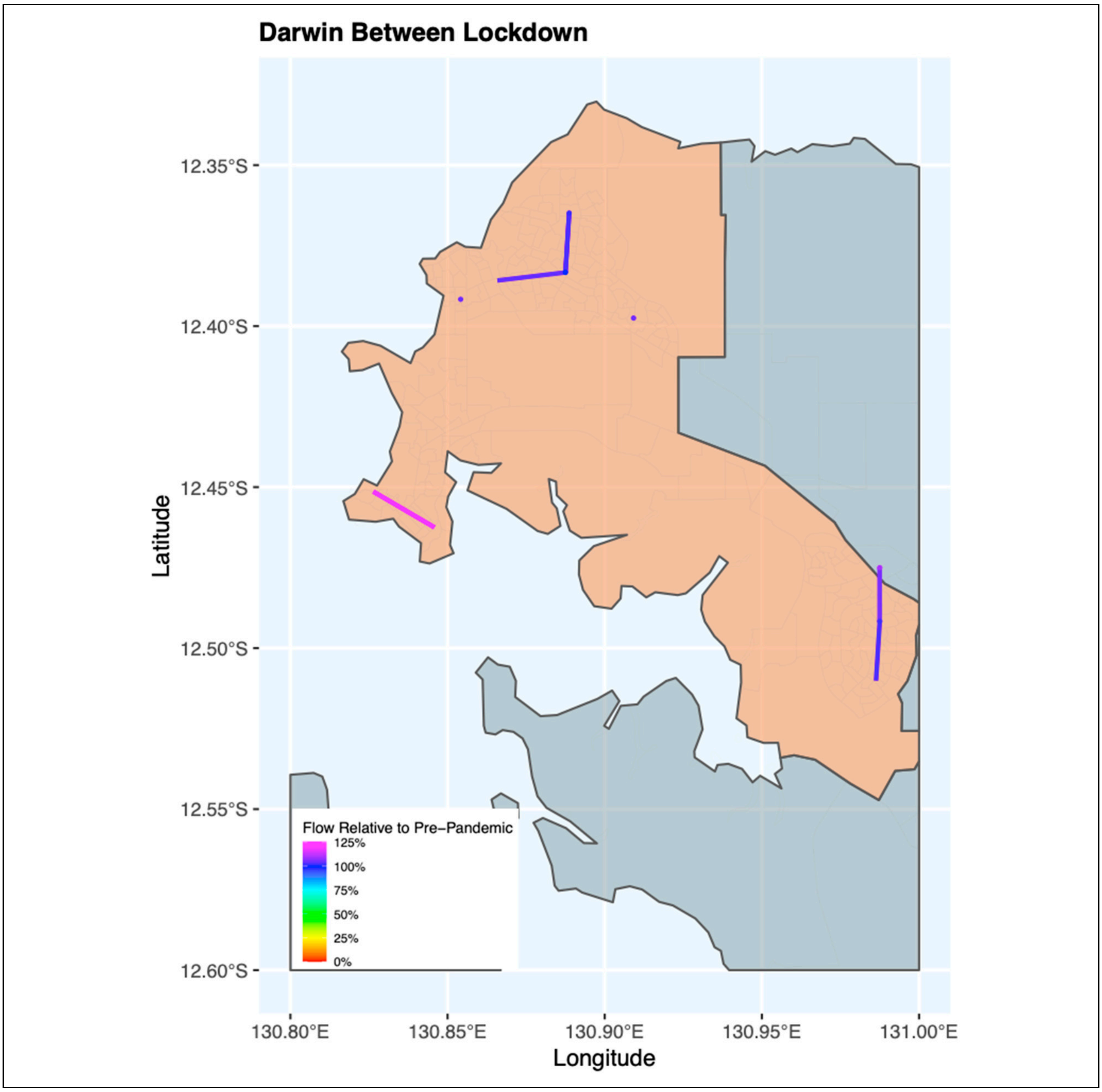
Darwin Between Lockdown
12.35°S
12.40°S
12.45°S
12.50°S
12.55°S
12.60°S
Latitude
130.80°E
130.85°E
130.90°E
130.95°E
131.00°E
Longitude
Flow Relative to Pre−Pandemic
125%
100%
75%
50%
25%
0%

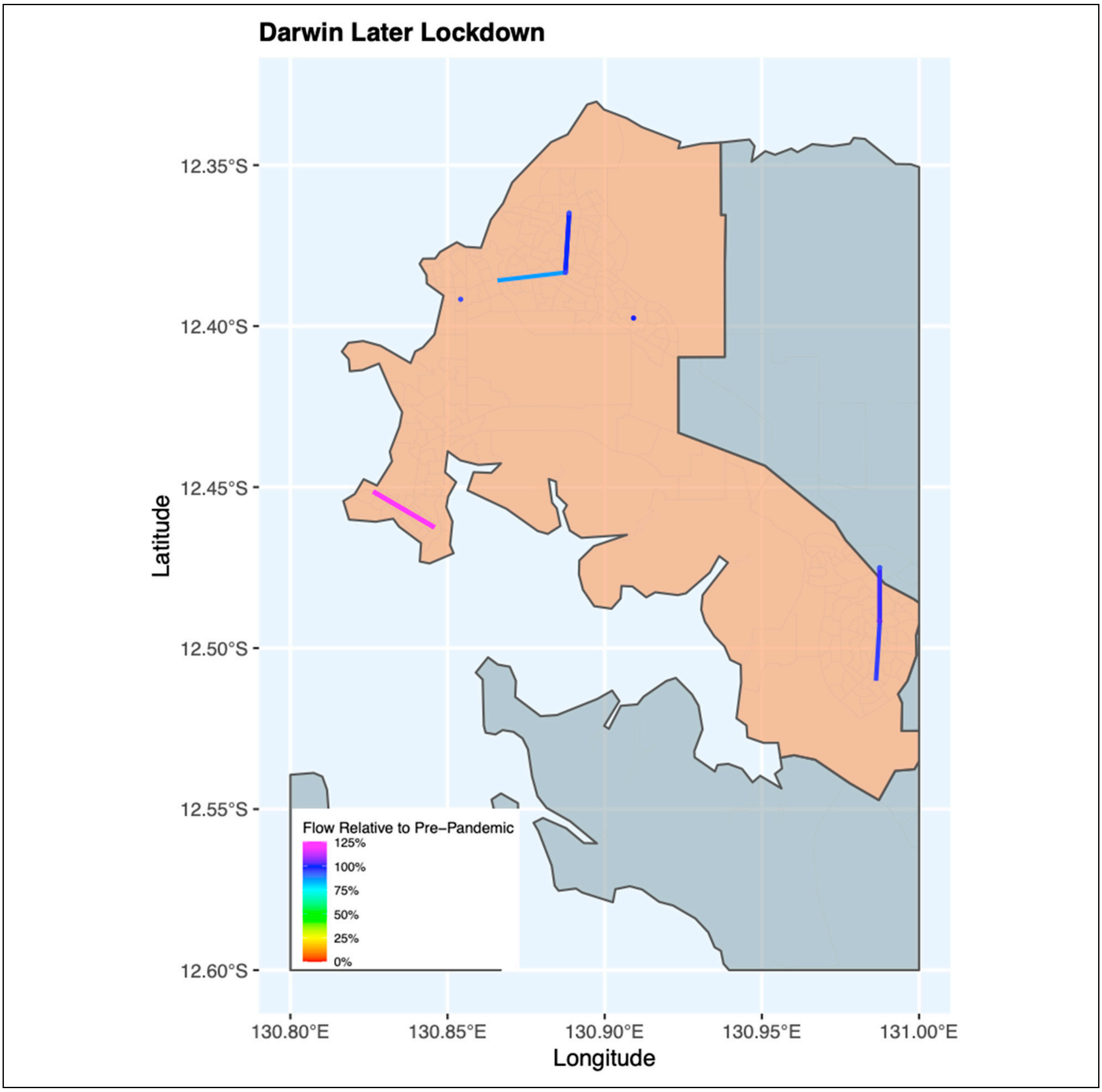
Darwin Later Lockdown
12.35°S
12.40°S
12.45°S
12.50°S
12.55°S
12.60°S
Latitude
130.80°E
130.85°E
130.90°E
130.95°E
131.00°E
Longitude
Flow Relative to Pre−Pandemic
125%
100%
75%
50%
25%
0%

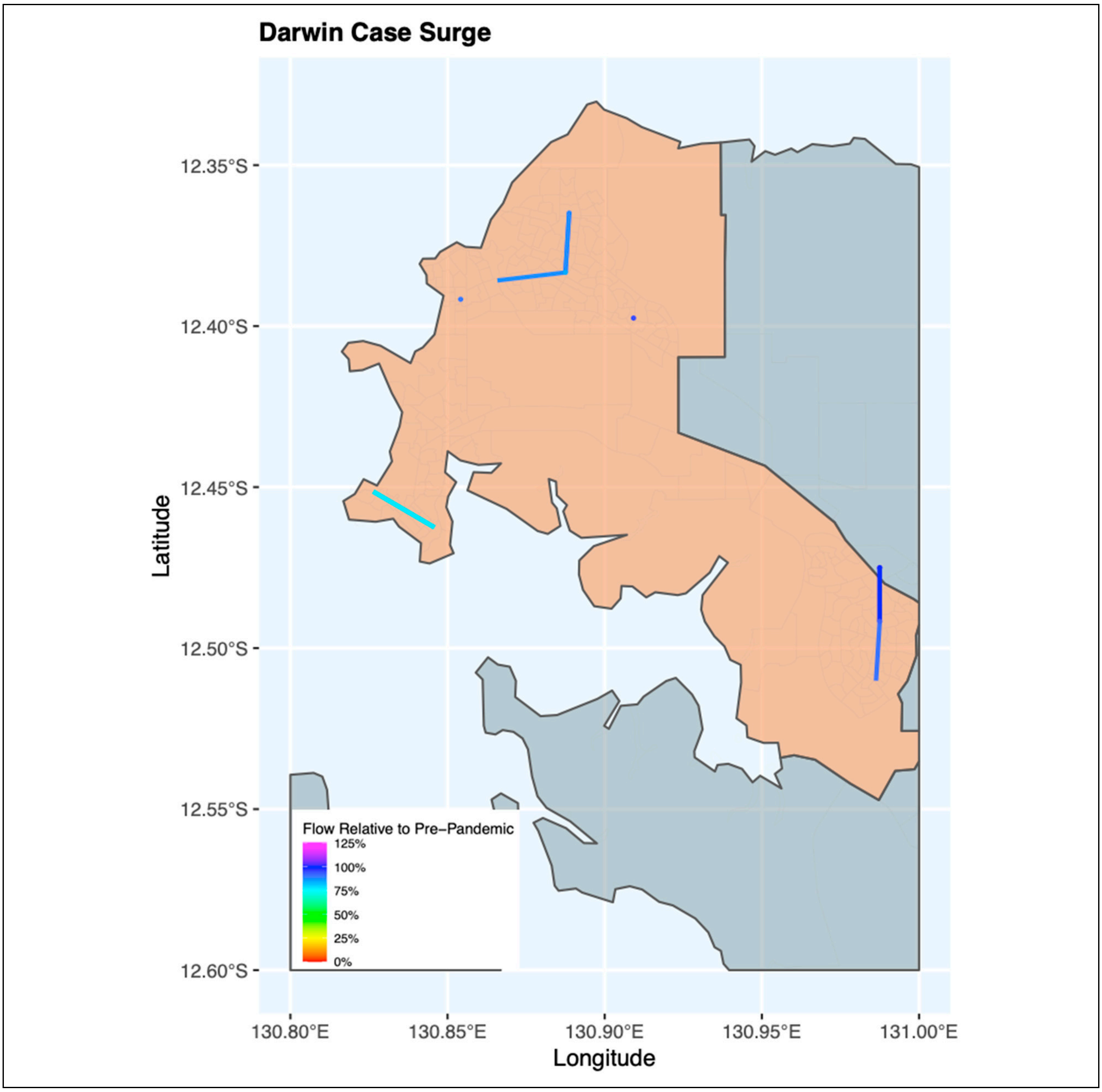
Darwin Case Surge
12.35°S
12.40°S
12.45°S
12.50°S
12.55°S
12.60°S
Latitude
130.80°E
130.85°E
130.90°E
130.95°E
131.00°E
Longitude
Flow Relative to Pre−Pandemic
125%
100%
75%
50%
25%
0%

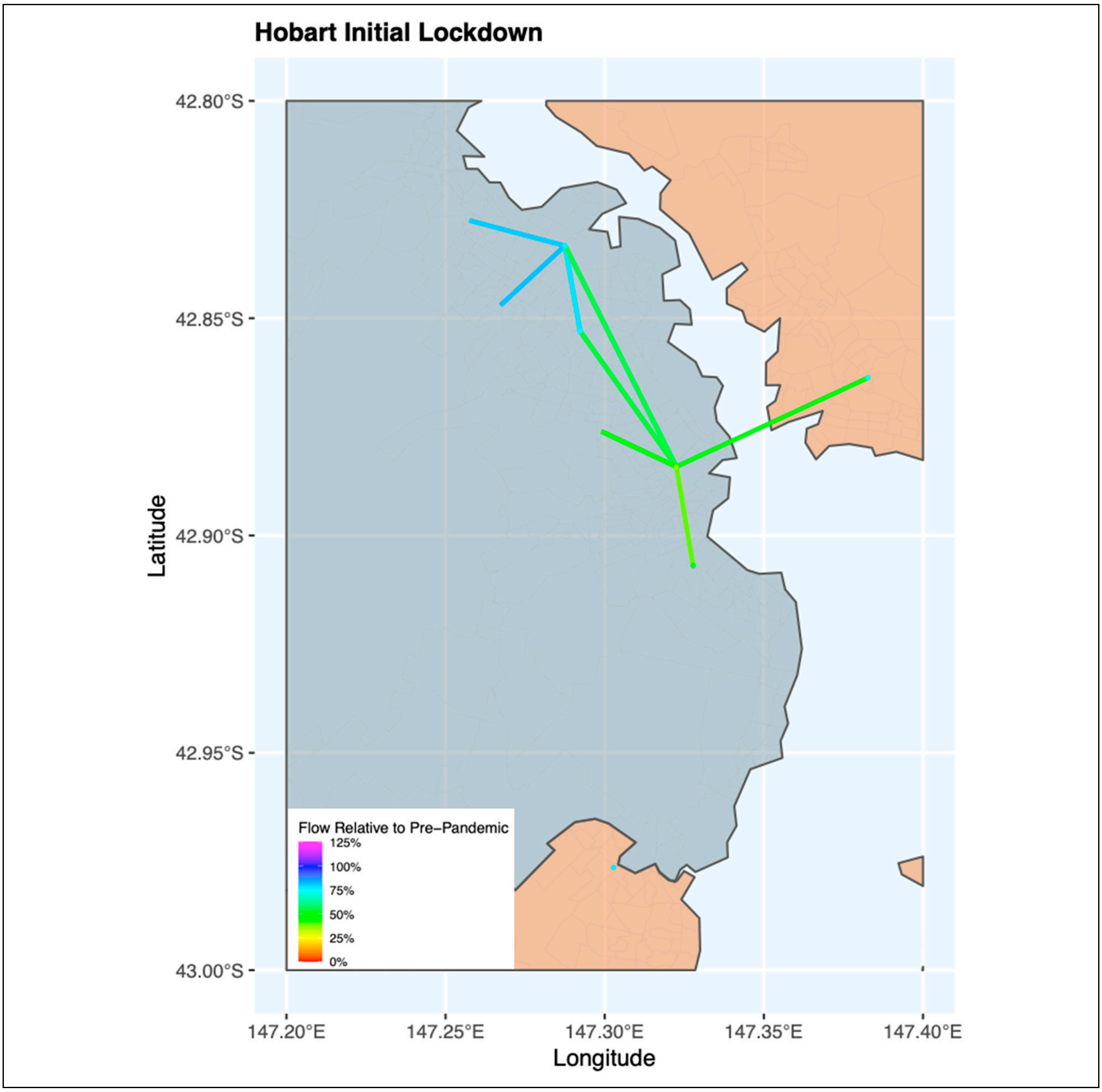
Hobart Initial Lockdown
42.80°S
42.85°S
42.90°S
42.95°S
43.00°S
Latitude
147.20°E
147.25°E
147.30°E
147.35°E
147.40°E
Longitude
Flow Relative to Pre–Pandemic
125%
100%
75%
50%
25%
0%

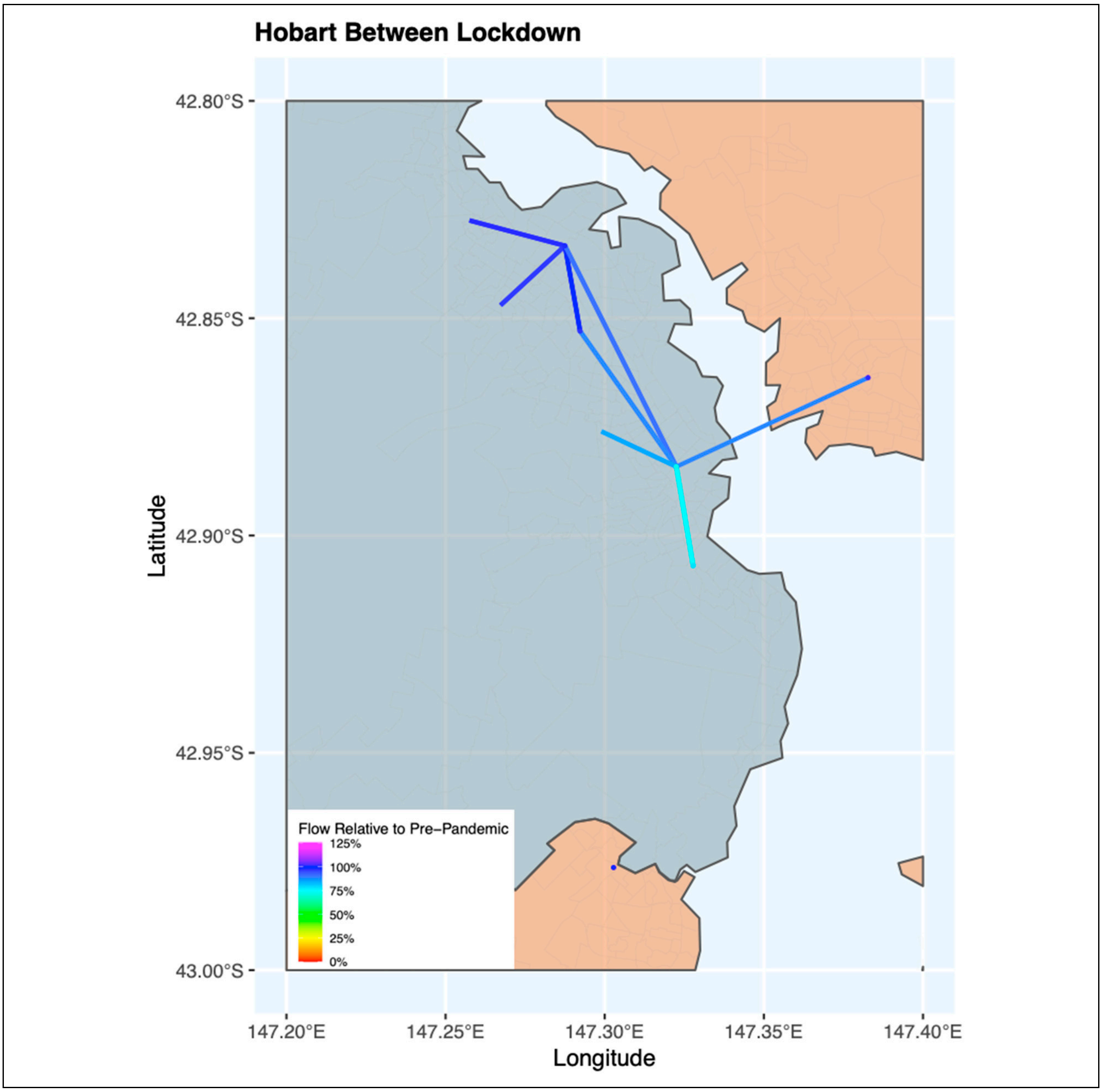
Hobart Between Lockdown
Latitude
42.80°S
42.85°S
42.90°S
42.95°S
43.00°S
147.20°E
147.25°E
147.30°E
147.35°E
147.40°E
Longitude
Flow Relative to Pre–Pandemic
125%
100%
75%
50%
25%
0%

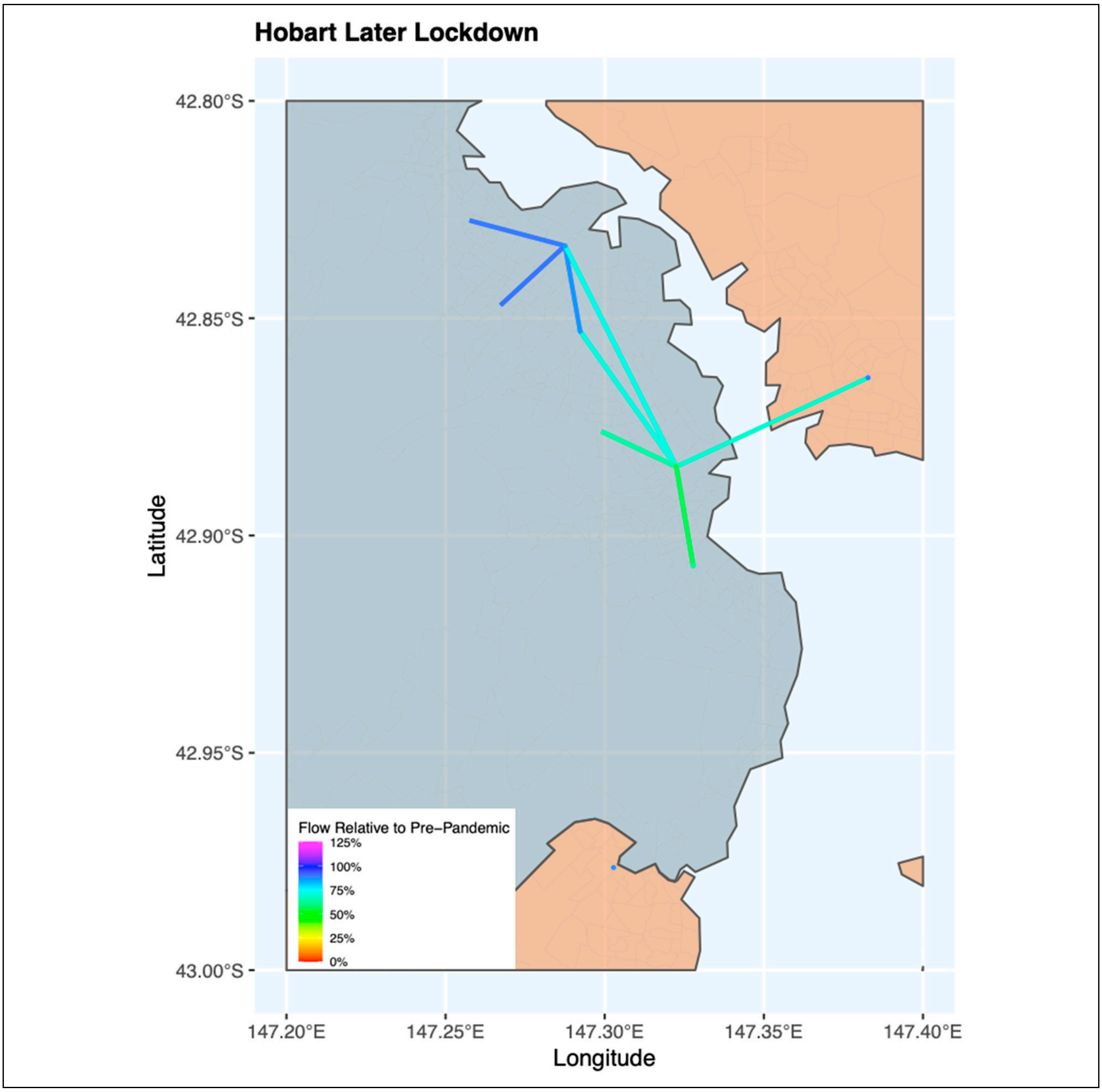
Hobart Later Lockdown
42.80°S
42.85°S
42.90°S
42.95°S
43.00°S
Latitude
147.20°E
147.25°E
147.30°E
147.35°E
147.40°E
Longitude
Flow Relative to Pre–Pandemic
125%
100%
75%
50%
25%
0%

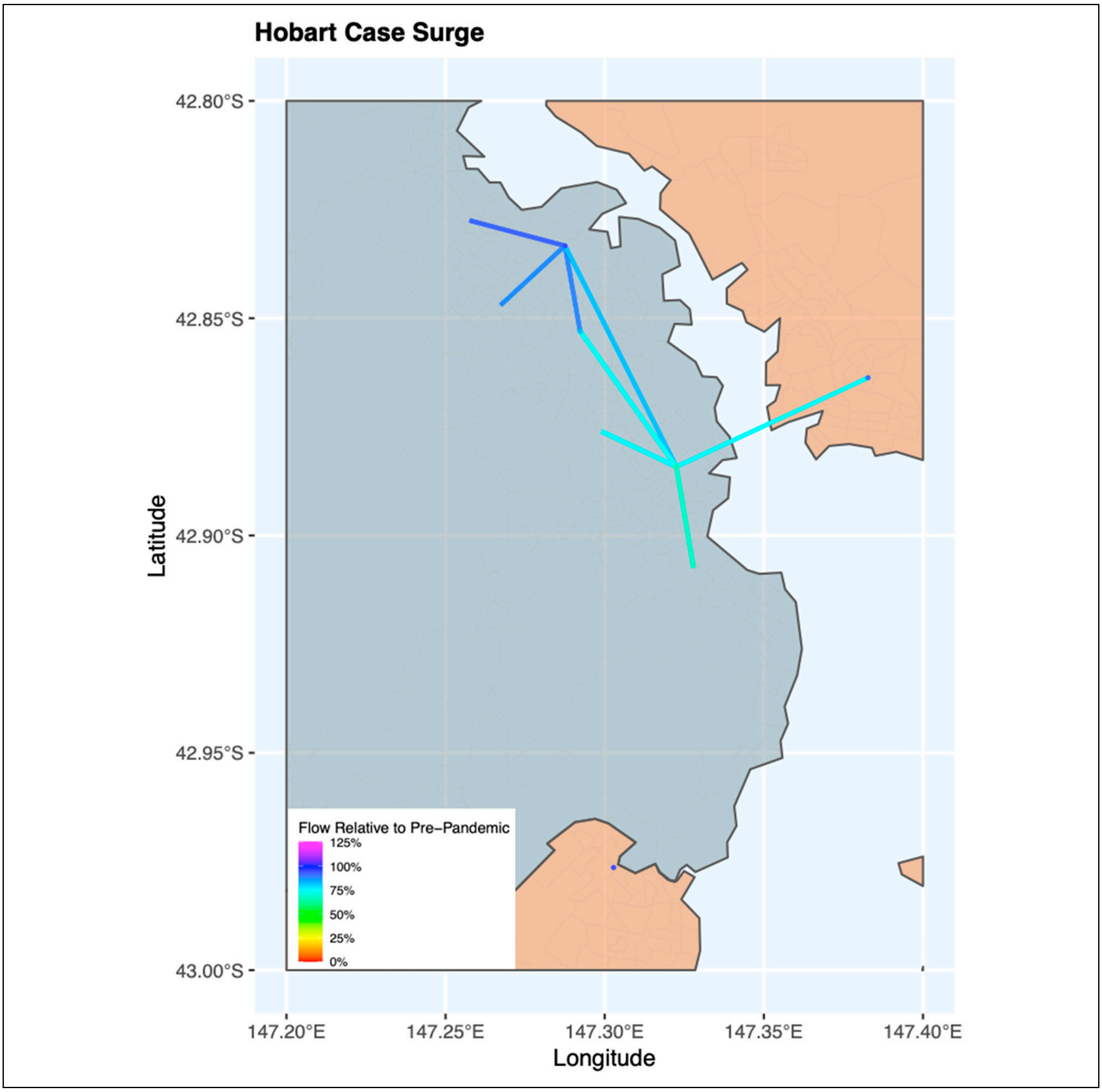
Hobart Case Surge
42.80°S
42.85°S
42.90°S
42.95°S
43.00°S
Latitude
147.20°E
147.25°E
147.30°E
147.35°E
147.40°E
Longitude
Flow Relative to Pre–Pandemic
125%
100%
75%
50%
25%
0%

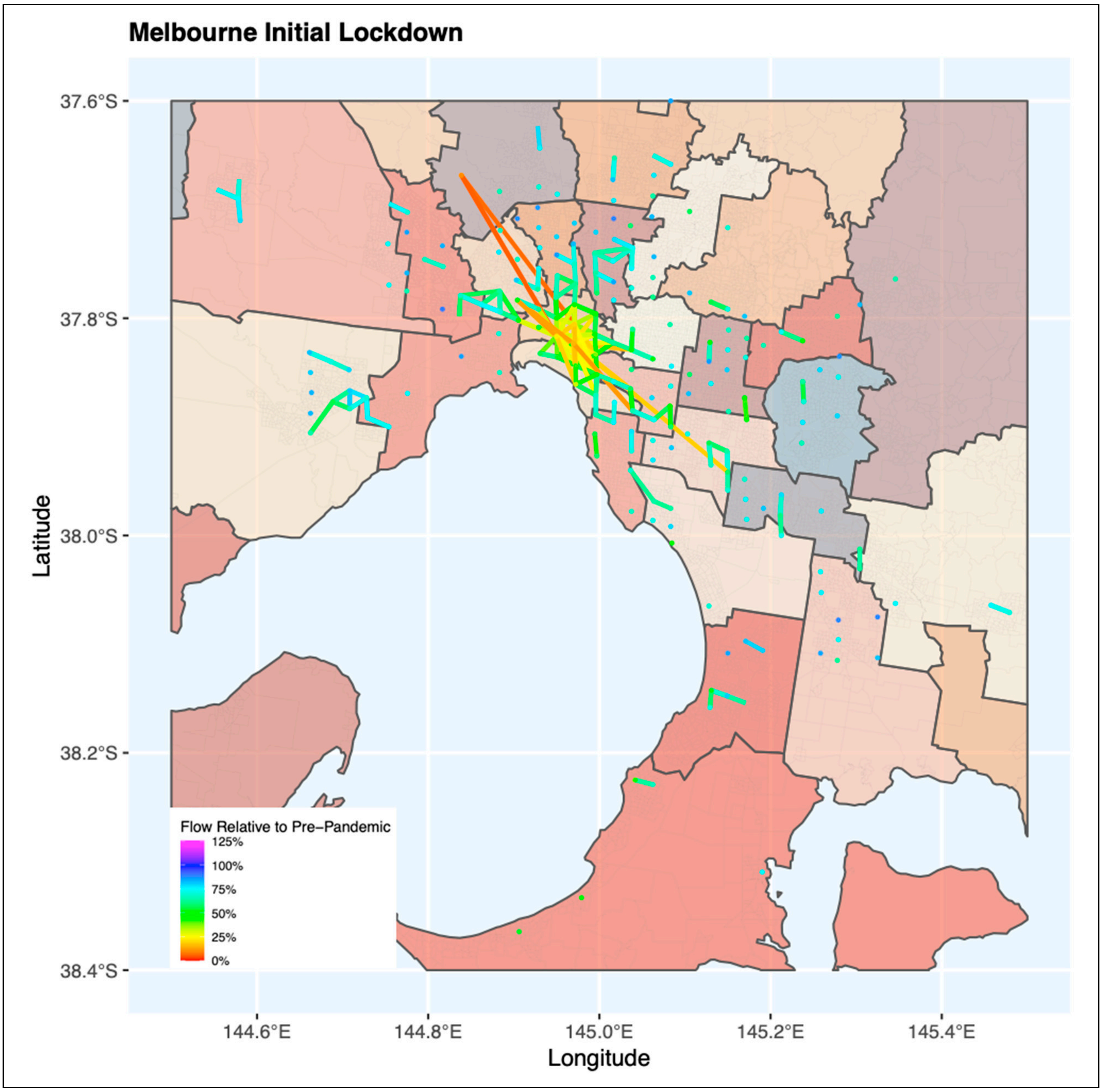
Melbourne Initial Lockdown
Latitude
37.6°S
37.8°S
38.0°S
38.2°S
38.4°S
Longitude
144.6°E
144.8°E
145.0°E
145.2°E
145.4°E
Flow Relative to Pre−Pandemic
125%
100%
75%
50%
25%
0%

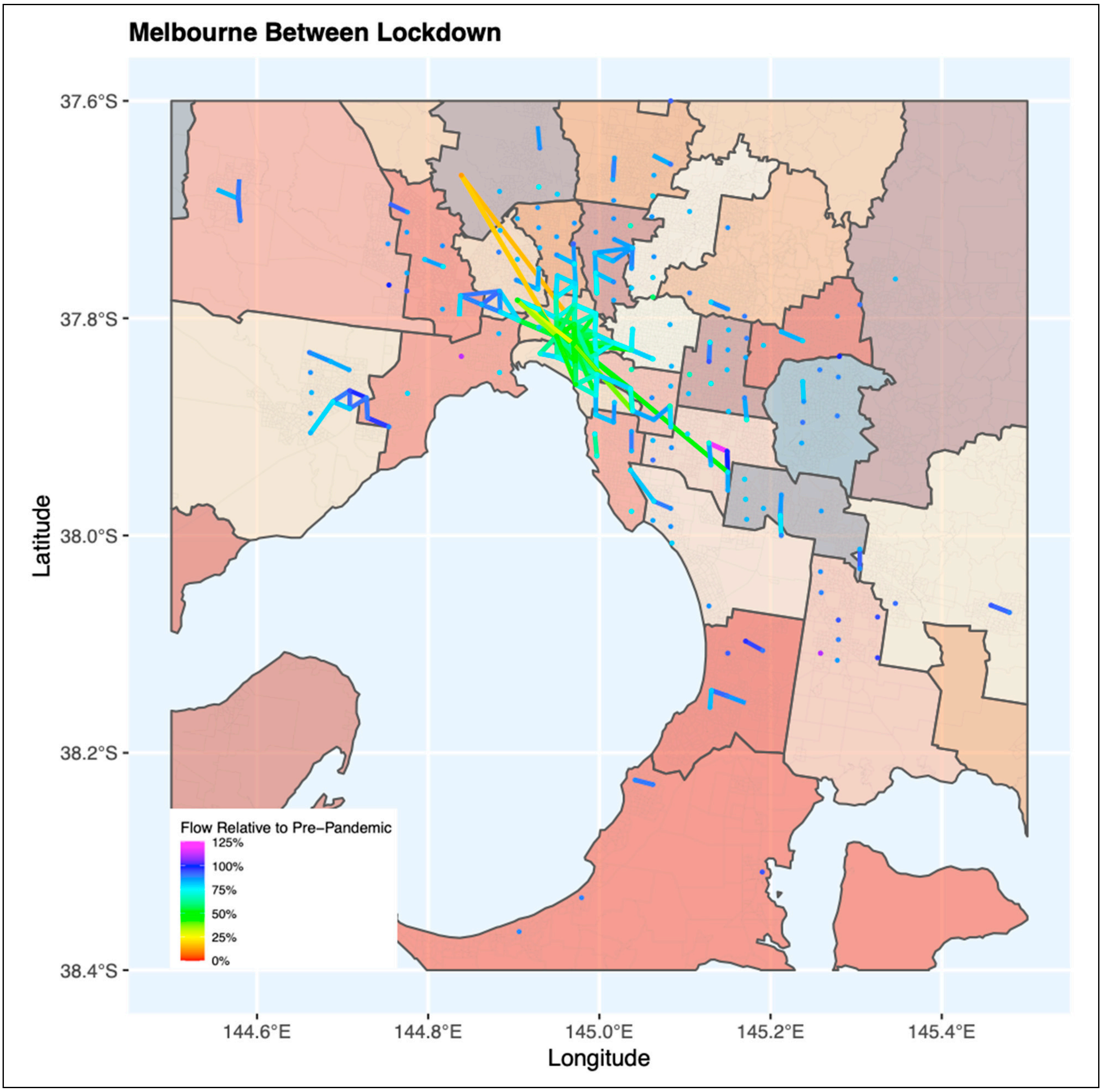

Melbourne Between Lockdown
37.6°S
37.8°S
38.0°S
38.2°S
38.4°S
Latitude
144.6°E
144.8°E
145.0°E
145.2°E
145.4°E
Longitude
Flow Relative to Pre–Pandemic
125%
100%
75%
50%
25%
0%

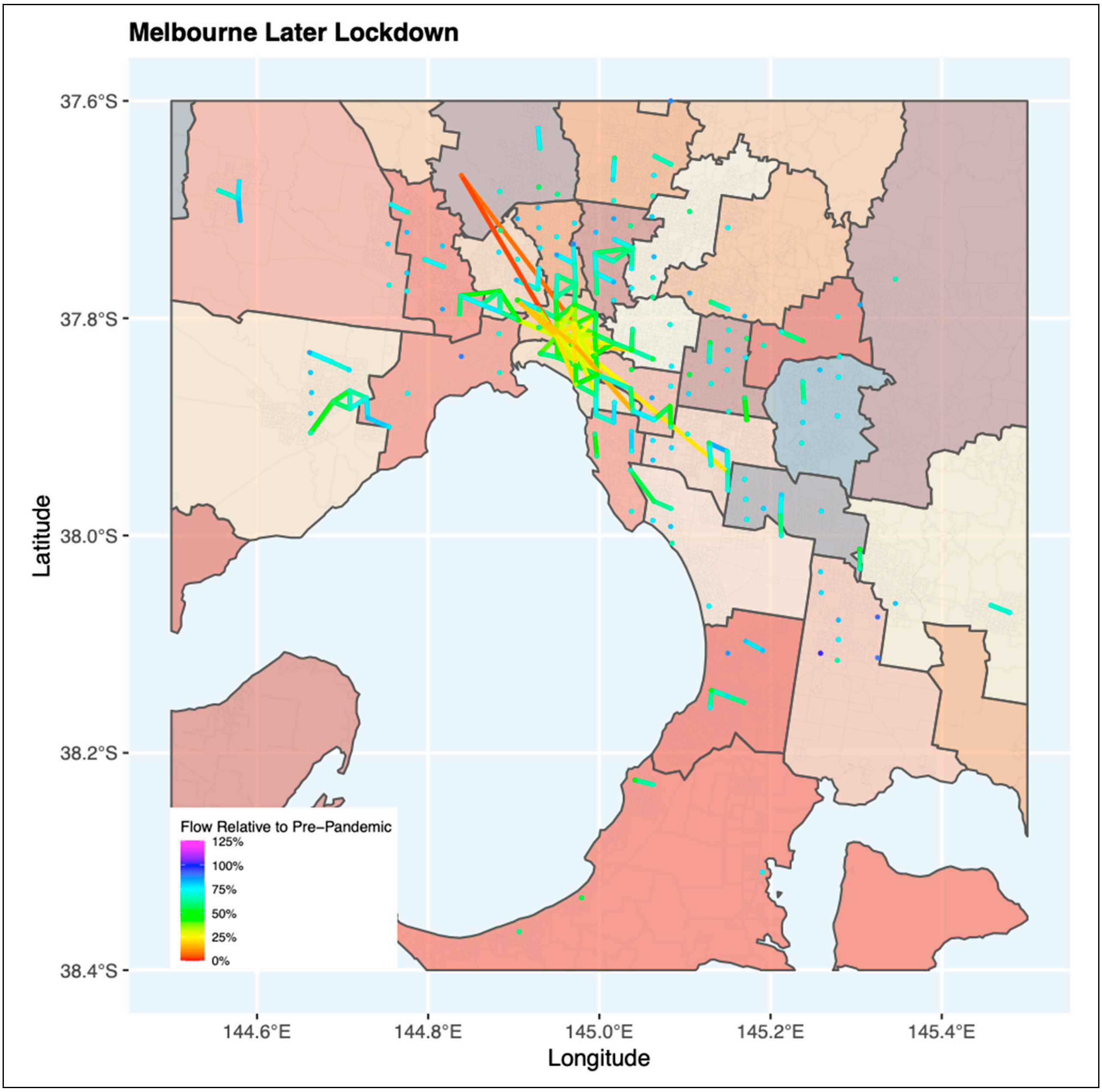
Melbourne Later Lockdown
37.6°S
37.8°S
38.0°S
38.2°S
38.4°S
Latitude
144.6°E
144.8°E
145.0°E
145.2°E
145.4°E
Longitude
Flow Relative to Pre−Pandemic
125%
100%
75%
50%
25%
0%

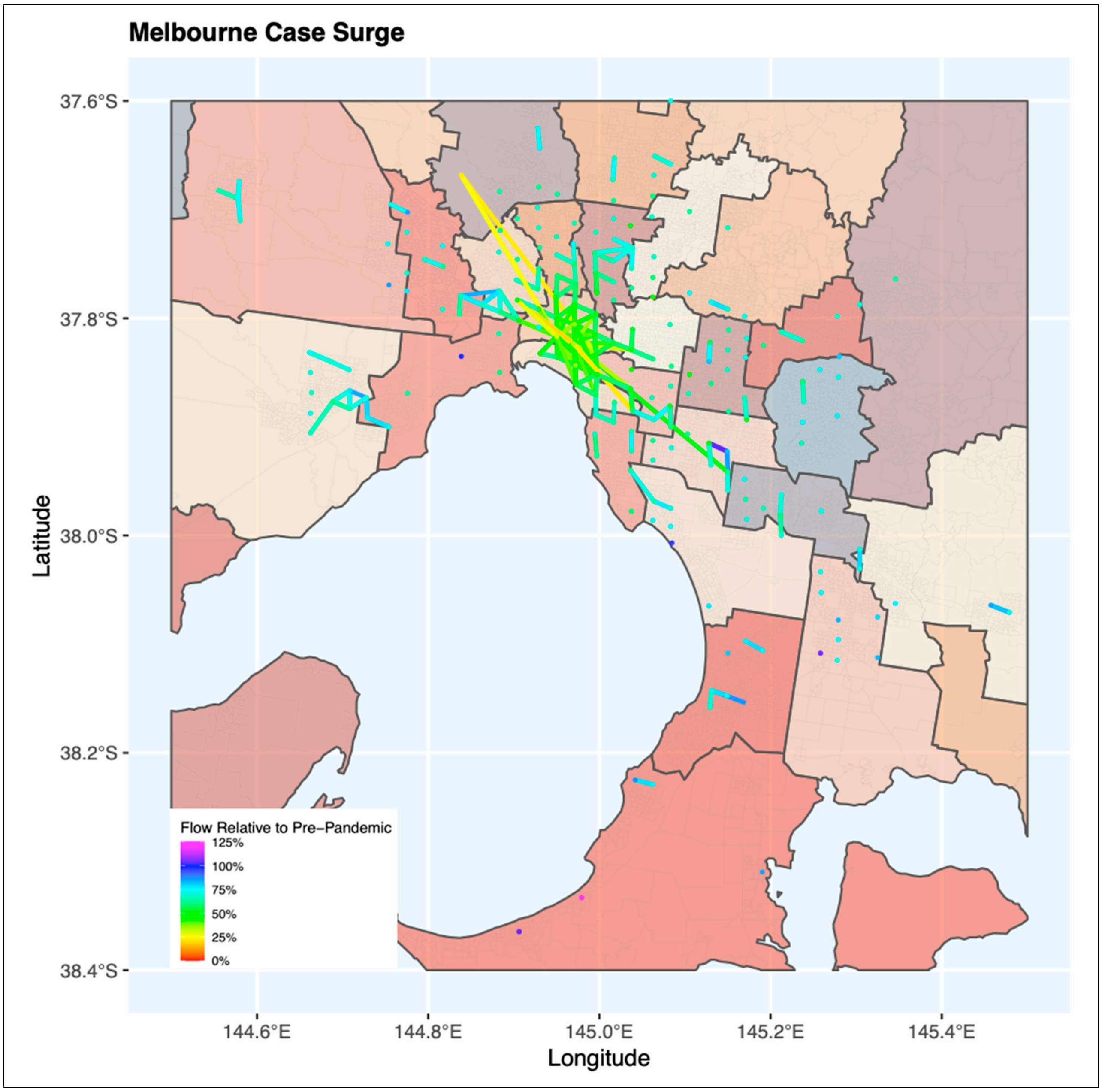
Melbourne Case Surge
37.6°S
37.8°S
38.0°S
38.2°S
38.4°S
Latitude
144.6°E
144.8°E
145.0°E
145.2°E
145.4°E
Longitude
Flow Relative to Pre-Pandemic
125%
100%
75%
50%
25%
0%

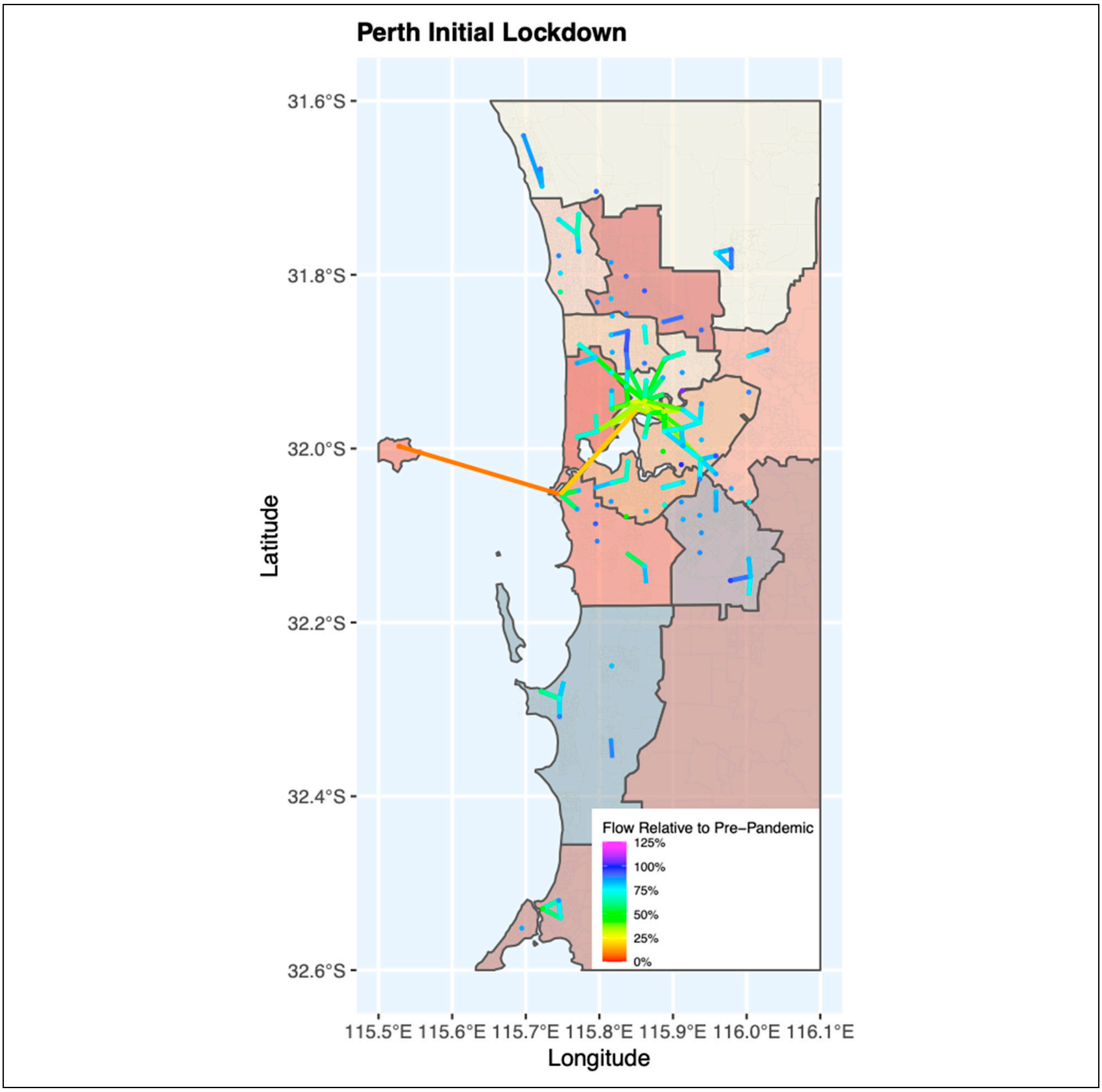
Perth Initial Lockdown
31.6°S
31.8°S
32.0°S
32.2°S
32.4°S
32.6°S
Latitude
115.5°E 115.6°E 115.7°E 115.8°E 115.9°E 116.0°E 116.1°E
Longitude
Flow Relative to Pre−Pandemic
125%
100%
75%
50%
25%
0%

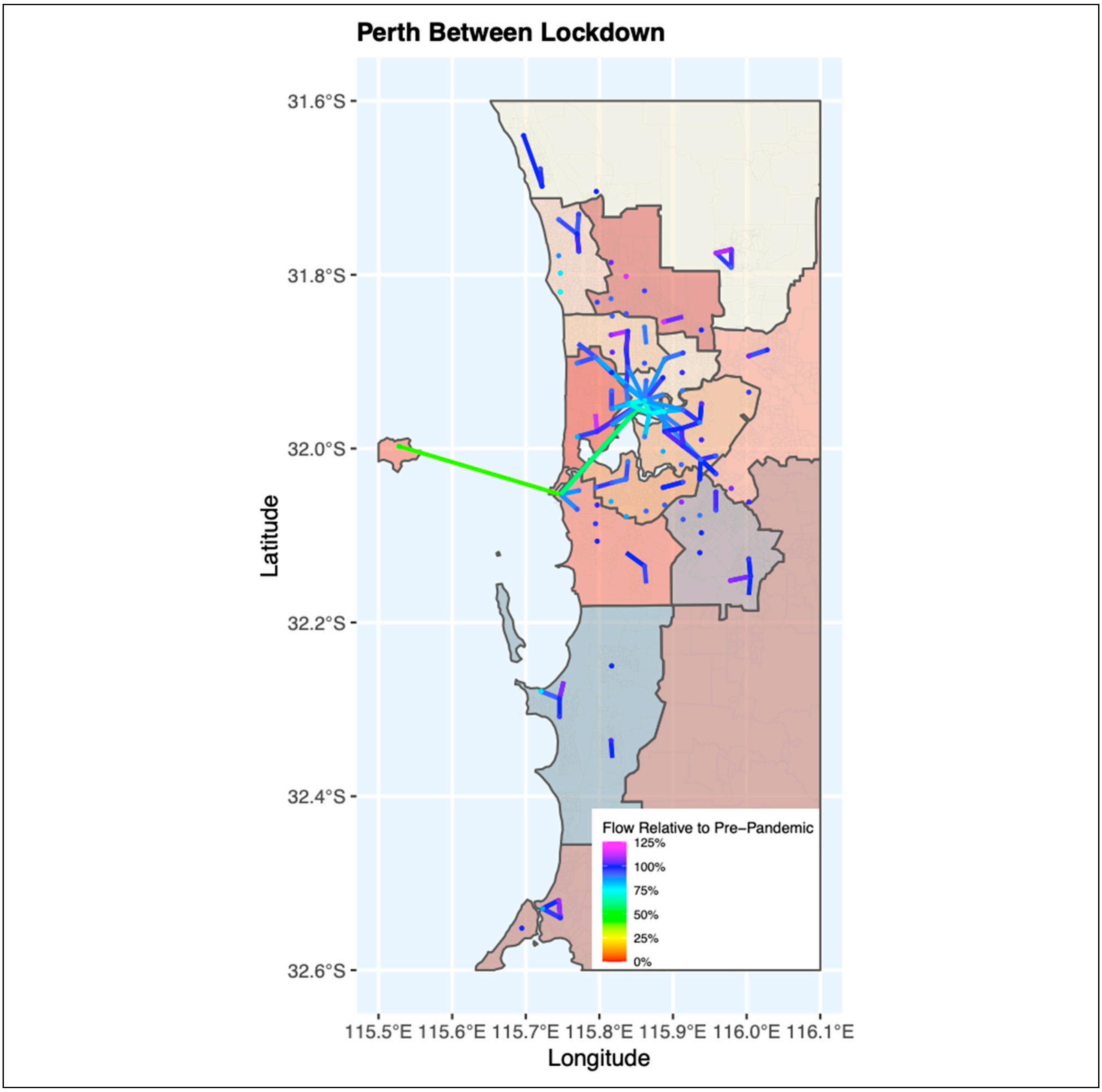
Perth Between Lockdown
31.6°S
31.8°S
32.0°S
32.2°S
32.4°S
32.6°S
Latitude
115.5°E 115.6°E 115.7°E 115.8°E 115.9°E 116.0°E 116.1°E
Longitude
Flow Relative to Pre−Pandemic
125%
100%
75%
50%
25%
0%

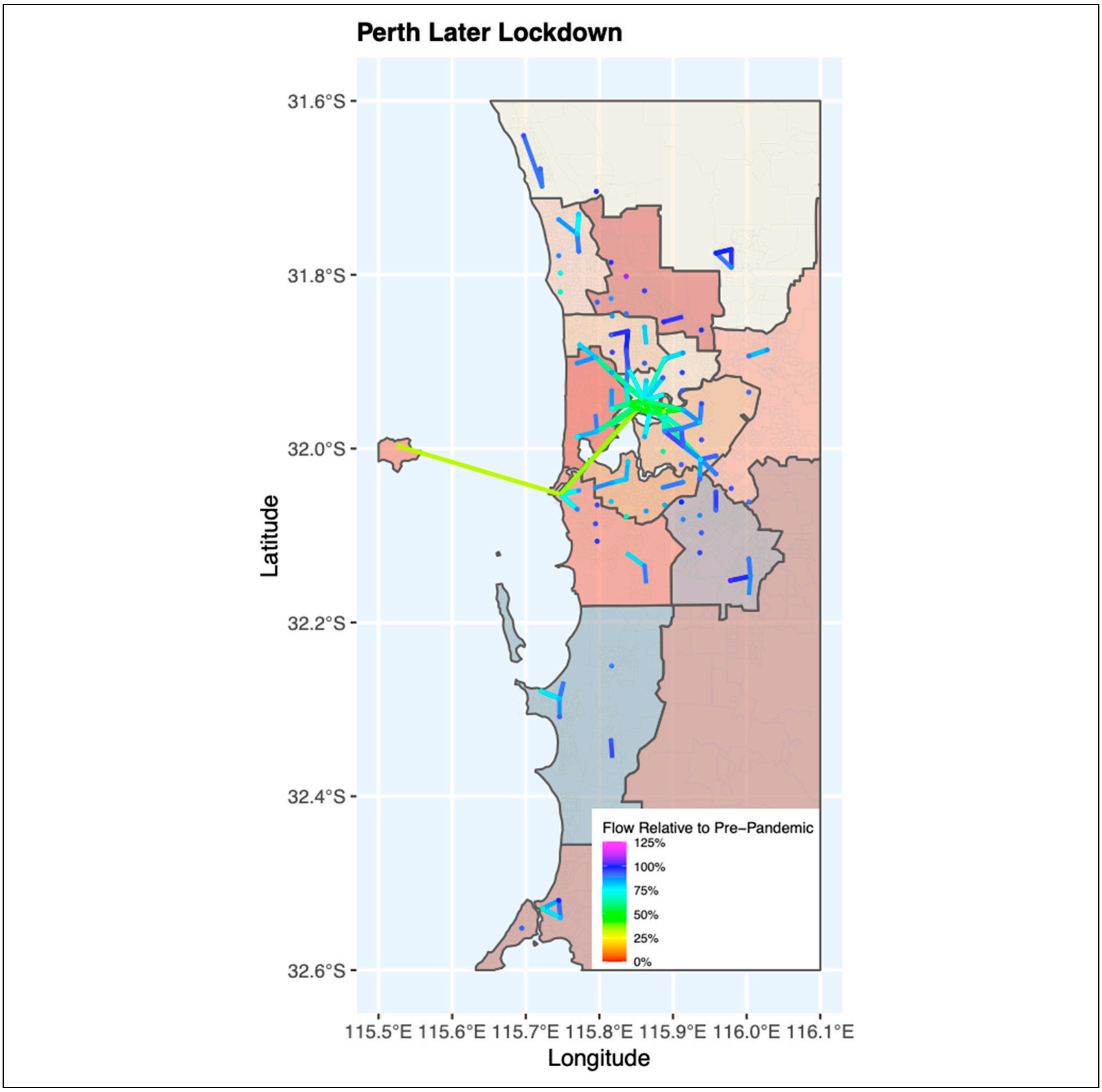
Perth Later Lockdown
31.6°S
31.8°S
32.0°S
32.2°S
32.4°S
32.6°S
Latitude
115.5°E 115.6°E 115.7°E 115.8°E 115.9°E 116.0°E 116.1°E
Longitude
Flow Relative to Pre−Pandemic
125%
100%
75%
50%
25%
0%

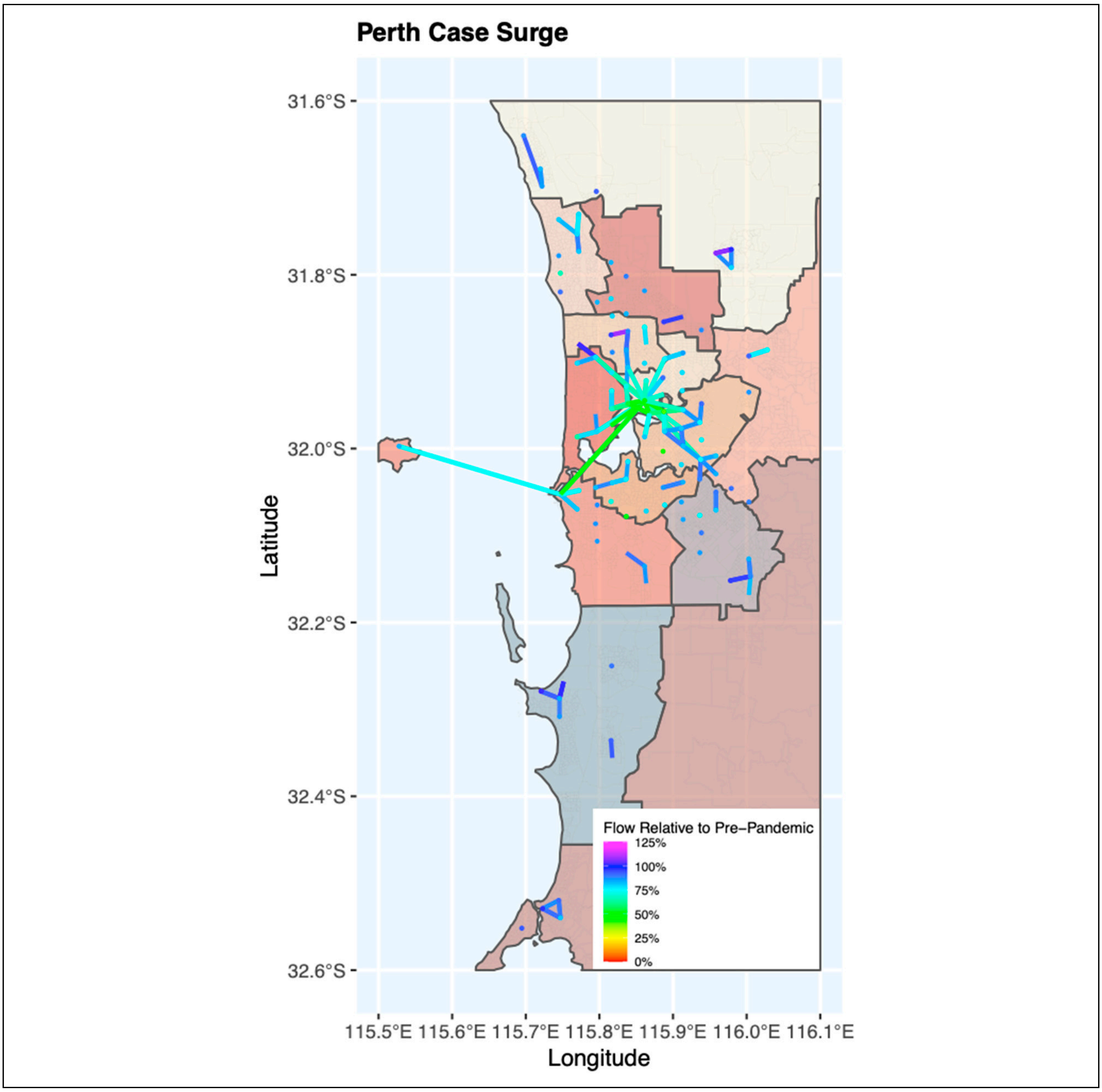
Perth Case Surge
31.6°S
31.8°S
32.0°S
32.2°S
32.4°S
32.6°S
Latitude
115.5°E 115.6°E 115.7°E 115.8°E 115.9°E 116.0°E 116.1°E
Longitude
Flow Relative to Pre−Pandemic
125%
100%
75%
50%
25%
0%

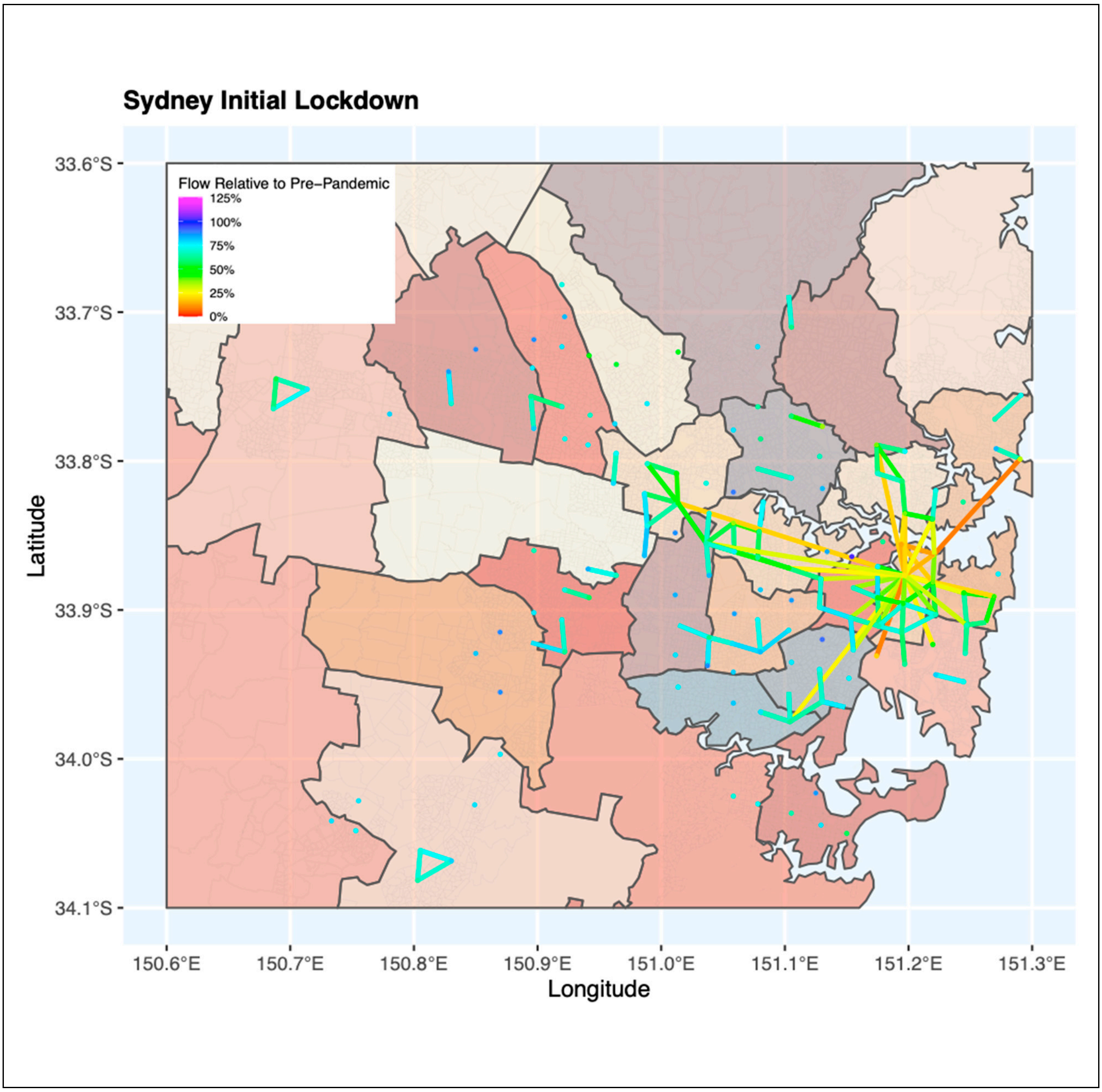
Sydney Initial Lockdown
Flow Relative to Pre–Pandemic
125%
100%
75%
50%
25%
0%
33.6°S
33.7°S
33.8°S
33.9°S
34.0°S
34.1°S
Latitude
150.6°E
150.7°E
150.8°E
150.9°E
151.0°E
151.1°E
151.2°E
151.3°E
Longitude

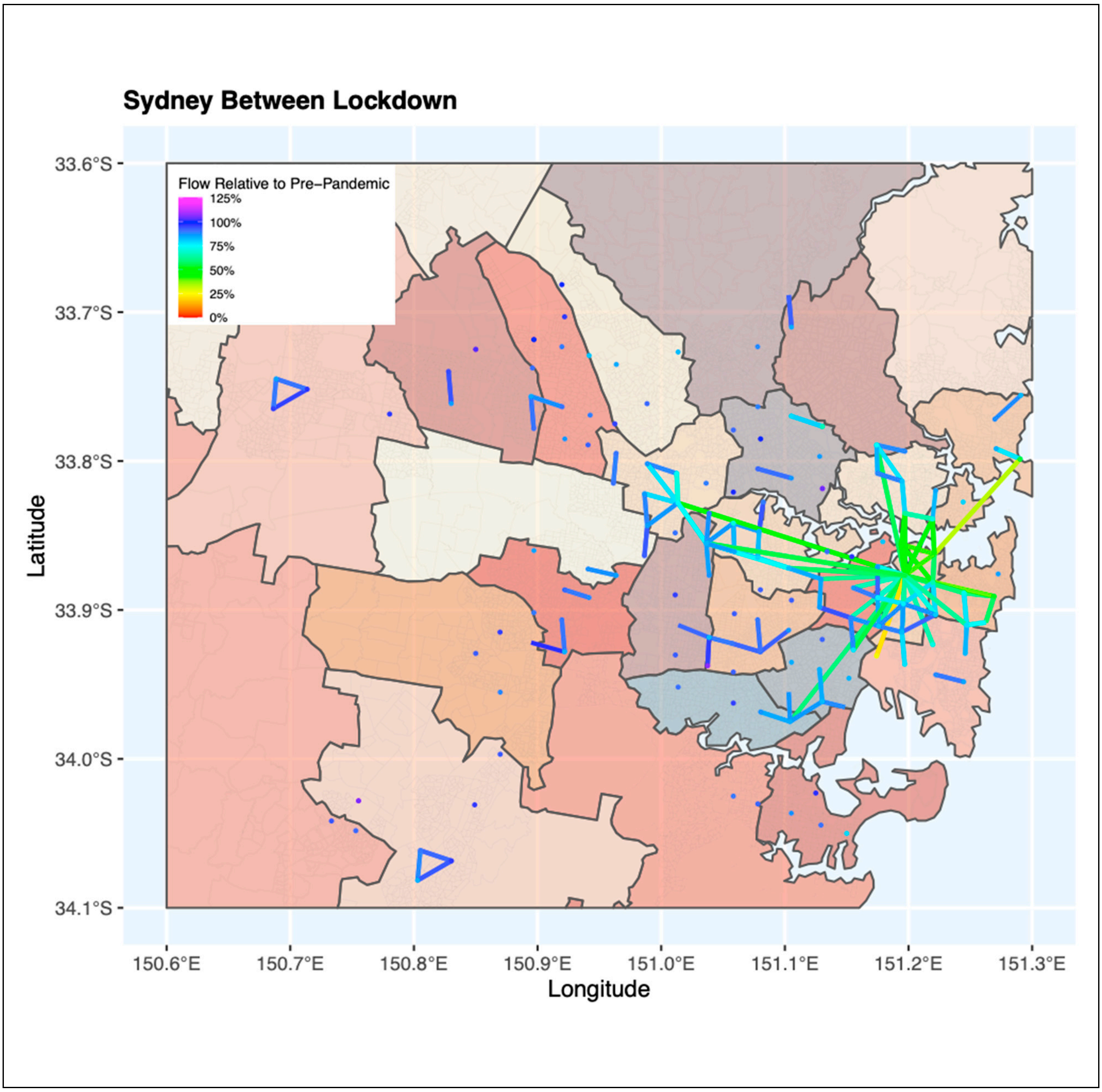
Sydney Between Lockdown
Flow Relative to Pre−Pandemic
125%
100%
75%
50%
25%
0%
33.6°S
33.7°S
33.8°S
33.9°S
34.0°S
34.1°S
Latitude
150.6°E
150.7°E
150.8°E
150.9°E
151.0°E
151.1°E
151.2°E
151.3°E
Longitude

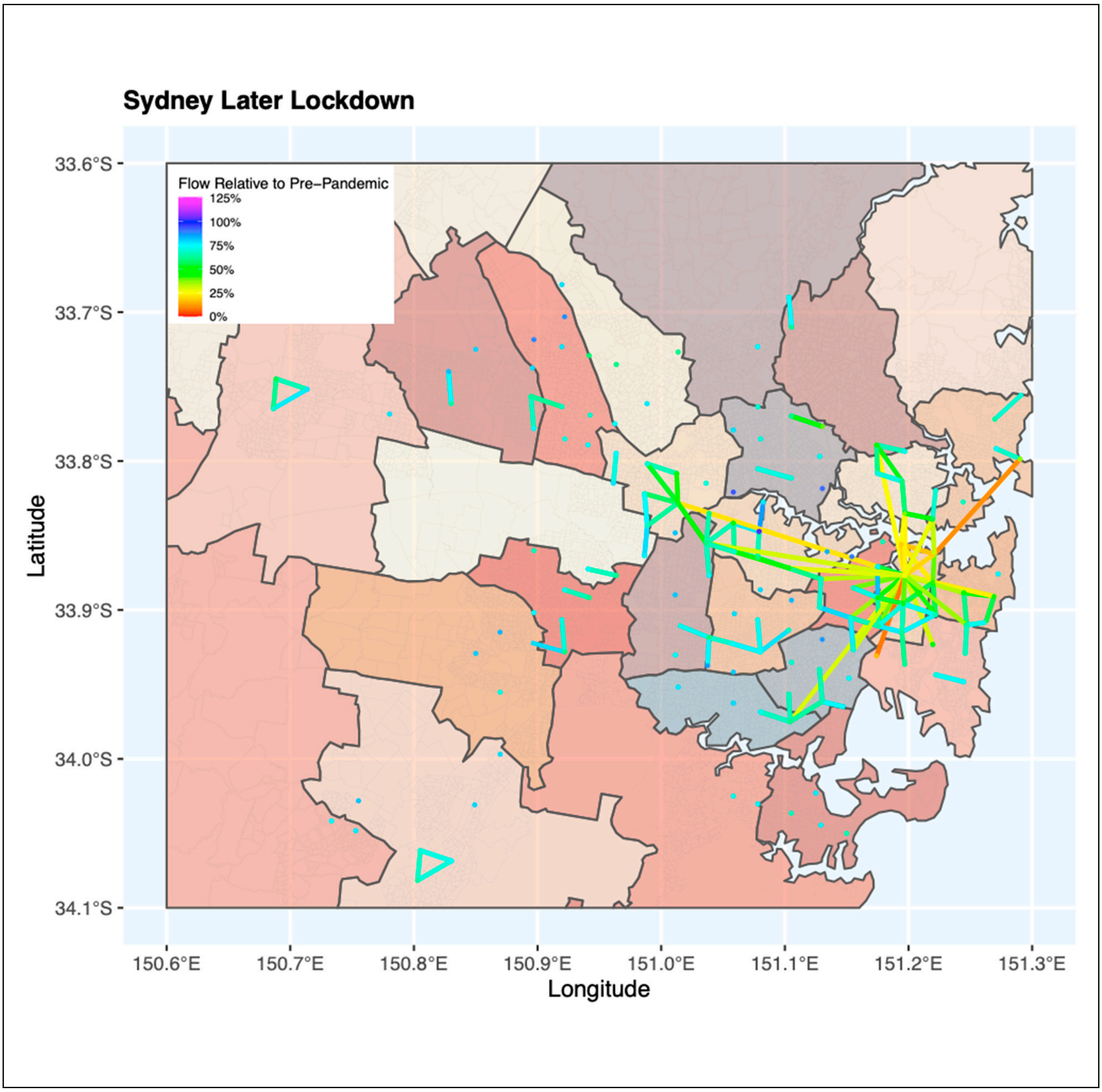
Sydney Later Lockdown
Flow Relative to Pre–Pandemic
125%
100%
75%
50%
25%
0%
33.6°S
33.7°S
33.8°S
33.9°S
34.0°S
34.1°S
Latitude
150.6°E
150.7°E
150.8°E
150.9°E
151.0°E
151.1°E
151.2°E
151.3°E
Longitude

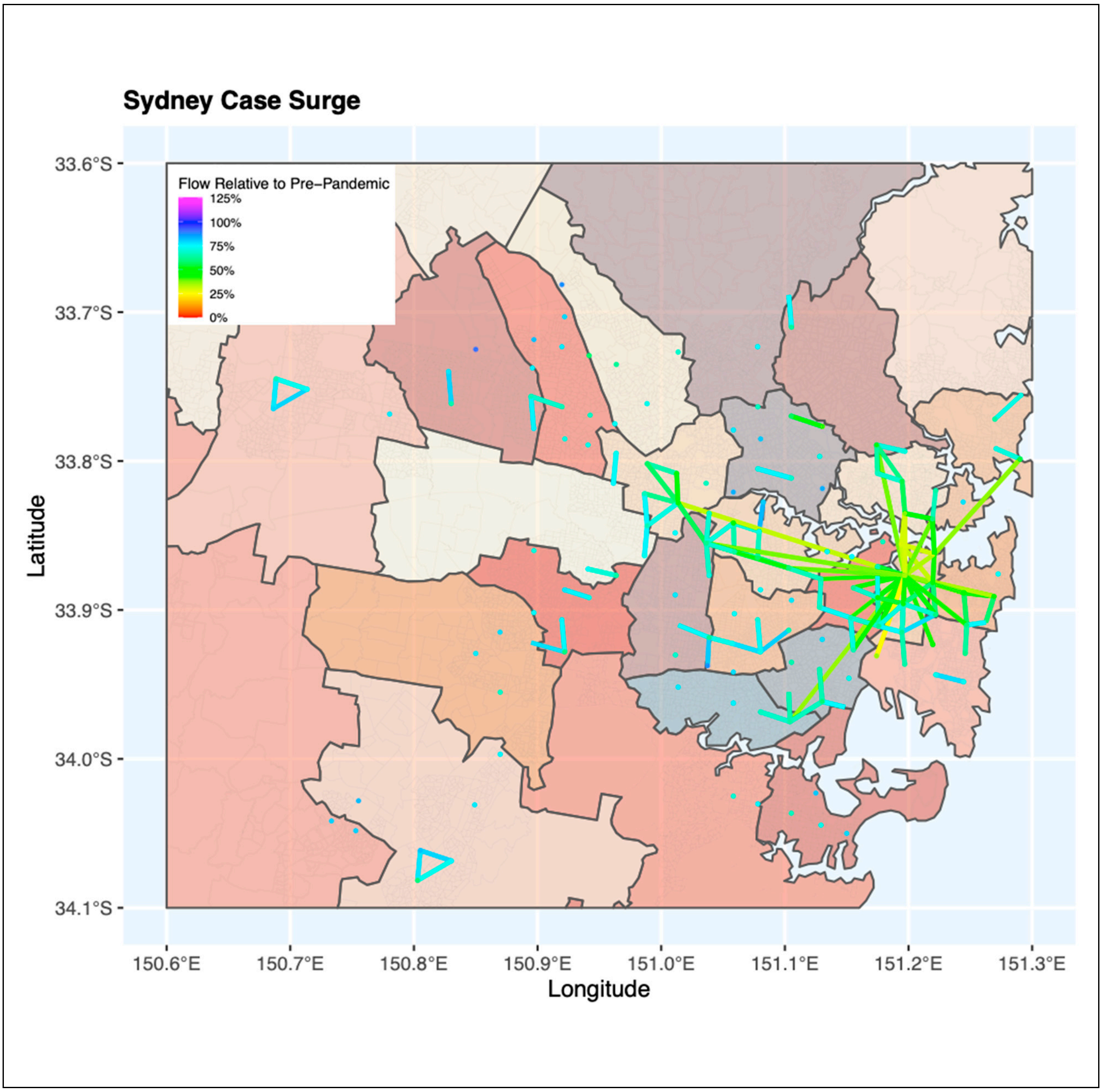

Figure S2. Movement maps for cities during pandemic stages relative to a pre-pandemic baseline for (a) The initial lockdown period, (b) Periods without movement restrictions, (c) Snap lockdowns, and (d) Surge in cases within a highly vaccinated population. For interpretability, only flows that were within the 1% of flow volume on any week within the timeseries are mapped.

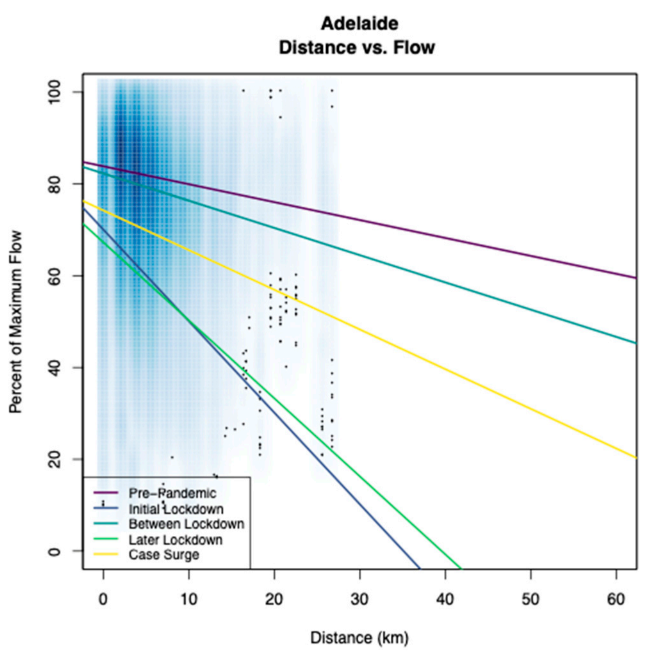
Adelaide
Distance vs. Flow
Percent of Maximum Flow
Distance (km)
Pre−Pandemic
Initial Lockdown
Between Lockdown
Later Lockdown
Case Surge
0
20
40
60
80
100
0
10
20
30
40
50
60

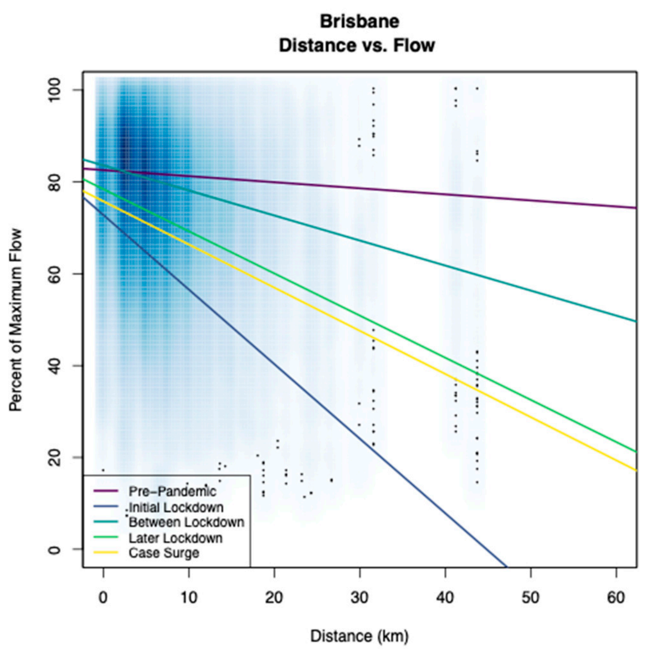
Brisbane
Distance vs. Flow
Percent of Maximum Flow
Distance (km)
Pre−Pandemic
Initial Lockdown
Between Lockdown
Later Lockdown
Case Surge
0
20
40
60
80
100
0
10
20
30
40
50
60

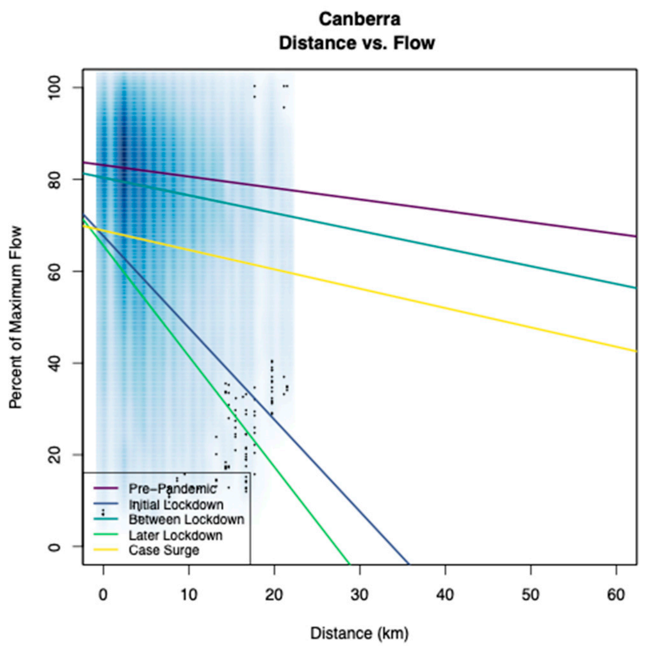
Canberra
Distance vs. Flow
Percent of Maximum Flow
Distance (km)
Pre−Pandemic
Initial Lockdown
Between Lockdown
Later Lockdown
Case Surge

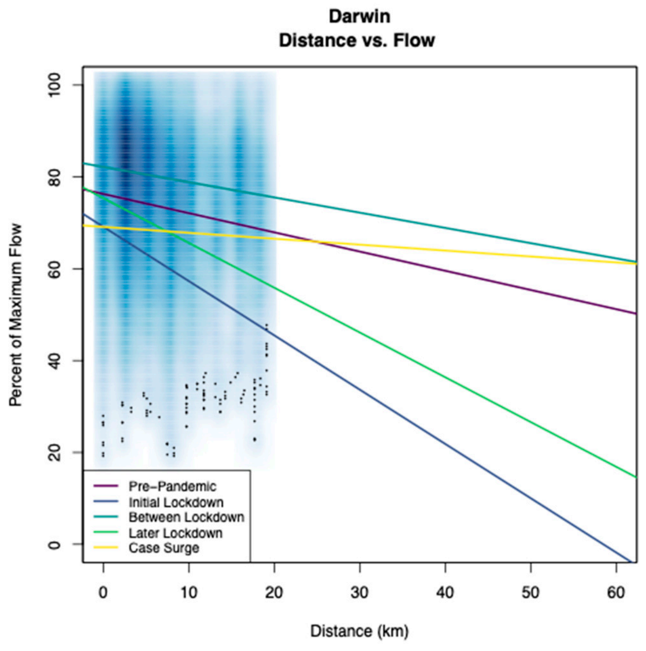
Darwin
Distance vs. Flow
Percent of Maximum Flow
Distance (km)
Pre−Pandemic
Initial Lockdown
Between Lockdown
Later Lockdown
Case Surge

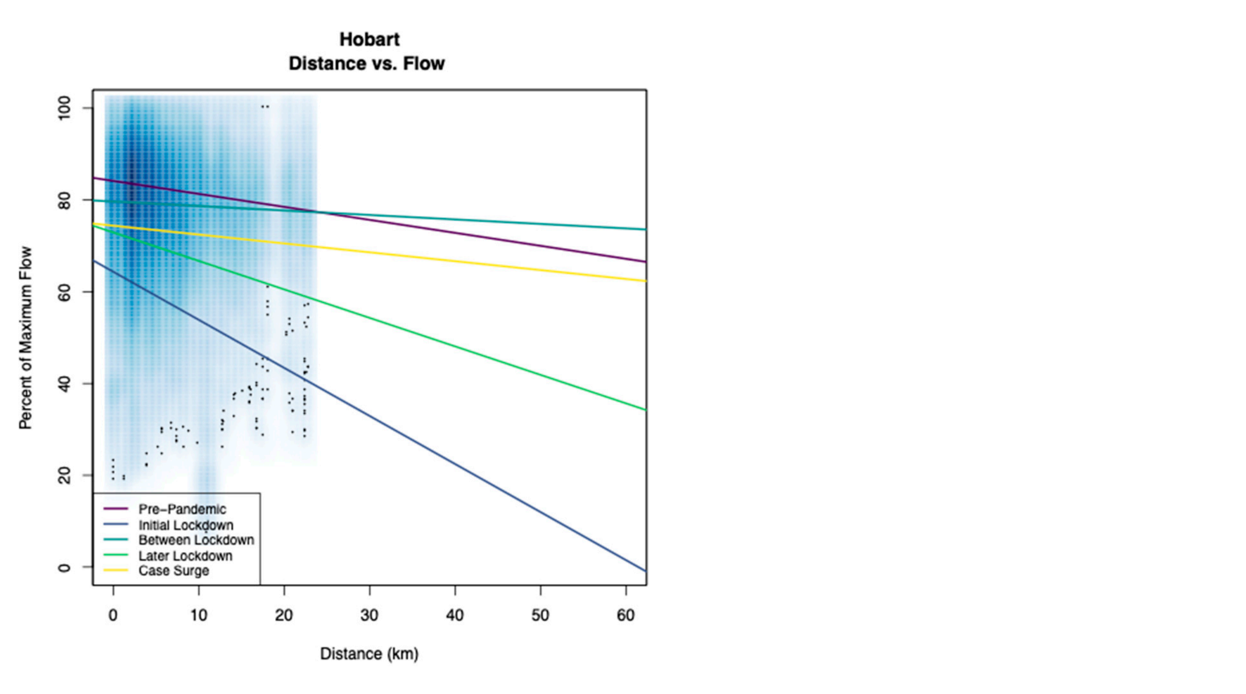
Hobart
Distance vs. Flow
Percent of Maximum Flow
Distance (km)
Pre−Pandemic
Initial Lockdown
Between Lockdown
Later Lockdown
Case Surge
0
20
40
60
80
100
0
10
20
30
40
50
60

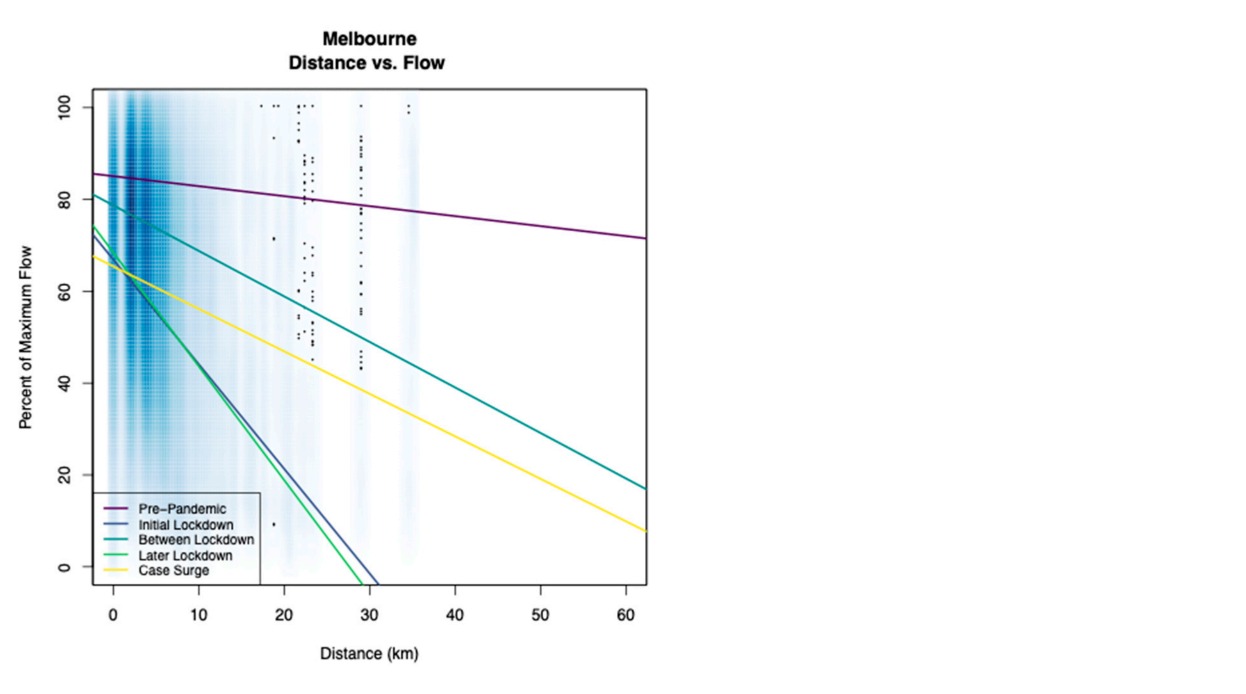
Melbourne
Distance vs. Flow
Percent of Maximum Flow
Distance (km)
Pre−Pandemic
Initial Lockdown
Between Lockdown
Later Lockdown
Case Surge
0
20
40
60
80
100
0
10
20
30
40
50
60

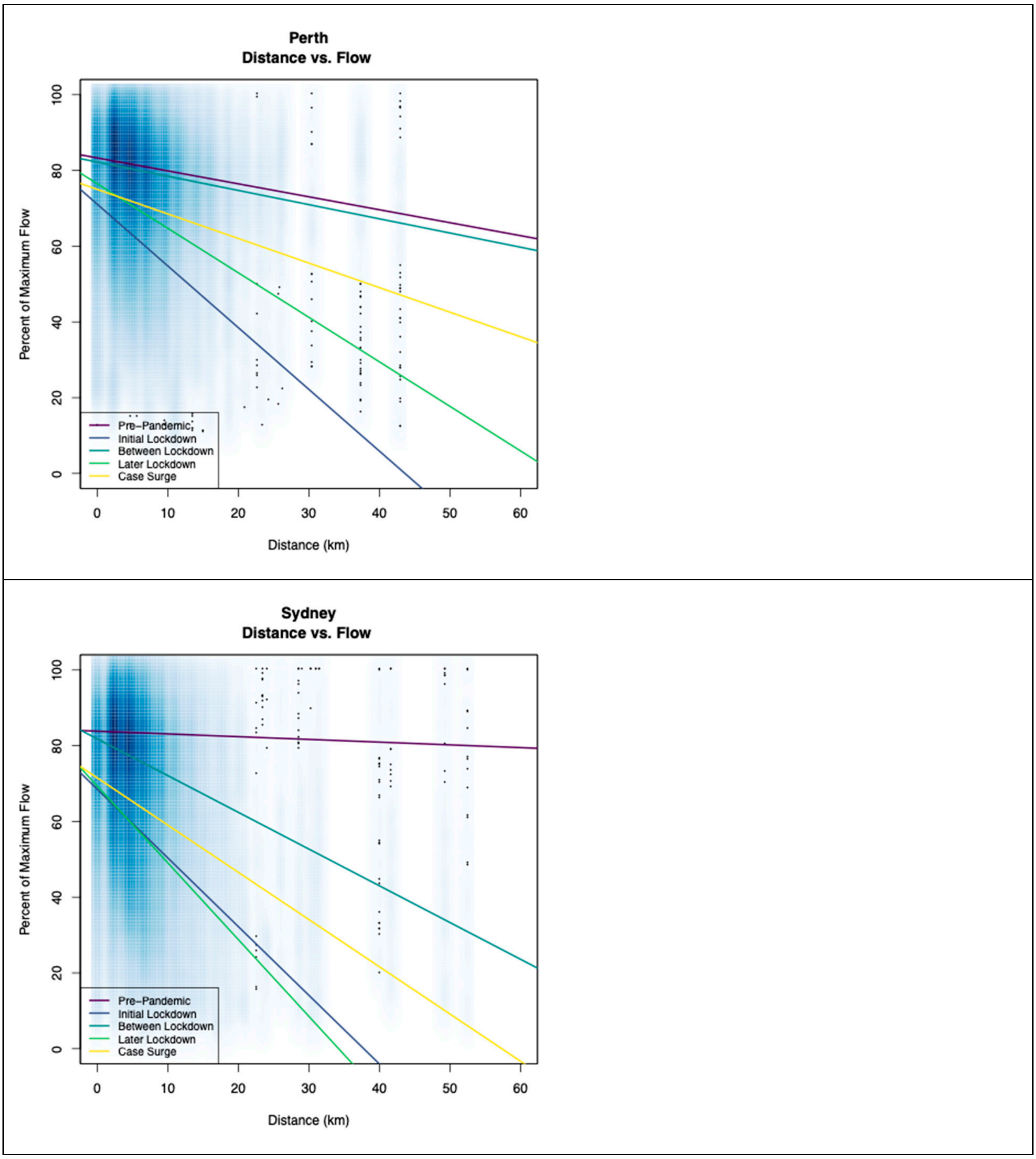

Figure S3. Declines in human movement flow volume by city for all lockdown categories for a single city. The lines represent the linear trends for all weeks within each category.

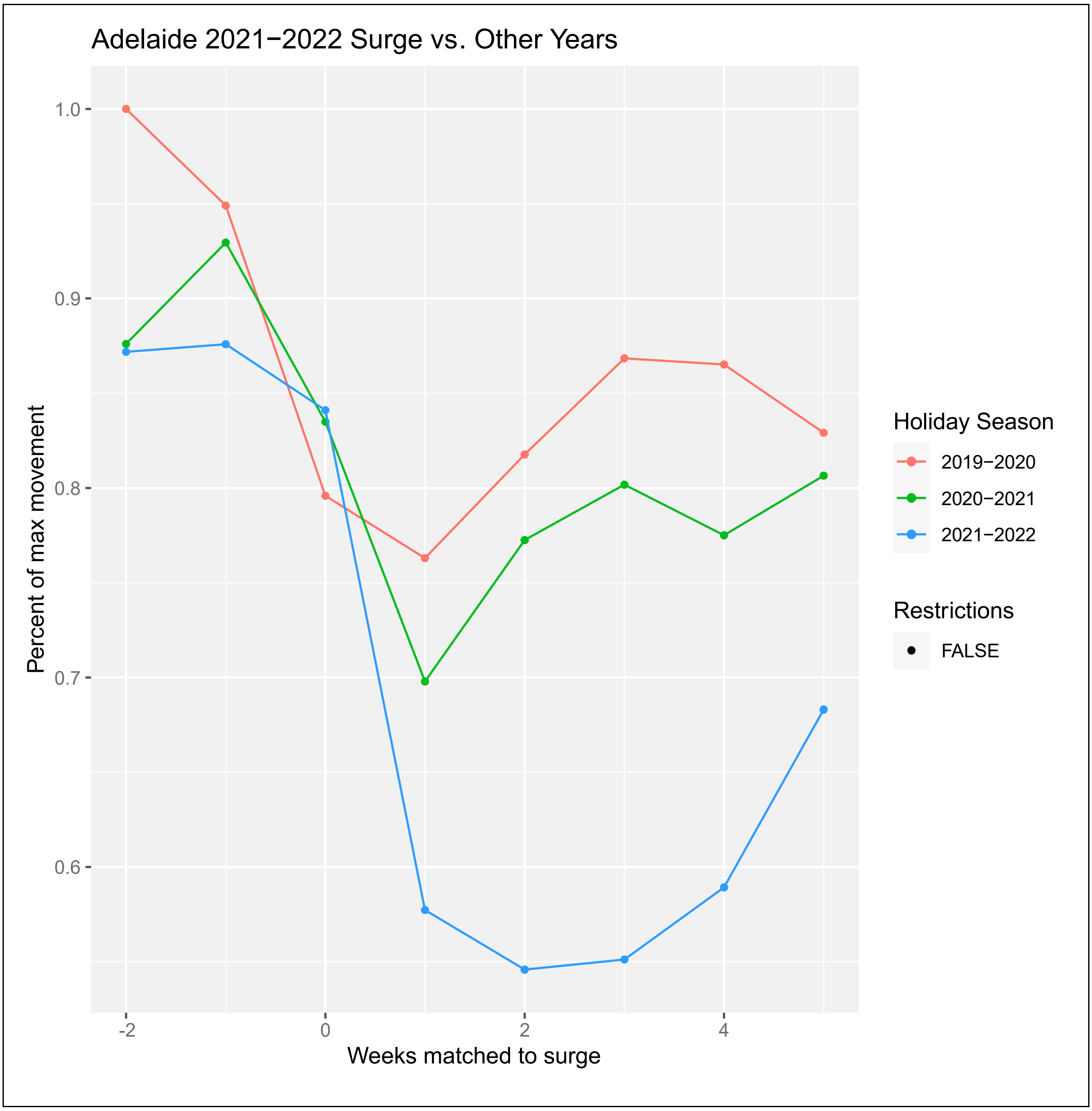
Adelaide 2021−2022 Surge vs. Other Years
Percent of max movement
1.0
0.9
0.8
0.7
0.6
-2
0
2
4
Weeks matched to surge
Holiday Season
2019−2020
2020−2021
2021−2022
Restrictions
FALSE

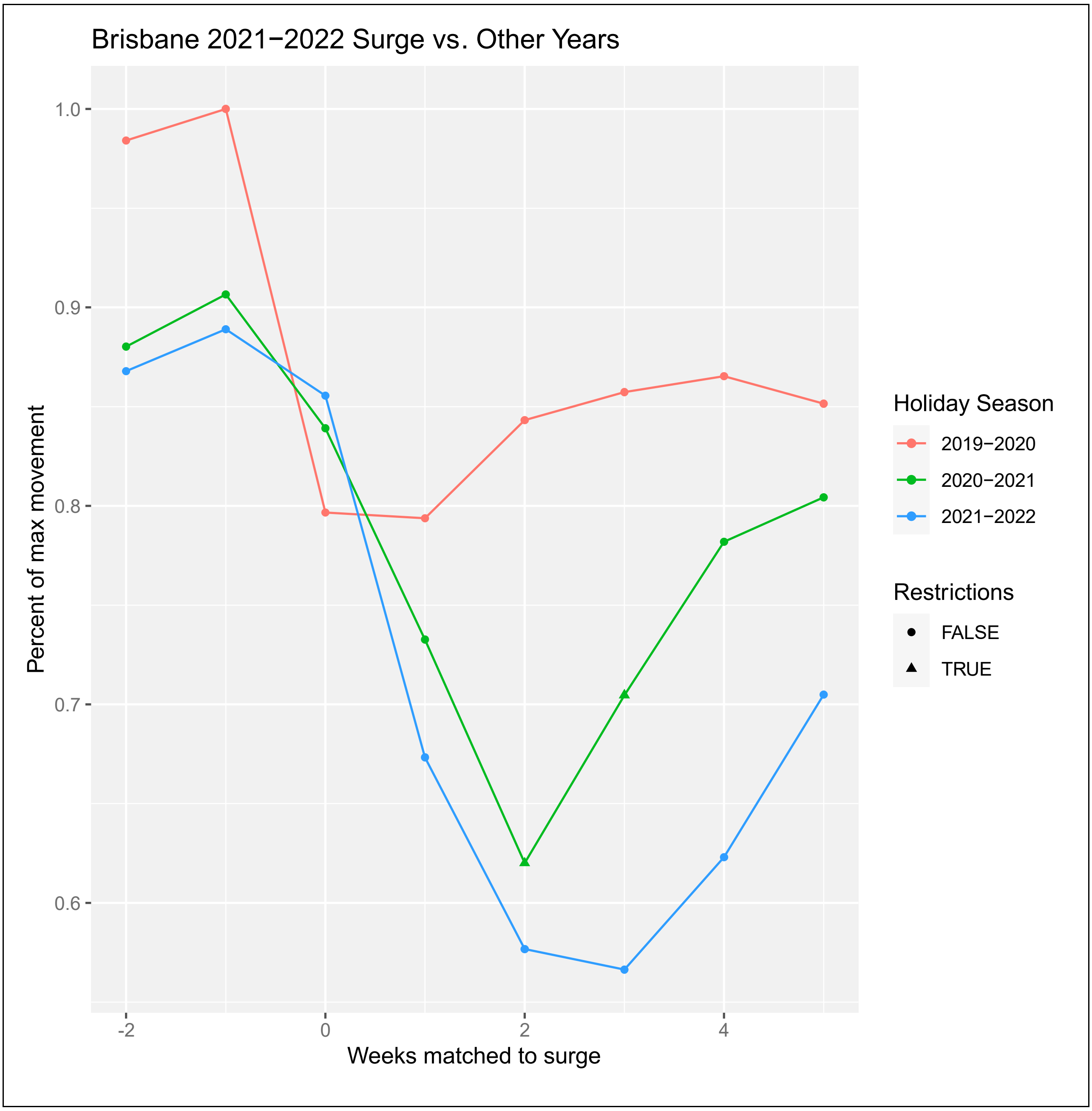
Brisbane 2021−2022 Surge vs. Other Years
Percent of max movement
1.0
0.9
0.8
0.7
0.6
-2
0
2
4
Weeks matched to surge
Holiday Season
2019−2020
2020−2021
2021−2022
Restrictions
FALSE
TRUE

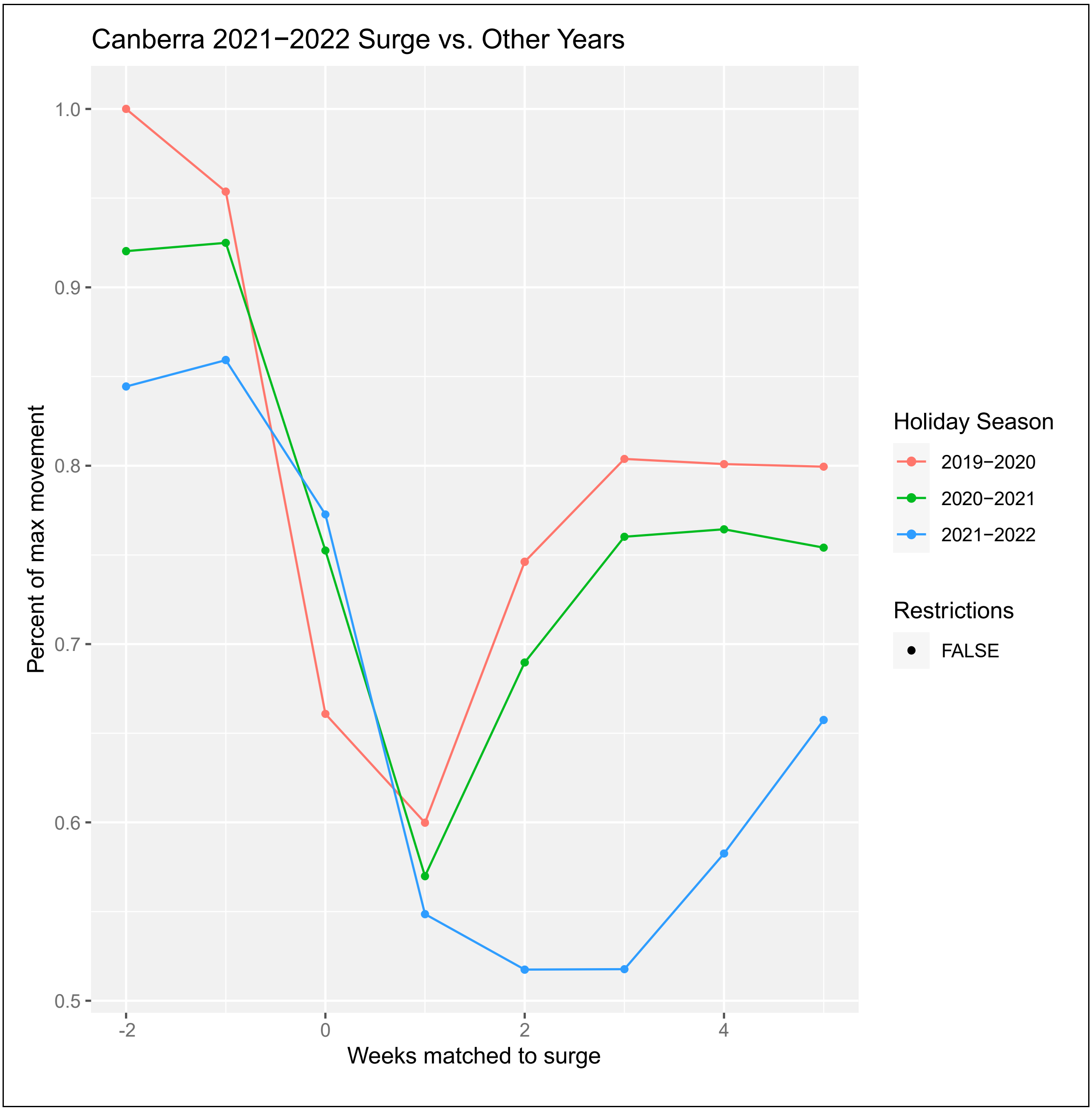
Canberra 2021−2022 Surge vs. Other Years
Percent of max movement
1.0
0.9
0.8
0.7
0.6
0.5
-2
0
2
4
Weeks matched to surge
Holiday Season
2019−2020
2020−2021
2021−2022
Restrictions
FALSE

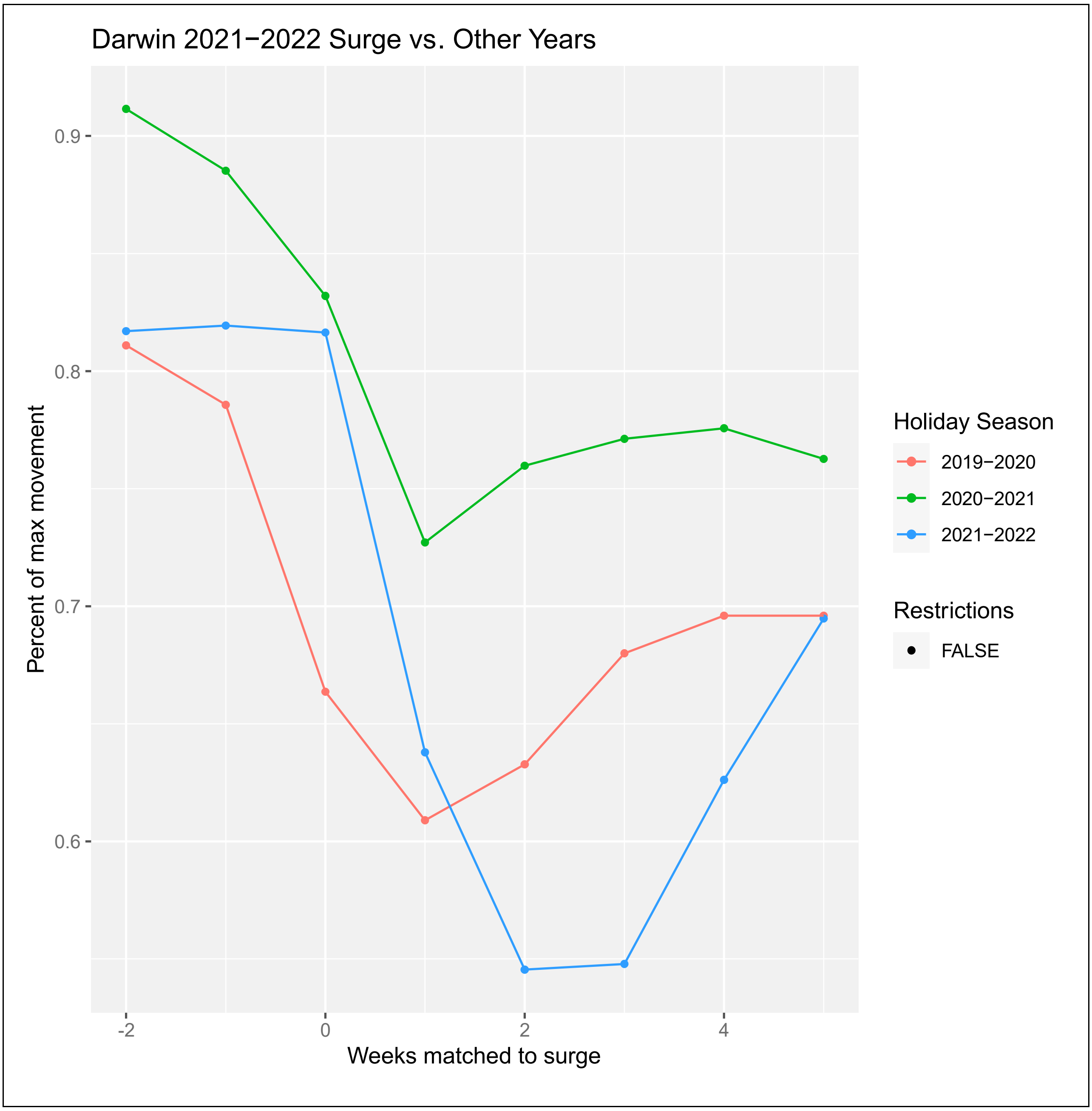
Darwin 2021−2022 Surge vs. Other Years
Percent of max movement
0.9
0.8
0.7
0.6
-2
0
2
4
Weeks matched to surge
Holiday Season
2019−2020
2020−2021
2021−2022
Restrictions
FALSE

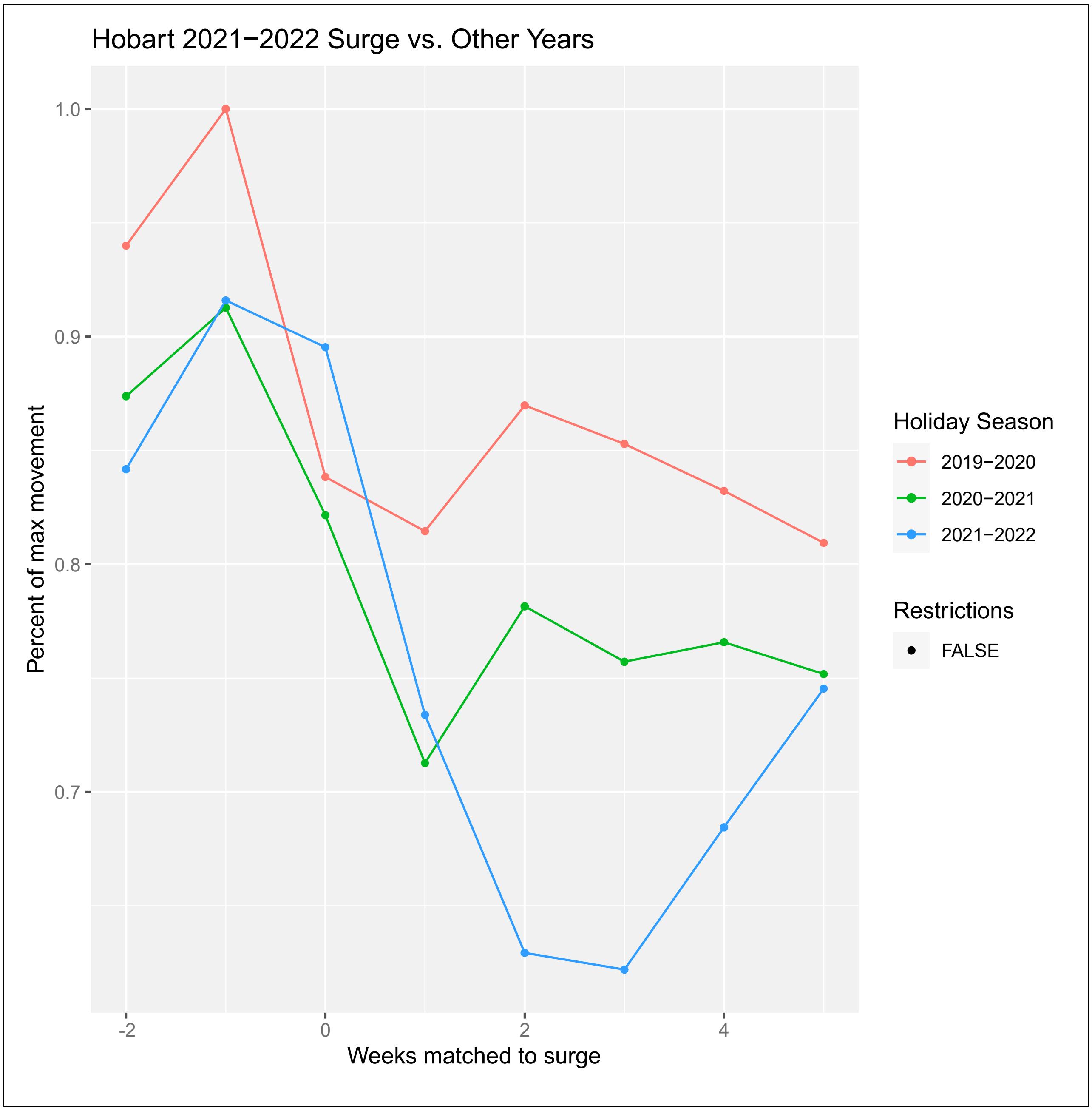
Hobart 2021−2022 Surge vs. Other Years
Percent of max movement
1.0
0.9
0.8
0.7
-2
0
2
4
Weeks matched to surge
Holiday Season
2019−2020
2020−2021
2021−2022
Restrictions
FALSE

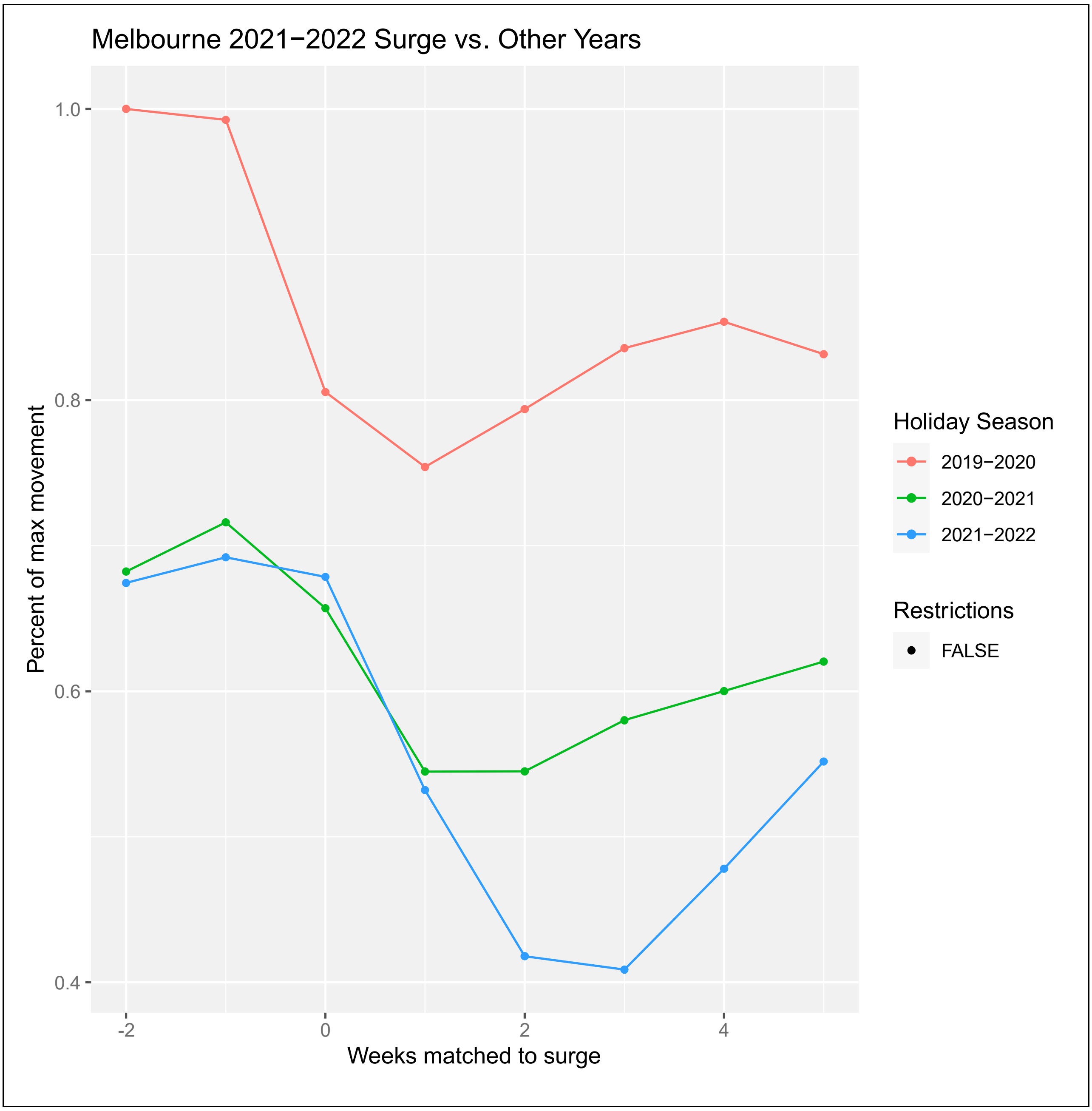
Melbourne 2021−2022 Surge vs. Other Years
Percent of max movement
1.0
0.8
0.6
0.4
-2
0
2
4
Weeks matched to surge
Holiday Season
2019−2020
2020−2021
2021−2022
Restrictions
FALSE

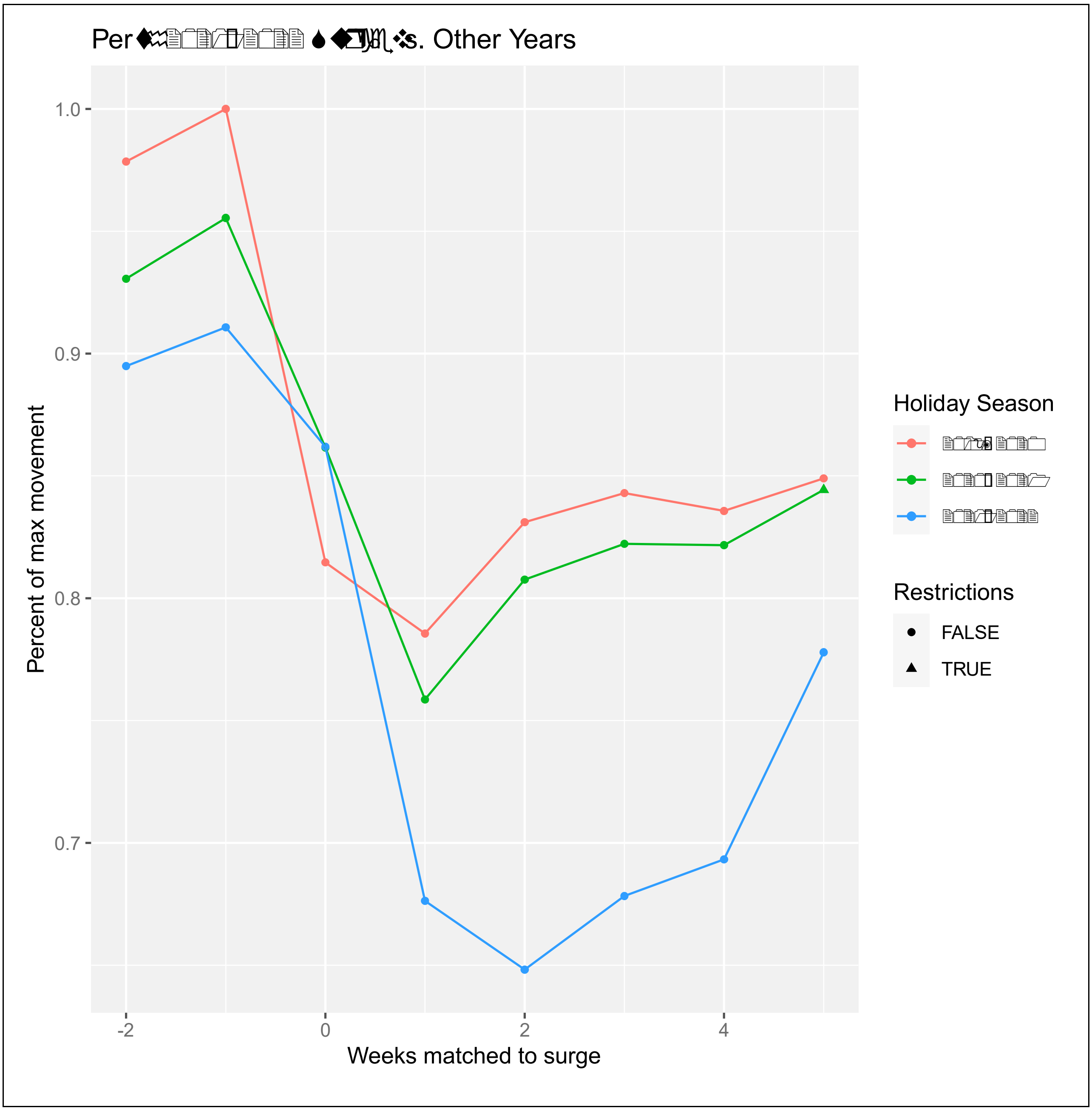
Other Years
1.0
0.9
0.8
0.7
Percent of max movement
-2
0
2
4
Weeks matched to surge
Holiday Season
Restrictions
FALSE
TRUE

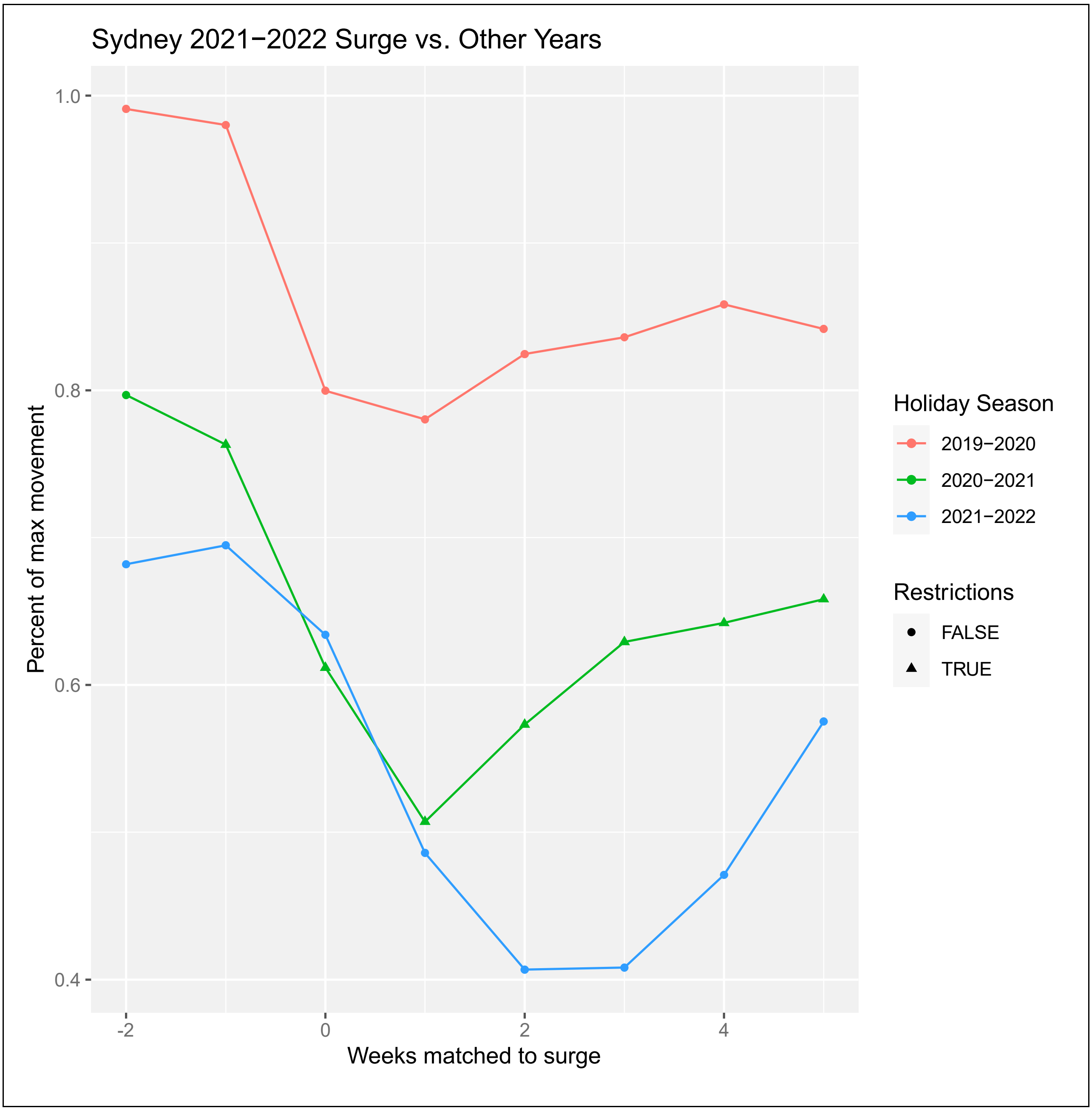

Figure S4. Declines in human movement flow volume coincident with the late pandemic case surges amongst a heavily vaccinated population and without restrictions compared to observed holiday movement volumes in other years. Anniversary weeks are aligned for each plot.
